# Oxidation of protein-bound methionine in Photofrin-photodynamic therapy-treated human tumor cells explored by methionine-containing peptide enrichment and quantitative proteomics approach

**DOI:** 10.1038/s41598-017-01409-9

**Published:** 2017-05-02

**Authors:** Ya-Ju Hsieh, Kun-Yi Chien, I-Fang Yang, I-Neng Lee, Chia-Chun Wu, Tung-Yung Huang, Jau-Song Yu

**Affiliations:** 1 Molecular Medicine Research Center, College of Medicine, Chang Gung University, Taoyuan, Taiwan; 2 Graduate Institute of Biomedical Sciences, College of Medicine, Chang Gung University, Taoyuan, Taiwan; 3 Department of Biochemistry and Molecular Biology, College of Medicine, Chang Gung University, Taoyuan, Taiwan; 4 Clinical Proteomics Core Laboratory, Chang Gung Memorial Hospital, Taoyuan, Taiwan; 5 Department of Medical Research, Chang Gung Memorial Hospital, Chia-Yi, Taiwan; 6 Liver Research Center, Chang Gung Memorial Hospital, Linkou, Taiwan

## Abstract

In Photofrin-mediated photodynamic therapy (PDT), cell fate can be modulated by the subcellular location of Photofrin. PDT triggers oxidative damage to target cells, including the methionine (Met) oxidation of proteins. Here, we developed a new Met-containing peptide enrichment protocol combined with SILAC-based quantitative proteomics, and used this approach to explore the global Met oxidation changes of proteins in PDT-treated epidermoid carcinoma A431 cells preloaded with Photofrin at the plasma membrane, ER/Golgi, or ubiquitously. We identified 431 Met-peptides corresponding to 302 proteins that underwent severe oxidation upon PDT and observed overrepresentation of proteins related to the cell surface, plasma membrane, ER, Golgi, and endosome under all three conditions. The most frequently oxidized Met-peptide sequence was “QAMXXM**M-**E/G/M-S/G-A/G/F-XG”. We also identified several hundred potential Photofrin-binding proteins using affinity purification coupled with LC-MS/MS, and confirmed the bindings of EGFR and cathepsin D with Photofrin. The enzyme activities of both proteins were significantly reduced by Photofrin-PDT. Our results shed light on the global and site-specific changes in Met-peptide oxidation among cells undergoing Photofrin-PDT-mediated oxidative stress originating from distinct subcellular sites, and suggest numerous potential Photofrin-binding proteins. These findings provide new insight into the molecular targets through which Photofrin-PDT has diverse effects on target cells.

## Introduction

Photodynamic therapy (PDT) has been approved by the U.S. Food and Drug Administration (FDA) and other health agencies in many countries for the clinical management of various cancers^1^. Photofrin is the most widely used photosensitizer in clinical PDT, and was the first such drug approved by the FDA for cancer treatment. In PDT, cells or tissues are exposed to a photosensitizing drug (a non-toxic dye), harmless visible light, and oxygen to produce highly reactive oxygen species(ROS) that cause tumor destruction^1^. More specifically, the light-stimulated photosensitizer reacts directly with biological substances and/or transfers energy to oxygen to generate singlet oxygen. This highly reactive ROS attacks many biological molecules, including lipids^2^, proteins^3^, and nucleic acids^4^, to cause cell death^1, 5–7^. PDT can induce different cell fates depending on the cell type^8–10^, the utilized photosensitizer, the treated subcellular site^11–14^, and the total administered dose^15, 16^. All these factors are interdependent^17^.

For Photofrin-mediated PDT, the subcellular location of the agent strongly influences the cell death response. We previously showed that Photofrin is dynamically distributed in human epidermoid carcinoma A431 cells treated with the agent in the medium: it is initially localized at the plasma membrane, but prolonged incubation allows it to enter the ER/Golgi^15^. We further showed that PDT with plasma-membrane-targeted Photofrin induces necrosis-like cell death, whereas that with ER/Golgi-localized Photofrin triggers the formation of perinuclear vacuoles via SERCA dysfunction and is highly correlated with the location of the ROS generated by the treated cells^16^. Thus, distinct signaling events appear to be triggered when different parts of the cell are subjected to the oxidative stress elicited by Photofrin-mediated PDT. Protein oxidation exerts diverse biological effects and is a major molecular consequence of the PDT-induced generation of intracellular ROS. Therefore, PDT of human tumor cells preloaded with Photofrin at different subcellular sites appears to offer an excellent opportunity to study the protein oxidation events that occur in different subcellular locations subjected to oxidative stress. However, no previous study has examined the global or site-specific protein oxidation of PDT-exposed cells in which the photosensitizer has been dispensed to distinct subcellular locations.

Protein oxidation has both positive and negative consequences for various biological processes, including receptor activation^18^, signal transduction and gene expression^19^, apoptosis^20^, the antimicrobial and cytotoxic actions of immune cells^21^, aging^22^, and age-related degenerative diseases^23^. Proteins are among the major biomolecules targeted by ROS in cells; among the constituents of a protein, the most readily oxidized amino acid is methionine (Met), which can be attacked by various ROS, including H_2_O_2_, hydroxyl radicals, singlet oxygen, etc.^24, 25^. Oxidation of Met residues can alter the protein structure, leading to the loss of enzyme activity and/or protein-protein interaction properties, as seen for calmodulin^26^, HIV-2 protease^27^, and alpha-1 antitrypsin^28^. Another example is caspase-3, as we previously showed that Photofrin-PDT oxidizes the Met residues of procaspase-3 and impairs its activation^29^.

Since Met residues are highly susceptible to being oxidized by various types of ROS, several groups have sought to characterize and/or quantify the Met oxidation of proteins. Oien *et al*.^30^ generated polyclonal antibodies against oxidized Met residues, but the applicability of this strategy for the proteome-wide investigation of Met oxidation has not yet been explored. Rosen *et al*.^31^ used spectral counting to determine the percentage of Met oxidation, but did not consider the differences of ionization efficiency between oxidized and reduced Met-peptides. Gevaert *et al*.^32, 33^ reported a COFRADIC (combined fractional diagonal chromatography) proteomics technology for the identification and quantification of the Met oxidation of proteins. However, this approach requires numerous steps for peptide preparation and separation, and its success critically requires the skillful application of chromatographic techniques. Whereas the above-mentioned methodologies did not use enrichment protocols to analyze Met-peptides, Grunert *et al*.^34^ applied a solid-phase reagent (porous glass beads carrying a bromo-acetyl group on the terminal end of a polymeric spacer; Pierce) to enrich the reduced form of Met-peptides for subsequent analysis by MALDI-TOF/MS. The authors provided evidence that the process removed the oxidized Met-peptides, but they assessed only model proteins. The use of this technique for proteome-wide analysis has been very limited^35, 36^.

In the present study, we developed an efficient Met-peptide enrichment methodology based on the selective reaction of iodoacetyl-PEG2-biotin with reduced Met-peptides under acidic conditions, as originally described by Gygi *et al*.^37^ for Cys-peptide enrichment. We applied this enrichment method in conjunction with SILAC (stable isotope labeling by amino acids in cell culture) technology and used on-line 2D-LC-MS/MS to quantify global changes of Met oxidation in human tumor cells subjected to Photofrin-PDT-induced oxidative stress at various subcellular locations. Using this method, we detected and quantified a significant number of Met-peptides that were not detected by shotgun proteomics approach without enrichment in PDT-treated cells. We observed that the distribution of the severely oxidized proteins was well correlated with that of Photofrin. We identified 803 potential Photofrin-binding proteins in human cells, and found that 94 of them were severely oxidized in PDT-treated cells. Our data reveal for the first time the profile of Met oxidation that occurs at distinct subcellular sites of living cells subjected to Photofrin-PDT-induced oxidative stress. The developed methodology can be widely and systematically applied to other oxidative-stress-related studies.

## Results

### Met-containing peptide enrichment by iodoacetyl-PEG2-biotin

**Figure 1:**
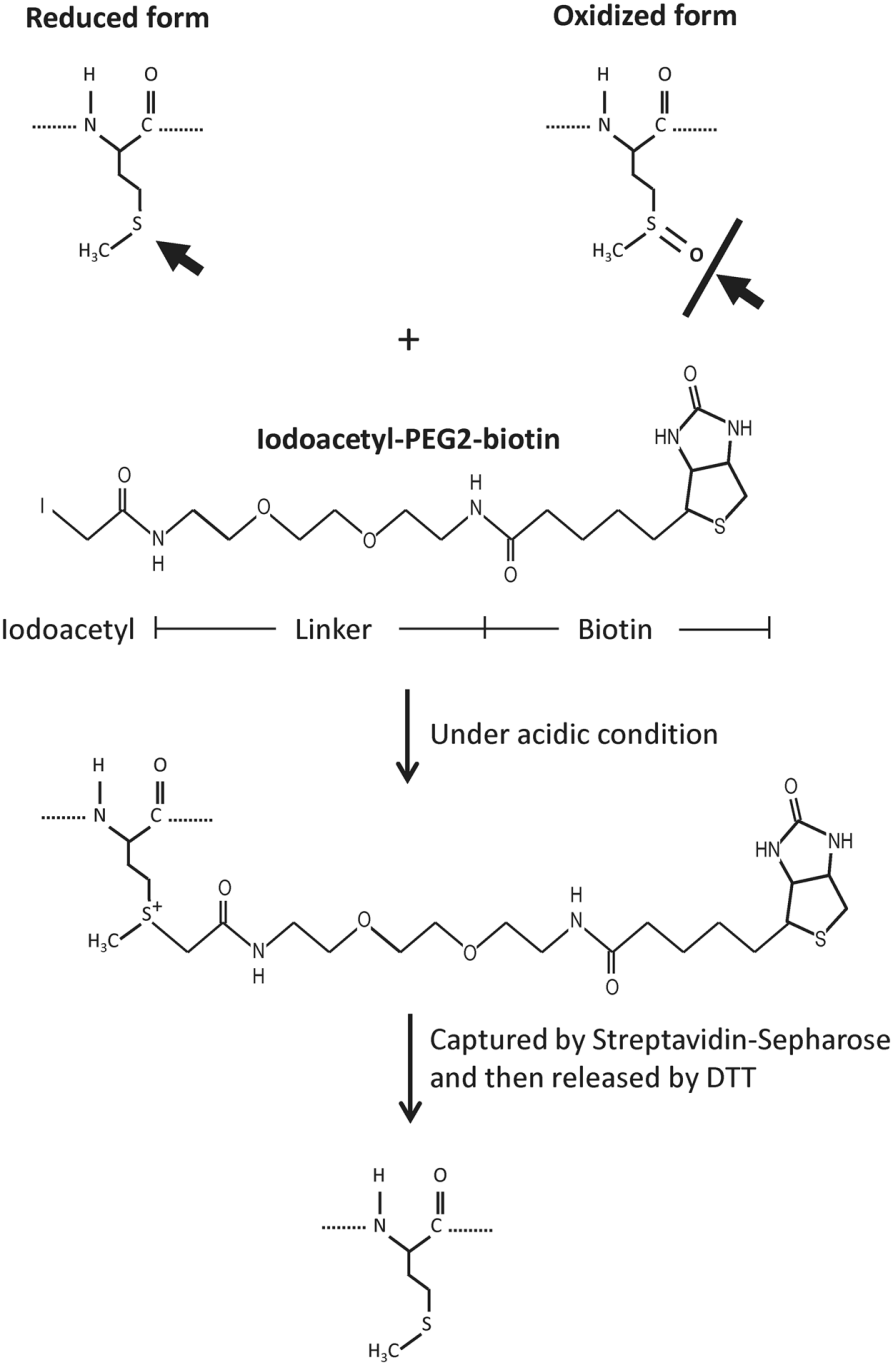
The scheme for Met-peptide enrichment. Reduced Met-peptides are labeled by the iodoacetyl-PEG2-biotin reagent under acidic conditions, whereas the oxidized Met-peptides are not. The labeled peptides are then captured and purified with streptavidin-Sepharose beads. After reduction by DTT, the captured Met-peptides are released from the beads and analyzed by LC-MS/MS.

Met-containing peptides can be alkylated by iodoacetate under acidic conditions^38^, whereas methionine sulfoxide (MetO)-containing peptides (the oxidized form) and Met-free peptides do not react with iodoacetate^34^. Therefore, the relative oxidation level of specific Met-residues can be deduced from the ratio of the reduced Met-peptide levels observed before and after oxidative stress. Here, we applied the well-established iodoacetyl-PEG2-biotin reagent, which was originally designed to capture cysteine-containing peptides, to capture the reduced Met-peptides in biological samples. We used the following protocol (Fig. 1): Cysteine residues were alkylated by iodoacetamide at neutral pH, and then acetic acid was used to decrease the pH, enabling the iodoacetyl group of theiodoacetyl-PEG2-biotin reagent to specifically alkylate the reduced Met-residues. The alkylated peptides were then selectively captured by streptavidin-conjugated beads, and the enriched Met-peptides were released and analyzed by on-line 2D-LC-MS/MS.

First of all, we designed an experiment to quantitatively evaluate the capture efficiency of this protocol for oxidized and reduced forms of a model Met-peptide, TVMENFVAFVDK, which is a tryptic peptide derived from bovine serum albumin (Fig. S1). This peptide was dimethylated by heavy (^13^CD_2_O) or light (CH_2_O) formaldehyde, and the resulting heavy peptide was oxidized by H_2_O_2_ to generate oxidized heavy peptide. Both oxidized (H) and reduced (L) peptides were purified by HPLC, quantified by LC-UV, equally mixed and then captured by the iodoacetyl-PEG2-biotin-based protocol. The enriched Met-peptides were released and analyzed by LC-MS/MS. A small portion of the equally mixed peptides was also analyzed by LC-MS/MS before the capture experiment. The results from this quantitative analysis clearly showed that (i) a very small percentage (~2.3%) of the oxidized Met-peptides can be captured as compared to its reduced form, which might result from the non-specific binding to streptavidin beads; and (ii) a small portion of the reduced (L) Met-peptides, as expected, become oxidized (to generate the oxidized (L) Met-peptides) during the enrichment procedure (see Fig. S1 for details).

Secondly, we evaluated the efficiency and reproducibility of this method to enrich Met-peptides derived from A431 cell lysates in triplicate experiments. As shown in Table 1, the percentage of Met-peptides increased from 31% (30.81%, 31.15% and 31.27%) to 96% (96.21%, 96.05% and 96.06%) after enrichment. The numbers of Met-peptides (18,205~18,612) detected using this enrichment method were much higher than those (11,099~11,460) observed without enrichment, and 78% (78.32%, 77.89% and 79.63%) of the enriched Met-peptides could be simultaneously found in the triplicate experiments. Collectively, the results indicate the high specificity, efficiency and reproducibility of iodoacetyl-PEG2-biotin-based protocol to enrich reduced Met-peptides from complex biological samples.

**Table 1:** Efficiency and reproducibility of the iodoacetyl-PEG2-biotin-based protocol to enrich Met-peptides derived from A431 cell lysates.

Peptide identification	Non-enriched			Enriched		
	Exp 1	Exp 2	Exp 3	Exp 1	Exp 2	Exp 3
No. of peptides	36022	36789	36428	19238	19377	18952
No. of peptides in all 3 exp.	25580			14982		
(%)	71.01	69.53	70.22	77.88	77.32	79.05
No. of non-Met-peptides	24923	25329	25037	730	765	747
No. of non-Met-peptides in all 3 exp.	17650			486		
(%)	70.82	69.68	70.50	66.58	63.53	65.06
No. of Met-peptides	11099	11460	11391	18508	18612	18205
No. of Met-peptides in all 3 exp.	7930			14496		
(%)	71.45	69.20	69.62	78.32	77.89	79.63
% of Met-peptides in all identified peptides	30.81	31.15	31.27	96.21	96.05	96.06

A431 cell lysates were digested by trypsin, and the digests were equally divided into three parts. For each part, the tryptic digests (200 μg protein) were subjected to Met-peptide enrichment using the iodoacetyl-PEG2-biotin-based protocol. After enrichment, one-fourth of the enriched product (equivalent to 50 μg protein of the original lysates) was analyzed by LC-MS/MS for peptide identification. The tryptic digests (10 μg protein) were directly analyzed by LC-MS/MS without prior enrichment for comparison purpose. “% of Met‐peptides” represents the percentage of Met‐peptides among the total identified peptides.

Furthermore, we also compared the efficiency of this newly developed protocol to that of the previously reported method (i.e. the use of bromoacetyl group-coupled beads^34^) for enriching Met-peptides derived from the lysates of PDT-treated A431 cells. Again, we could only detect ~31% of the Met-peptides in the trypsin-digested lysates of PDT-treated A431 cells without any enrichment process (Fig. S2a). After enrichment, the iodoacetyl-PEG2-biotin-based protocol showed a slightly higher specificity to enrich Met-peptides as compared to the bromoacetyl-based method (96.9% vs. 87.5%), although the latter method could enrich a bit more Met-peptides (12452 vs. 14281) (Fig. S2a). In addition, we compared the ratios (Heavy/Light = Ctrl/PDT) of the 3964 reduced Met-peptides simultaneously quantified before and after enrichment by the two protocols. The results showed good correlations among each other (Spearman’s rho = 0.625–0.751) (Fig. S2b), implying that the levels of most reduced Met-peptides did not alter significantly by both enrichment protocols. Taken together, the results indicate that the efficiency of the iodoacetyl-PEG2-biotin-based protocol toward Met-peptide enrichment from complex biological samples is comparable to that of the bromoacetyl-based method.

### Met oxidation profiling in A431 cells treated with different Photofrin-PDT regimens

**Figure 2:**
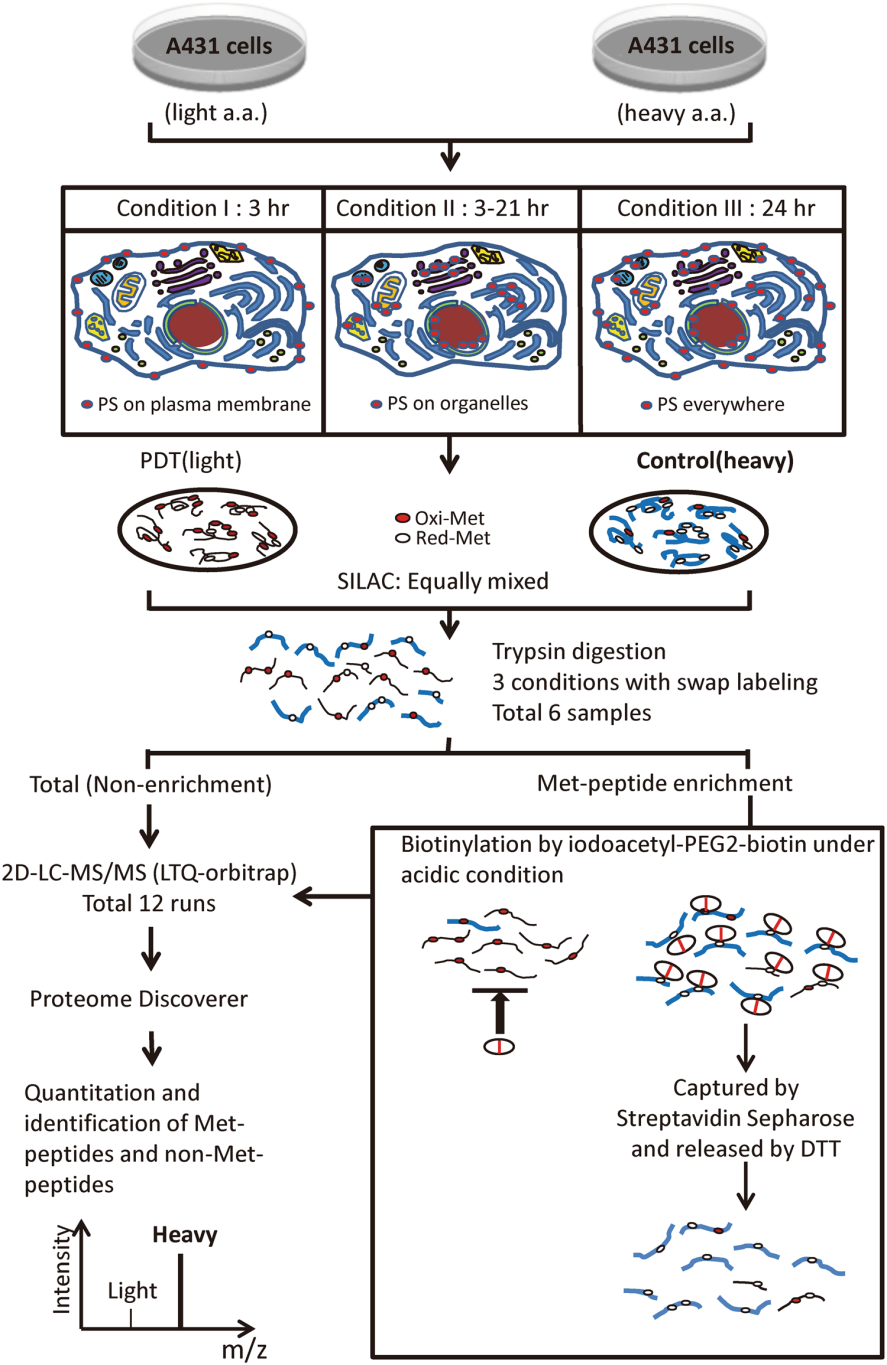
Workflow for Met oxidation profiling in A431 cells treated with different Photofrin-PDT regimens. SILAC-labeled A431 cells were preloaded with Photofrin (as photosensitizer, PS) under three different incubation conditions (conditions I-III), and treated with or without laser irradiation to generate paired control and PDT cell groups, respectively. Equal amount of protein extracts from the control and PDT groups were mixed at a 1:1 ratio and digested with trypsin. Each digested sample was divided into two parts; one part was directly analyzed by 2D-LC-MS/MS (LTQ-Orbitrap), while the other was subjected to iodoacetyl-PEG2-biotin-mediated Met-peptide enrichment, followed by the same 2D-LC-MS/MS analysis. A label-swap replication of the SILAC experiment was applied to each condition. The Protein Discoverer software (Thermo Scientific) was used to combine and analyze the MS data from all 12 runs.

Our previous studies showed that the distribution of Photofrin in living cells depends on the incubation protocol^15, 16^. Here, we used the workflow shown in Fig. 2 to systemically analyze the pattern of Met oxidation in living cells subjected to oxidative stress at distinct subcellular sites. We labeled A431 cells with light or heavy amino acids (the SILAC procedure) and manipulated the Photofrin distribution using different incubation protocols, as previously described^15, 16^. In condition I, Photofrin was mainly distributed at the plasma membrane; in condition II, Photofrin was internalized and preferentially accumulated in the ER and Golgi apparatus; and in condition III, cells were incubated with Photofrin for 24 hours to allow ubiquitous distribution. Cells preloaded with Photofrin under these conditions were left untreated (control group with SILAC labeled heavy) or subjected to laser irradiation (PDT group with SILAC labeled light), and proteins extracted from the paired cell groups were equally mixed and trypsin-digested. The samples were directly analyzed by 2D-LC-MS/MS (non-enriched sample) or subjected to Met-peptide enrichment followed by 2D-LC-MS/MS analysis (enriched sample). A label-swap replication of the SILAC experiment (Exp.1, PDT/Ctrl = Light/Heavy; Exp. 2, PDT/Ctrl = Heavy/Light) was performed for each condition, in an effort to improve the accuracy of our quantitation. Finally, the MS data obtained from the 12 LC-MS/MS runs were processed for protein and peptide identifications/quantifications (see Supplemental Materials and Methods for details).

### Identification of Met-peptides in the non-enriched and enriched samples

**Figure 3:**
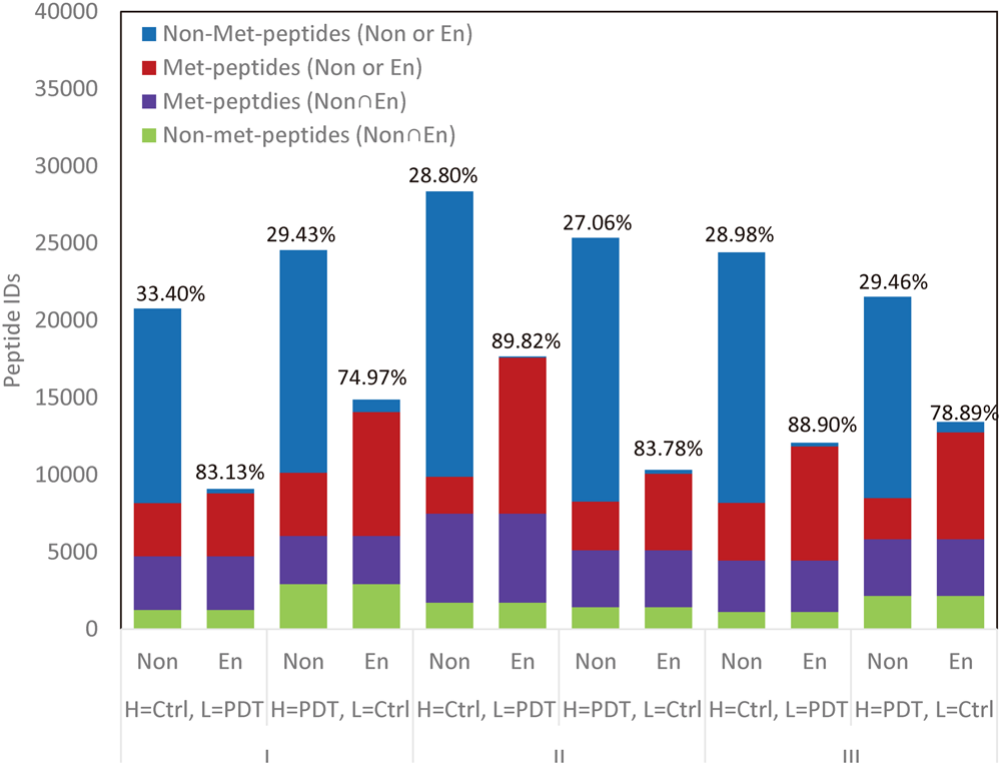
The efficiency of Met-peptide enrichment from A431 cells under the three different PDT conditions. The numbers of Met- and non-Met-peptides detected in the non-enriched and enriched samples were calculated for each condition. The three conditions (with swapping experiments) were analyzed separately. The red bars indicate the Met-peptides specifically identified in either non-enriched or enriched samples, while the purple bars indicate Met-peptides that were commonly identified in both, and the blue and green bars represent non-Met-peptides. The number above each column denotes the Met-peptides as a percentage of the total identified peptides.

In each of the six non-enriched samples, we identified more than 24,000 peptides with an average Met-peptide percentage of ~30% (7,099 Met-peptides per sample, including 1,641 oxidized and 5,458 reduced Met-peptides). In each of the six enriched samples, we identified ~12,900 peptides with an average Met-peptide percentage of 83% (10,766 Met-peptides per sample, including 5,642 oxidized and 5,124 reduced Met-peptides) (Fig. 3 and Table S1). Only 3,844 Met-peptides were common to the non-enriched and enriched samples, indicating that iodoacetyl-PEG2-biotin-based enrichment can greatly increase the number of Met-peptides identified (6,922 Met-peptides observed only in enriched samples on average) (Table S1). The total number of Met-peptides identified in each experiment increased from 7,099 to 10,766 on average after enrichment, representing an increase of 1.5 times. Our analysis of all 12 samples yielded 63,951 identified peptides, including 34,362 non-Met-peptides and 29,589 Met-peptides. Notably, 48% of the Met-peptides (14,179) were identified only after enrichment, demonstrating that our newly developed iodoacetyl-PEG2-biotin-based enrichment method efficiently enriches Met-peptides from complex biological samples. To evaluate whether the oxidative status changed during the enrichment procedure, we analyzed the correlation of the Met-peptide PDT/Ctrl ratios before and after enrichment. As shown in Fig. S3, these ratios were very well correlated in the six datasets (0.741~0.844), indicating that the ratios remained the same and the protocol should not alter the quantitative results.

### Quantitation of Met-peptide oxidation in A431 cells under conditions I, II and III

To confidently characterize the oxidized Met-peptides in Photofrin-PDT treated A431 cells, we performed two SILAC label-swap replicates for each condition, and selected Met-peptides whose changes in oxidation levels were both beyond the mean ±2 SD. Under the three PDT conditions, we identified a total of 431 Met-peptides (156, 29 and 322 for conditions I, II, and III, respectively) corresponding to 302 proteins that showed significantly altered Met oxidation (Table S2). Although Met-peptide oxidation in biological samples can be measured directly, some spontaneous oxidation of Met-peptides (~5–10%) can occur during sample preparation^39^. This could lead to underestimation of the change in Met-peptide oxidation (see Table S3 for a detailed illustration). In contrast, the ratios of reduced Met-peptide are not affected by spontaneous oxidation during sample preparation. Thus, this is a useful alternative way to monitor Met-peptide oxidation. More importantly, quantitative assessment of the changes in the oxidized and reduced forms of the same Met-peptide upon specific oxidative challenge enabled us to estimate the oxidized proportion of the target Met-peptide before and after treatment (see Table S4 for equation).

From non-enriched samples, we selected Met-peptide pairs with both oxidized and reduced forms that showed significant changes upon PDT treatment (beyond the mean ± 2 SD in both swapping experiments, see Table S2). This yielded 71 pairs from condition I, 135 pairs from condition III, and only eight pairs from condition II. As shown in Table S5 and Fig. S4, about 87% (62/71) of the Met-peptide pairs from condition I and 88% (119/135) of those from condition III showed good reciprocal relationships between the increase of oxidized Met-peptides and the decrease of reduced Met-peptides. Furthermore, 35% (25/71) of the Met-peptides from condition I and 40% (54/135) from condition III displayed oxidation proportions greater than 20% after PDT treatment. The representative MS spectra of selected Met-peptides observed with both oxidized and reduced forms are shown in Fig. S4c. Notably, PDT treatment had little effect on the levels of their parent proteins (Table S5). Thus, the PDT-induced alterations of the identified Met-peptides reflected post-translational changes, not alterations in protein expression.

**Figure 4:**
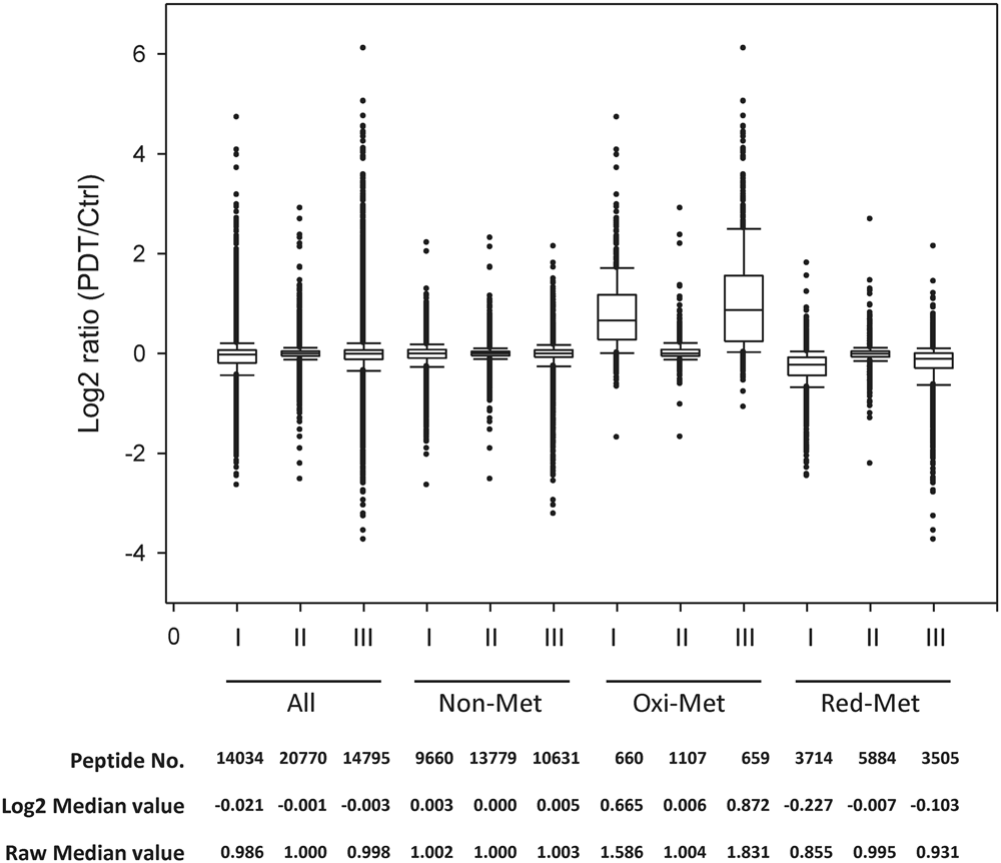
The log2 (PDT/Ctrl) ratio distributions of all identified peptides, non-Met-peptides, oxidized Met-peptides, and reduced Met-peptides under the three conditions. The box represents the 25 to 75% distribution, and the line in the box shows the median ratio. The upper and lower10% distributions are marked at the top and bottom of the box, respectively. The peptide numbers and the ratios of the medians are shown at the bottom.

Next, we analyzed the global distributions of the PDT/Ctrl ratios for all of the quantified peptides and proteins (Fig. S5), and further analyzed these ratios for all of the peptides, non-Met-peptides, oxidized Met-peptides, and reduced Met-peptides detected under the three PDT conditions (Fig. 4). There was no significant difference in the median PDT/Ctrl ratios of all peptides or the non-Met peptides. In contrast, the ratios of the oxidized Met-peptides were significantly increased under conditions I (median, 1.586) and III (median, 1.831), whereas those of the reduced Met-peptides were somewhat slightly decreased under the same conditions (median, 0.855 and 0.931, respectively). Under condition II, which would be expected to trigger only mild oxidative stress^24^, PDT triggered little change in the ratios of the oxidized and reduced Met-peptides (median, 1.004 and 0.995, respectively). Collectively, these results indicate that the systematic analysis of oxidized and reduced Met-peptides can be used to estimate the proportion of Met-peptide oxidation and the cellular oxidative status.

### Subcellular distribution of Met-oxidized proteins under different Photofrin-PDT conditions

**Figure 5:**
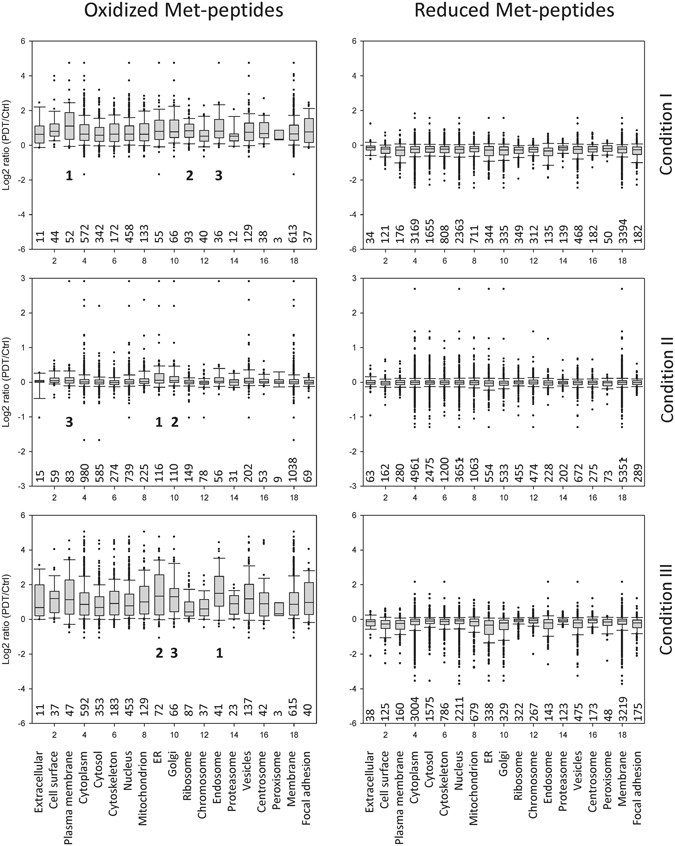
The redox status of cellular organelles in A431 cells subjected to the different Photofrin-PDT regimens. The proteins corresponding to the quantified Met-peptides (oxidized or reduced) were assigned to their known subcellular locations using GO analysis, and the log2 (PDT/Ctrl) ratio distributions of all oxidized (left panels) and reduced (right panels) Met-peptides were analyzed for each cellular site/organelle under each PDT condition. The box representations are the same as in Fig. 4. The numbers of oxidized (or reduced) Met-peptides grouped and analyzed in each cellular site/organelle are shown below each box. The top three most severely oxidized cellular structures in each PDT condition are indicated by 1, 2 and 3 in left panels.

To evaluate the Photofrin-PDT-mediated Met oxidation of proteins at the subcellular level, we matched the quantified oxidized and reduced Met-peptides with their parent proteins and assigned these proteins to their known subcellular locations by gene ontology (GO) analysis^40^. We then grouped the quantified Met-peptides by their related cellular structures (organelles), and quantitatively analyzed the Met oxidation of proteins according to the median ratios and 25^th^ to 75^th^ percentiles in the different GO categories (Fig. 5). Regarding the oxidized Met-peptides, most of the cellular structures exhibited much higher oxidative stress under conditions I and III compared to condition II as expected. The top three most severely oxidized cellular structures were (in order): under condition I, the plasma membrane (1.112), ribosome (0.837), and endosome (0.821); under condition III, the endosome (1.498), ER (1.333) and Golgi (1.299); and under condition II, the ER (0.055), Golgi (0.047), and plasma membrane (0.046). Regarding the reduced Met-peptides, their median log2 ratios (PDT/Ctrl) at the various cellular sites were all lower under conditions I and III versus condition II (Fig. 5). Under condition II, only proteins distributed in the ER, Golgi, or endosome showed slightly decreased levels of reduced Met-peptides (ER, −0.03; Golgi, −0.02; and plasma membrane, −0.02), which is consistent with the slight increases of the oxidized Met-peptides seen under this condition (ER, 0.055; Golgi, 0.047; and plasma membrane, 0.046) (Fig. 5, middle panels).This clearly indicates the feasibility of using our quantitative proteomics platform to profile protein Met-oxidation events at different subcellular sites. Our data also demonstrate for the first time that the Met oxidation of proteins correlates well with the subcellular distribution of Photofrin in PDT-treated cells.

### Consensus sequence of the oxidized Met-peptides

**Figure 6:**
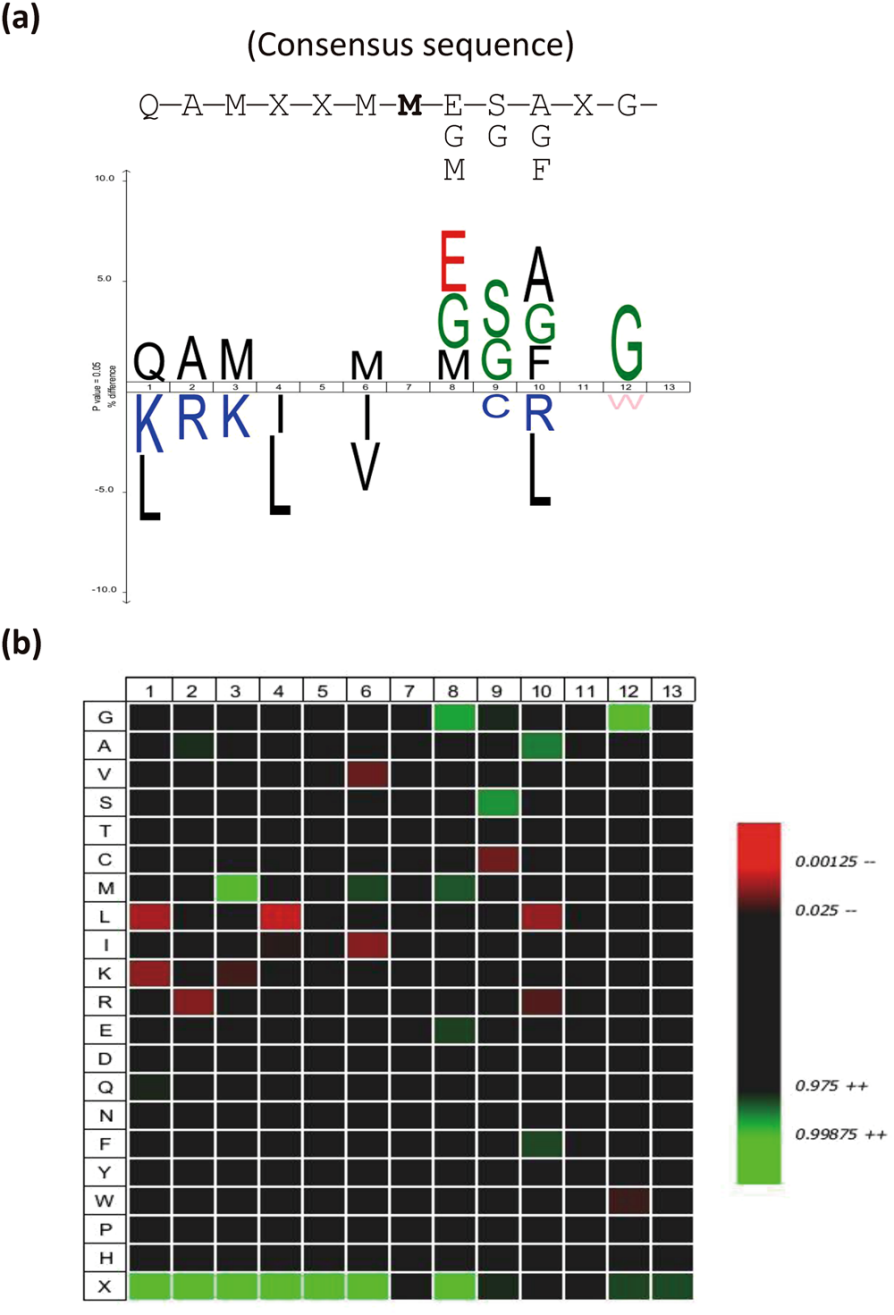
The consensus sequence of the oxidized Met-peptides, as analyzed using the iceLogo software. The frequencies of each amino acid at positions surrounding the oxidized Met residues of the 431 oxidized Met-peptides from three PDT conditions were analyzed with the iceLogo software, using a reference database containing all of the non-oxidized Met-peptides identified in our experiments. (**a**) The high- and low-frequency amino acid residues are shown at upper and lower part of the iceLogo, respectively. The oxidized Met residue is set at position 7. (**b**) A heat map generated from the same analysis. In both panels, only significantly overrepresented amino acid residues (*P* < 0.05) are shown.

To search for a consensus sequence for the Photofrin-PDT-induced Met oxidation of proteins, we calculated the frequency of various amino acid residues around the oxidized Met residues in the 431 Met-peptides that showed significant oxidization under Photofrin-PDT (Table S2). For this analysis, we applied the iceLogo software^33, 41^, using the non-oxidized Met-peptides as a reference set. This analysis revealed that “QAMXXM**M-**E/G/M-S/G-A/G/F-XG” (the bold M indicates the oxidized Met residue) is the consensus sequence for the oxidized Met-peptides (Fig. 6). This sequence motif shows three unique features: (1) Several Gly residues are located near the severely oxidized Met residue. Since Gly can enable a stereo turn in the secondary structure, this might suggest that a beta turn surrounds the oxidized Met-residue and potentially presents it on the protein surface. (2) The hydrophobic amino acid residues, Leu and Val, were the least likely to appear at the N-terminus of this sequence surrounding an oxidized Met residue, suggesting that the targeted Met-peptides have a hydrophilic character. The oxidation of surface Met residues will further increase the hydrophilicity of the protein surface^42^. (3) Two to three additional Met residues are located near the oxidized Met residue, potentially offering an antioxidant effect and acts as an oxidant scavenger for proteins to respond to oxidative stress^43^.

### Identification of potential Photofrin-binding proteins

**Figure 7:**
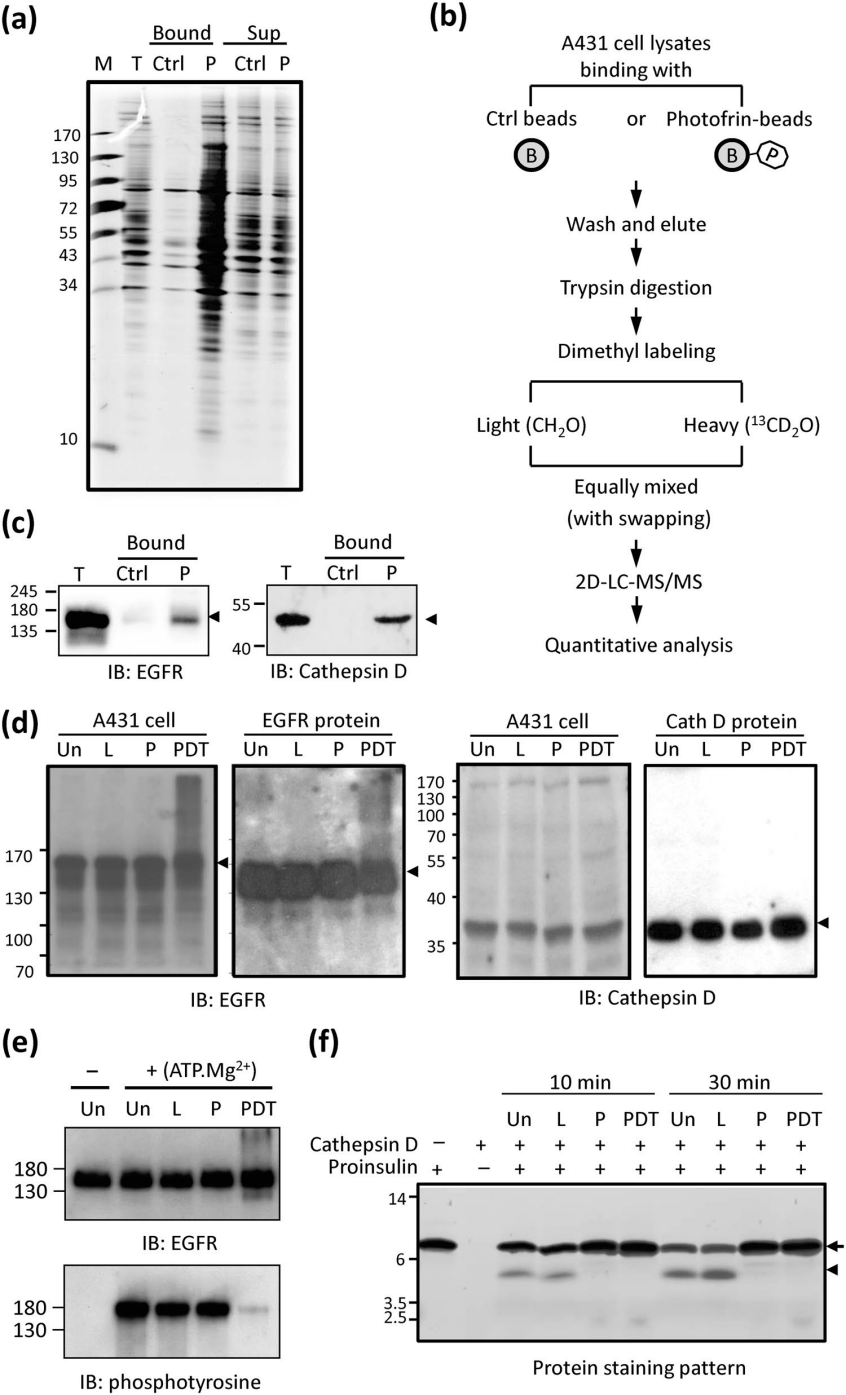
Identification and verification of Photofrin-interacting proteins. (**a**) SDS-PAGE analysis of potential Photofrin-interacting proteins. A431 cell lysates (1 mg of protein) were incubated with Photofrin-coupled beads (P) or control beads (Ctrl), and washed with 1 M NaCl and PBS. The bead-bound proteins (Bound) and the unbound supernatants (Sup) were subjected to SDS-PAGE followed by silver staining. T, total cell lysates. (**b**) The proteomics workflow used to identify potential Photofrin-interacting proteins. See the Materials and Methods for details. (**c**) Samples were processed as described in (**a**), and the bead-bound proteins (Bound) were subjected to SDS-PAGE followed by Western blot analysis with anti-EGFR or anti-cathepsin D antibodies. T, total cell lysates. (**d**) A431 cells or recombinant EGFR (or cathepsin D) proteins were left untreated (Un) or treated with laser irradiation (L), Photofrin (P), or both (PDT), and the cell lysates or reaction products were subjected to Western blot analysis with anti-EGFR (left panel) or anti-cathepsin D (right panel) antibodies. (**e**) Recombinant EGFR was treated as described in (**d**) and then incubated with (+) or without (−) kinase assay buffer containing ATP.Mg^2+^at 30 °C for 10 min. The reaction products were subjected to Western blot analysis with anti-EGFR or anti-phosphotyrosine antibodies. (**f**) Recombinant cathepsin D was treated as described in (**d**), and the untreated/treated cathepsin D proteins were incubated with a reaction mixture containing 0.5 μg proinsulin at 37 °C for 10 or 30 min. The reaction products were analyzed by SDS-PAGE followed by silver staining. The arrow and arrowhead indicate proinsulin and its processed product, respectively.

It is well known that PDT mainly attacks biomolecules near the photosensitizer, due to the very short lifetime of ROS in biological systems (<0.04 ms) and the small action radius of singlet oxygen (<0.02 mm)^44^. This, together with our previous finding that Photofrin binds procaspase-3 and mediates its PDT-triggered Met oxidation and inactivation^29^, prompted us to search for additional Photofrin-binding proteins in human cells. We hypothesized that such proteins might be more susceptible to oxidative damage during Photofrin-mediated PDT. We coupled Photofrin onto carboxyl-linked agarose beads and used them to pull down proteins from A431 cell lysates. As shown in Fig. 7a, numerous proteins were selectively pulled down by Photofrin-immobilized beads but not control beads. The use of a stable isotope dimethyl-labeling strategy combined with LC-MS/MS (Fig. 7b) enabled us to identify and quantify 803 proteins in both swapping experiments (Table S6). Comparison of these 803 proteins with the 302 proteins we had identified as being severely oxidized under Photofrin-PDT (Table S2) allowed us to identify 94 proteins as being potential Photofrin-binding proteins that are severely Met oxidized by PDT of A431 cells (Table S6).

### Effects of Photofrin-PDT on the enzyme activities of EGFR and cathepsin D

From among the 94 proteins identified above, we selected EGFR (a membrane protein with kinase activity) and cathepsin D (a lysosomal protein with protease activity) for further validation. As expected, Western blot analysis confirmed that Photofrin-coupled beads could pull down both EGFR and cathepsin D from A431 cell lysates (Fig. 7c). Interestingly, Photofrin-PDT caused the formation of some unusual high-molecular-weight products of EGFR, but not cathepsin D, both in A431 cells and when we used purified recombinant proteins (Fig. 7d). This suggests that protein-oxidation-mediated cross-linking of EGFR might occur during Photofrin-PDT. Assessment of autophosphorylation on tyrosine residues revealed that EGFR tyrosine kinase activity was dramatically reduced by Photofrin-PDT (Fig. 7e). Meanwhile, the cathepsin D-mediated proteolysis of its physiological substrate, proinsulin^45^, was almost completely blocked by Photofrin, regardless of PDT (Fig. 7f). Thus, our results demonstrate that the enzyme activities of EGFR and cathepsin D can be significantly blocked by Photofrin-PDT or Photofrin alone.

## Discussion

Recent progress in cellular oxidative stress research has come from the development of new fluorescent probes and reagents, as well as the improvement of analytical methodologies, including mass spectrometry^46^. Most of the studies on how the redox status can alter protein functions have focused on Cys oxidation. However, Met oxidation has gained recent attention, as it has been shown to play important roles in mediating conformational changes and regulating protein functions, such as by modulating actin assembly^47^, altering calcium signals^26^, and preventing phosphorylation^48, 49^. Several studies have sought to systematically identify oxidized Met residues in proteins under oxidative stress using quantitative redox proteomics approaches. Various separation strategies and/or enrichment protocols have been used to reduce sample complexity and enhance the identification of Met-peptides in biological samples. Previously, the COFRADIC procedure developed by Gevaert *et al*.^32, 33^ appeared to the most successful in identifying and quantifying Met oxidation on a proteome-wide scale. Here, we used the previously reported chemistry^34, 38^ and a commercially available iodoacetyl-PEG2-biotin reagent^37^ to develop a protocol that selectively captures reduced Met-peptides in biological samples. Starting from 200 μg cell lysates, this simple protocol enabled us to identify >10,000 Met-peptides on average, with less than 17% non-Met-peptide contamination (Fig. 3 and Table S1). When combined with the SILAC technique, the new protocol allows a more comprehensive assessment of the quantitative changes seen among reduced Met-peptides. Compared to the results obtained from non-enriched samples, the enrichment protocol enabled the detection and quantification of an additional 2,231 reduced Met-peptides, yielding the identification of another 258 peptides that were significantly altered (beyond mean ± 2 SD) by Photofrin-PDT, as well as many other reduced Met-peptides that were not altered by this treatment (Table S7). The quantification of additional reduced Met-peptides increased the pool of peptides for which we were able to estimate the oxidation proportions before and after PDT.

In the quantitation results obtained from SILAC, the peptide ratios represent fold-changes, not the proportion of oxidation. The Met oxidation percentage can only be estimated by the calculation as described in Table S4. As an example of the difference between the results of fold-changes to oxidation percentages, as shown in Table S5, the PDT/Ctrl ratio of the oxidized form of the peptide, NLLHVTDTGVGMTR (derived from the protein, endoplasmin) was 4.12, which would appear to be significant; however, the oxidation percentage increased only from 2.09 to 8.64 after PDT. Compare this to the peptide, NSLESYAFNMK (derived from Heat shock cognate 71 kDa protein), for which the PDT/Ctrl ratio of the oxidized form was 4.24, and the oxidation percentage increased from 15.18 to 55.15 after PDT. These two oxidized Met-peptides had similar PDT/Ctrl ratios, but their oxidation percentages were quite different. Thus, it is critical to estimate the proportion of oxidized proteins if we hope to begin understanding how severely the protein function might be altered.

It has long been recognized that protein oxidation critically mediates the therapeutic effects of Photofrin-PDT on tumor cells. However, little is known about the global picture of protein oxidation in Photofrin-PDT-treated tumor cells. Here, we show for the first time that the majority of the severely oxidized proteins are located at the plasma membrane, ER, Golgi, and endosome (Fig. 5); the top three most severely oxidized cellular structures are as below: the plasma membrane, ribosome, and endosome (under condition I); the ER, Golgi, and plasma membrane (under condition II); and the endosome, ER and Golgi (under condition III). These observations appear to follow the distribution properties for Photofrin in A431 cells that we found before: it is initially localized at the plasma membrane, but prolonged incubation allows it to enter the ER/Golgi^15^. It has been well documented that Photofrin (and its derivatives) can bind and partition into lipid bilayer membranes^50–52^, and our data showed that the proteins which reside in membrane-bound cellular structures (including plasma membrane, ER, Golgi, endosome, ribosome, vesicles, etc.) are among the most severely oxidized cellular proteins in all three PDT conditions (Fig. 5). Additionally, several proteins critical for endomembrane traffic pathways were found to be severely oxidized by Photofrin-PDT, such as clathrin heavy chain 1, coatomer subunit epsilon and zeta-1, dynamin-2, cation-dependent mannose-6-phosphate receptor, vesicle-trafficking protein SEC22b, protein transport protein Sec61 subunit beta, vacuolar protein sorting-associated protein 52 homolog, etc. (Table S5). Taken together, these observations suggest that when the lipophilic photosensitizer Photofrin binds and partitions into the cell membrane, it can be quickly internalized, probably through the endomembrane system as a major route, into the cells. The findings that significant increase of Met oxidation of proteins residing in endosome, ER, Golgi and ribosome could also be observed in PDT condition I (Fig. 5, upper panel) suggest that Photofrin may have already reached these membrane-bound cellular structures within 3 h incubation. After removal of Photofrin-containing medium and incubation with Photofrin-free medium for an additional 21 h (i.e. the PDT condition II), the cellular content of Photofrin dropped significantly, and the ER/Golgi retained the majority of residual Photofrin under this circumstance^15^, which is consistent with the observation that ER and Golgi represent the top two most severely oxidized cellular structures in condition II (Fig. 5, middle panel). In condition III, cells were incubated with Photofrin for 24 h to allow ubiquitous distribution, and this explains the high levels of Met oxidation of proteins in most cellular structures (Fig. 5, lower panel). In addition, it is noted that numerous cytosolic and cytoskeleton proteins could also be oxidized by Photofrin-PDT under conditions I and III. Direct binding to Photofrin diffused into the cytoplasm (such as calmodulin, S100 family protein A14 and cytoskeleton-associated protein 4, see Table S6) or locating in close vicinity of the Photofrin-containing, membrane-bound cellular structures may account for their oxidation during PDT.

Our findings are consistent with previous studies showing that PDT with different photosensitizers caused the carbonylation of various ER proteins, including PDI, calreticulin, GRP78, and heat shock protein 71^3, 53, 54^. Notably, these proteins were also identified herein as severely Met-oxidized proteins. Taken together, these observations imply that photosensitizers may locate at different cellular compartments and induce ROS to attack nearby proteins in numerous ways, such as by thiol oxidation, carbonylation, and methionine oxidation. A more comprehensive proteomics study by Tsaytler *et al*.^55^ used selective biotinylation, affinity purification and MS analysis to identify 314 carbonylated or Cys-oxidized proteins in A431 tumor cells treated with phthalocyanine (Pc4)-mediated PDT. Of them, 66 were also found as severely Met-oxidized proteins in our current study, including GRP78, annexin A1, EGFR, heat shock proteins, etc. (see Table S8). Thus, these 66 proteins appear to be highly sensitive to PDT, exhibiting carbonylation, Cys oxidation, and Met oxidation under different photosensitizers. We further compared the cellular protein targets oxidized under our conditions with those previously reported under other oxidative stresses, including H_2_O_2_, cadmium, or the silencing of methionine-sulfoxide reductase (Msr)^33, 56–58^. There are 98 proteins sensitive to other oxidative stresses; of them, 53 were also identified in this study, including heat shock cognate 71 kDa protein, cathepsin D, protein disulfide-isomerase, myosin-9, moesin, elongation factor 2, alpha-actinin-1, T-complex protein 1 subunit alpha, etc. (see Table S8). Since Cys and Met oxidation are reversible reactions^59^, these 53 proteins may act as general cellular antioxidants in moderating the effects of mild ROS stimulation. Our comparative analysis also revealed 210 proteins whose oxidations were detected only under Photofrin-PDT, and half of them (102 of 210) are ER, Golgi, or mitochondrial proteins (see Table S8).

In the present study, we detected 302 severely oxidized proteins represented by 431 Met-peptides that contained a total of 501 Met residues (Table S2). We analyzed the sequences of these 431 Met-peptides using the iceLogo software^33, 41^, and found that “QAMXXM**M-**E/G/M-S/G-A/G/F-XG” was the peptide sequence motif that was most frequently oxidized by Photofrin-mediated PDT (Fig. 6). Of the isolated Met-peptides, 21.8% (94 of 431) contained more than two Met residues, and 17.2% (74 of 431) had a Gly residue next to the target Met residue. An increasing number of proteins with important biological functions have been shown to undergo oxidant-mediated Met oxidation (reviewed in ref. 60). Interestingly, many of them have two closely located target Met residues that are susceptible to protein-function-modulating oxidation, including CaMKII (STVAS^281^M^282^MHRQET), prion (KPKTN^109^MKH^112^MAGAA and GLGGY^129^MLGSA^134^MSRPII), actin (HQGV^44^MVG^47^MGQKDS), the sodium channel, Nav3.1 (LLFAL^1476^M^1477^MSLPAL), and caspase-3 (DNSYK^39^MDYPE^44^MGLCII)^29^. In this study, calmodulin showed severe oxidation (PDT/Ctrl Log2 ratio = 4.34) at Met144/145, which is located at the most C-terminal surface-exposed part of the protein. These two Met residues are reportedly important for the tertiary structure maintenance and functional regulation of this protein^26^. The coordination of neighboring Met residues in the 3D structure is not easy to assess. However, the identification and quantification of oxidized Met-peptides by MS plus 3D-structure analysis can provide important clues as to how spatially adjacent Met residues coordinate with each other to regulate protein functions.

We identified 94 proteins as potential Photofrin-binding proteins that could also undergo severe Met oxidation upon PDT of A431 cells (Table S6). We selected EGFR and cathepsin D for functional validation, and found that their enzyme activities were significantly decreased by PDT (Fig. 7). We detected eleven EGFR-derived Met-peptides (containing Met111, 176, 178, 268, 277, 318, 766, 881, 987, 1002 and 1007) whose peptide levels were significantly altered by PDT (Table S2). Of them, GN^111^MYYENSYALAVLSNYDANK and AcGADSYE^318^MEEDGVR (PDT/Ctrl Log2 ratios = 3.07 and 3.55, respectively) represented the most severely oxidized Met-peptides of EGFR. According to the known crystal structure of EGFR, both Met111 and Met318 are surface-exposed, while Met111 is located at the bottom of the EGF-binding pocket. Oxidation of Met111 might therefore interfere with the interaction between EGFR and EGF. Furthermore, Met766 is located near activation loop during dimerization, and it is possible that Photofrin-PDT-triggered Met766 oxidation may interfere EGFR intracellular domain dimerization and inter-molecule phosphorylation^61^. Regarding cathepsin D, PDT most severely oxidized Met246, which is close to the active site Asp97/295 identified in the resolved 3D structure of cathepsin D^62^. Thus, further detailed investigations may be warranted to elucidate how Met oxidation can regulate the activities of EGFR and cathepsin D.

Environmental or drug-induced oxidative stress can cause Met oxidation of cellular proteins that participate in diverse biological processes. However, the global and quantitative analysis of protein Met oxidation remains challenged by technical limitations. This study describes a new method for Met-peptide enrichment and its combination with a SILAC-based quantitative proteomics approach, and our data provide a first insight into the oxidation status of distinct organelles in cells subjected to different Photofrin-PDT regimens, and suggest potential protein targets through which Photofrin-PDT may elicit its diverse effects on cancer cells.

## Materials and Methods

### Cell culture and SILAC labeling

Human epidermoid carcinoma A431 cells were cultured as previously described^1, 32^. For SILAC experiments, cells were maintained in SILAC medium comprising Dulbecco’s modified Eagle medium (DMEM; Invitrogen) supplemented with10% dialyzed FBS (Invitrogen). L-lysine and L-arginine(Sigma-Aldrich) or [^13^C_6_]-L-lysine and [^13^C_6_]-L-arginine (ISOTEC; Sigma-Aldrich) were added at 0.1 g/L for light or heavy stable isotope labeling, respectively, and cells were cultured for at least 10 doubling times to achieve 95% incorporation^63, 64^.

### Photofrin-mediated PDT and sample preparation

A431 cells were loaded with Photofrin (Pinnacle Biologics)in the dark at different subcellular sites using the previously described protocols^15^. For condition I, cells were incubated with 28μg/ml Photofrin (2.5 mg/ml stock solution in 5% dextrose) in culture medium for 3 h in the dark. For condition II, cells were incubated with 28 μg/ml Photofrin for 3 h, washed with fresh medium, and incubated with Photofrin-free medium for an additional 21 h. For condition III, cells were incubated with 28 μg/ml Photofrin for 24 h. After Photofrin loading, the cells were washed once with fresh medium and irradiated with a 632.8 nm He–Ne laser (Coherent) at a fluence rate of 15 mW to yield a total energy of 10 J/cm^2^. Cells treated under each condition and untreated paired control cells were washed with cold phosphate-buffered saline (PBS) and lysed in 0.1% SDS. The cell lysates were vortexed at room temperature for 15 min, sonicated in an ice bath, and centrifuged at 10,000× *rpm* for 10 min to remove insoluble debris. The protein concentration of each lysate was determined using a BCA protein assay kit (Thermo Scientific).

### The sample preparation, LC-MS/MS analysis, peptide identification, protein identification, and data processing are described in the Supplemental Information

#### Enrichment of Met-peptides with iodoacetyl-PEG_2_-biotin

Three hundred micrograms of iodoacetyl-PEG_2_-biotin (Thermo Scientific) were dissolved in 6 μl dimethyl sulfoxide (DMSO; Sigma-Aldrich)/18 μl acetonitrile (ACN; J.T. Baker)/0.15 μl acetic acid (Sigma-Aldrich) and purified on Source 15 S resin (GE Life Sciences) to remove contaminants. A431 SILAC cell lysates (200 μg) were reduced, alkylated, digested, desalted, and vacuum dried. The dried peptide mixtures were mixed with pre-cleaned iodoacetyl-PEG_2_-biotin reagent (for model proteins, 200 pmole in 300 μg reagent; for cell lysates, 200 μg in 2 mg reagent). The ACN was removed with a speed vacuum, and the mixture was incubated at 50 °C for 6 h in the dark. After labeling, the excess iodoacetyl-PEG_2_-biotin reagent was removed using Source 15S resin, and the samples were eluted with 1 M sodium chloride/100 mM Tris pH 8.5. The eluted peptides were diluted 5-fold with water and shaken gently with 100 μl of streptavidin sepharose beads (GE Life Sciences) for 30 min at room temperature. After affinity capture, the sample was centrifuged at 1000 × *g* for 3 min, and the beads were washed three times with 0.2 M sodium chloride/20 mM Tris, pH 8.5. After the washing step, the beads were resuspended in 1 M Tris, pH 8.8, and 2 M DTT at 50 °C for 5 h to release the peptides. The released peptides were desalted using Source 15RPC resin, eluted by stepwise ACN (25, 50, 75% ACN/0.1% FA) and identified by LC-MS/MS.

#### Enrichment of Met-peptides with bromoacetyl-glass beads

Two hundred micrograms of cell lysates were digested by trypsin and reacted with bromoacetyl-glass beads under acetic condition as in the manufacturer’s instruction (PrepTide methionine selective matrix, Pierce). After capture, the glass beads were washed and released by 1 M ammonium bicarbonate and 14% beta-mercaptoethanol. Then the samples were cleaned up by the reverse phase silica in the kit as described and analyzed by LC-MS/MS.

#### Organelle oxidation index

The identified/quantified peptides were classified according to the cellular distributions of their parent proteins, as assessed by GO using the Proteome Discoverer software (Thermo Scientific). The ratios of oxidized and reduced Met-peptides classified into each organelle/subcellular site were analyzed with SigmaPlot (version 10.0; Systat Software Inc.).

#### Immunoblotting

Immunoblotting was carried out essentially as described previously^15^. Briefly, proteins were resolved by SDS-PAGE, transferred to a PVDF membrane, and probed with monoclonal anti-EGFR (Santa Cruz Biotechnology), anti-cathepsin D, (Santa Cruz Biotechnology), or anti-phosphotyrosine (Millipore).

#### Coupling of Photofrin to carboxyl-linked agarose, and affinity purification of Photofrin-binding proteins

Photofrin (100 μg) was mixed and incubated with 2.5 μl EDC (1-ethyl-3-[3-dimethylamino-propyl] carbodiimide hydrochloride; Thermo Scientific) 120 mg/ml in 0.1 M MES buffer, pH 4.7, 0.45 mg NHS (*N-*hydroxy-succinimide; Thermo Scientific) and 10 μl CarboxyLink^TM^ coupling gel (Thermo Scientific) at room temperature for 3 h. The beads were then washed six times with PBS and used as Photofrin-coupled beads. Photofrin-coupled or control beads were incubated with 1 mg A431 cell lysate at 4 °C for 3 h, supernatants were collected, and the beads were washed three times with 1 M NaCl and three times with PBS. The washed beads were divided into two portions. The first was subjected to SDS-PAGE. The second was subjected to reduction/alkylation by DTT/IAA and eluted by 6 M guanidine hydrochloride (GndCl; Sigma-Aldrich). The eluted proteins were diluted 20-fold with 25 mM ammonium bicarbonate to a final concentration of 0.2 M GndCl, and then digested overnight with trypsin (enzyme: substrate = 1:50). The resulting tryptic peptides were desalted with C18 Z tips (Millipore) and subjected to dimethyl labeling for quantitative analysis (see below).

#### Dimethyl labeling

Dimethyl labeling was performed according to the previously described protocol^65^. Dimethyl-labeled peptides from control and Photofrin-coupled beads (heavy/light and swapping) were mixed and subjected to 2D-LC-MS/MS analysis. The acquired data were processed using the Proteome Discoverer software (Thermo Scientific).

#### Assay of EGFR autophosphorylation

Purified EGFR (50 ng) in 10 μl of buffer A (50 mM HEPES, pH 7.6, 150 mM NaCl, 0.05% Triton X-100, 1 mM DTT; Sigma-Aldrich) was treated with or without Photofrin-PDT (28 μg/ml Photofrin and irradiation with a 632.8 nm He–Ne laser to yield a total energy of 10 J/cm^2^). After treatment, 20 μl of assay buffer (50 mM HEPES, pH 7.6, 20 mM MgCl_2_, 3 mM MnCl_2_, 3 mM Na_3_VO_4_, 1 mM DTT, and 0.2 mM ATP) was added, and the autophosphorylation reaction mixture was incubated at 30 °C for 10 min. The reaction products were resolved by 7.5% SDS-PAGE, transferred to PVDF membranes, and probed with anti-phosphotyrosine (Millipore).

#### Cathepsin D activity assay

Recombinant human cathepsin D (250 ng; R&D Systems) was dissolved and activated in 25 μl assay buffer (0.1 M NaOAc, 0.2 M NaCl, pH 3.5) at 37 °C for 30 min^66^.The activated cathepsin D proteins were diluted 10 times in assay buffer and divided into two portions. The first was left untreated, while the second was treated with Photofrin (28 μg/ml)-PDT. The untreated/treated cathepsin D (10 ng cathepsin D in a volume of 10 μl) proteins were mixed with a 5 μl reaction mixture containing 0.5 μg proinsulin (R&D Systems), and then incubated at 37 °C for 10 or 30 min. The reaction was stopped by the addition of SDS-sample buffer, and the mixture was boiled for 5 min and analyzed by SDS-PAGE/silver staining.

#### Statistical analysis

The peptide ratios are transformed to log2 scale, and all statistical data are expressed as the mean ± SD. Peptides ratios were considered significantly changed when the values were beyond mean ± 2 SD. Correlation analysis between two ranked variables was performed using SPSS (IBM Corporation) to calculate Spearman’s rank correlation coefficient. The quantile plots were generated using SigmaPlot.

## Electronic supplementary material

Supplementary Information
