# Supplementary material for: Oxidation of protein-bound methionine in Photofrin-photodynamic therapy-treated human tumor cells explored by methionine-containing peptide enrichment and quantitative proteomics approach
Source: Sci Rep. 2017 May 2;7:1370. doi: 10.1038/s41598-017-01409-9 (PMC5431048; doi:10.1038/s41598-017-01409-9)
Supplement: Supplementary file 1 — Supplementary Information [file 41598_2017_1409_MOESM1_ESM.pdf]

Supplementary information

Oxidation of protein-bound methionine in Photofrin-photodynamic therapy-treated human tumor cells explored by methionine-containing peptide enrichment and quantitative proteomics approach

Ya-Ju Hsieh[1#], Kun-Yi Chien[1,2,3#], I-Fang Yang[2], I-Neng Lee[4], Chia-Chun Wu[2], Tung-Yung Huang[2] and Jau-Song Yu[1,2,3,5]*

[1]Molecular Medicine Research Center; [2] Graduate Institute of Biomedical Sciences;
[3]Department of Biochemistry and Molecular Biology, College of Medicine, Chang Gung University, Taoyuan, Taiwan
[4]Department of Medical Research, Chang Gung Memorial Hospital, Chia-Yi, Taiwan.
[5]Liver Research Center, Chang Gung Memorial Hospital, Linkou, Taiwan

Materials and Methods

Reduction, alkylation and sample preparation

Equally mixed SILAC labeled cell lysates were dissolved in 50 mM ammonium bicarbonate, reduced with 10 mM dithiothreitol (DTT; Sigma-Aldrich) at 56ºC for 30 min, alkylated with 30 mM iodoacetamide (IAA; Sigma-Aldrich) at room temperature for 30 min, and neutralized with another incubation in 10 mM DTT. The samples were then digested with trypsin (Promega) overnight at 37ºC (trypsin/protein ratio 1:50). The peptide samples were desalted with Source 15RPC resin (GE Life Sciences), aliquoted at 200 μg per tube, dried with a speed vacuum, and stored at -80ºC for subsequent experiments.

Preparation and quantitative analysis of a model Met-peptide

The model Met-peptide, TVMENFVAFVDK, was purchased from Sigma-Aldrich. The peptide was dimethylated by heavy ($^{13}CD_2O$) (ISOTEC; Sigma-Aldrich) or light (CH_2O) formaldehyde as previously described [1], and the resulting heavy peptide was further oxidized by 0.5% (w/v) H_2O_2 at 30ºC for 30 min. Both oxidized (H) and reduced (L) peptides were purified by HPLC, quantified by LC-UV, and equally mixed. The heavy and light peptides (250 pmol each) were then mixed together with the tryptic digests of three proteins (horse heart cytochrome c, chick egg white lysozyme, and bovine carbonic anhydrase; 250 pmol each; Sigma-Aldrich), and the mixture was subjected to Met-peptide enrichment using the iodoacetyl-PEG2-biotin-based protocol. The reduced and oxidized forms of this model Met-peptide before and after enrichment were then analyzed by LC-MS/MS.

On-line 2D LC system and LC-MS/MS analysis using LTQ-Orbitrap

A total of 20 μg non-enriched samples or Met-enriched peptides from 200 μg lysates were injected into a strong cation exchange (SCX) column (Luna SCX,5μm, 0.5×150 mm;

Phenomenex) and separated into 22 fractions using a continuous ammonium chloride gradient in the presence of 30% ACN and 0.1% FA. Each SCX fraction was online diluted with 0.1%FA/H_2O and trapped on an RP18 column (Source 15 RPC, 0.5×5 mm; GE Life Sciences) and separated using coupled BEH RP18 chromatography (1.7μm, 0.1×120 mm; Waters) with an ACN gradient in 0.1% FA performed on a Dionex UltiMate 3000 nanoLC system (Thermo Fisher). Peptides eluted from the 2D-LC were nanoelectrosprayed to the LTQ-Orbitrap mass spectrometer (Thermo Fisher). The m/z 445.120025 was used for lock mass calibration in the Orbitrap to improve mass accuracy. Full-scan MS spectra (m/z 430 - m/z 2000) were acquired in the Orbitrap at a resolution of 30,000 (FWHM). The top twelve ions in each spectrum, each having a signal intensity greater than 15,000, were sequentially isolated for MS/MS fragmentation in the linear ion trap using a collision-induced dissociation energy of 35%, Q activation at 0.25, an activation time of 30 ms, and an MS/MS isolation width of 2 Da. Each precursor ion m/z with error less than 30 ppm was selected and analyzed twice, and was then excluded during the following 1 min.

Peptide and protein identification and data analysis

For the cell lysate experiments, MS and MS/MS spectrum obtained from LTQ-Orbitrap were analyzed using the Proteome Discoverer software (version 1.4.1.14; Thermo Scientific). MS/MS spectra were searched against SwissProt 2015 human database (total 20,205 entries) and the decoy database combined with common contaminants. The Mascot search parameters were set as follows: carbamidomethylation (Cys) as the fixed modification; oxidation (Met), Gln->pyro-Glu (N-term), acetylation (protein N-term), and $^{13}C_6$-Lys and $^{13}C_6$-Arg as variable modifications; 10 ppm for MS tolerance; 0.7 Da for MS/MS tolerance; and 2 for missing cleavage. The SILAC label (K) (R) was set as the quantitation method. For peptide and protein identification, the false discovery rate was set to < 0.01. In addition, a posterior error probability

for each MS/MS spectrum below or equal to 0.1 was required. The identified proteins were required to fit the following criteria: more than two peptides were identified, and at least one of them was a unique peptide.

For peptide/protein quantification, since arginine can be converted to proline during the SILAC process [2], we used the following strategy: Firstly, we normalized the SILAC conversion efficiency by calculating the average ratio of peptides containing one proline divided by the ratio of non-proline peptides. The obtained conversion efficiencies (Arg→Pro) were as follows: condition I, 7.96%; condition I swap, 2.05%; condition II, 1.73%; condition II swap, 1.31%; condition III, 14.97%; and condition III swap, 14.8%. Secondly, we obtained protein ratios by the median ratio of their non-Met-peptides. Thirdly, we used global median normalization to normalize the protein ratio, in an effort to reduce the system error arising from sample preparation. Fourthly, we normalized the observed peptide ratios by their protein ratios to ensure that the differences in the peptide ratios reflected oxidative modification rather than any PDT-induced alteration in protein expression. Peptides without a protein ratio were excluded from this analysis. We also excluded oxidized Met-peptides detected from the enriched samples, which would represent byproducts generated by the step in which Met-peptides were released from the streptavidin beads by incubation at 50°C for 6 h [3]. Fifth and finally, we averaged the peptide ratios between swapping experiments. If the peptide ratios were only available from one experiment, the peptide was excluded; if they were available from both non-enriched and enriched samples, these ratios were averaged unless they were discordant between swapped experiments and differed by more than 2SD, at which point they were excluded.

Table S1. Identification of proteins and peptides in the 12 samples.

Table S2. List of 431 significantly changed Met-peptides (corresponding to 302 proteins) detected under the three PDT conditions.

Table S3. Theoretical calculation of how oxidation during sample preparation could affect the observed ratios of oxidized and reduced Met-peptides between PDT and control samples.

Table S4. Theoretical calculation of the percentage of Met oxidation of target Met peptides before and after PDT.

Table S5. The Met-peptide pairs that showed significant changes in oxidation status under all three PDT conditions.

Table S6. List of potential Photofrin-binding proteins identified by affinity purification coupled with quantitative proteomics approach.

Table S7. Quantitation of reduced Met-peptides in the 12 samples.

Table S8. A cross-study comparison of oxidative stress-sensitive proteins.

Figure S1. Quantitative analysis of capture efficiency of the iodoacetyl-PEG2-biotin-based protocol for oxidized and reduced forms of a model Met-peptide.

Figure S2. Comparisons between the two protocols used to enrich Met-peptides from lysates of PDT-treated A431 cells.

Figure S3. Spearman's correlation coefficient analysis of the ratios of reduced Met-peptides from non-enriched versus enriched samples.

Figure S4. Reciprocal relationship between the increase of oxidized Met-peptides and the decrease of reduced Met-peptides in A431 cells treated with Photofrin-PDT.

Figure S5. Global distribution of the protein/peptide ratio (Log2) determined by LC-MS/MS in the six non-enriched samples.

Supplemental References

[1] Hsu JL, Huang SY, Chow NH, Chen SH. Stable-isotope dimethyl labeling for quantitative proteomics. *Anal Chem* 75: 6843-52, 2003.

[2] Ong SE, Kratchmarova I, Mann M. Properties of 13C-substituted arginine in stable isotope labeling by amino acids in cell culture (SILAC). *J Proteome Res* 2: 173-81, 2003.

[3] Zang L, Carlage T, Murphy D, Frenkel R, Bryngelson P, Madsen M, Lyubarskaya Y. Residual metals cause variability in methionine oxidation measurements in protein pharmaceuticals using LC-UV/MS peptide mapping. *J Chromatogr B Analyt Technol Biomed Life Sci* 895-896: 71-6, 2012.

Table S1. Identification of proteins and peptides in the 12 samples

A label-swap replication of SILAC experiments (Exp.1, PDT/Ctrl = Light/Heavy; Exp. 2, PDT/Ctrl = Heavy/Light) was applied to each condition. For each sample, we analyzed peptides with (Enriched) or without (Non-enriched) Met-peptide enrichment. % Of Met-peptides represents the percentage of Met-peptides among the total identified peptides. The numbers shown in the intersecting regions of the blue and red circles denote peptides that were identified in both enriched and non-enriched samples.

Protein/peptide identification	Condition I				Condition II				Condition III			
	H=Ctrl, L=PDT		H=PDT, L=Ctrl		H=Ctrl, L=PDT		H=PDT, L=Ctrl		H=Ctrl, L=PDT		H=PDT, L=Ctrl	
	Non-enriched	Enriched	Non-enriched	Enriched	Non-enriched	Enriched	Non-enriched	Enriched	Non-enriched	Enriched	Non-enriched	Enriched
No. of proteins	3052	1556	3600	2684	3834	2889	3549	1885	3448	1911	3154	2145
No. of peptides	20752	9103	24546	14884	28363	17657	25342	10337	24398	12086	21521	13442
No. of Non-Met-peptides	13821	1536	17323	3725	20194	1798	18484	1677	17327	1341	15181	2837
No. of Met-peptides	6931	7567	7223	11159	8169	15859	6858	8660	7071	10745	6340	10605
% of Met-peptides	33.40	83.13	29.43	74.97	28.80	89.82	27.06	83.78	28.98	88.90	29.46	78.89
Non-Met-peptides	12562 1259 277		14393 2930 795		18463 1731 67		17051 1433 244		16202 1125 216		13006 2175 662	
Met-peptides	3452 3479 4088		4095 3128 8031		2399 5770 10089		3170 3688 4972		3733 3338 7407		2678 3662 6943	

Table S2. List of 431 significantly changed Met-peptides (corresponding to 302 proteins) detected under the three PDT conditions

						Non-enriched experiment [Log2 (PDT/Ctrl) ratio]			Enriched experiment [Log2 (PDT/Ctrl) ratio]			ID by other studies
Protein	Description	Location	Sequence	Modification	No. of Met residues	I	II	III	IE	IIE	IIE	
Q9NRX4	14 kDa phosphohistidine phosphatase	cytoplasm; cytosol	IHVYGYSMAYGPAQHAISTEK	M8(Oxidation)	1		-1.68 a					
P62258	14-3-3 protein epsilon	cytoplasm; cytoskeleton; cytosol; mitochondrion	DNLTLWTSDMQGDGEEQNK	M10(Oxidation)	1	1.33 a		1.94 a				#
			DNLTLWTSDMQGDGEEQNKEALQDVEDENQ	M10(Oxidation)	1			2.04 a				#
			MDDREDLVYQAK	N-Term(Acetyl); M1(Oxidation)	1	1.75 a						#
P31947	14-3-3 protein sigma	cytoplasm; extracellular; nucleus	SAYQEAMDISKK	M7(Oxidation)	1			1.40 a				
			TTFDEAMADLHTLSEDSYK	M7(Oxidation)	1			1.78 a				
Q15392	24-dehydrocholesterol reductase	cytoplasm; cytoskeleton; cytosol; endoplasmic reticulum; Golgi; membrane; nucleus	EEFWEMFDGSLYHK		1			-1.44 a			-1.42 a	
P43686	26S protease regulatory subunit 6B	cytoplasm; cytosol; mitochondrion; nucleus; organelle lumen; proteasome	MEEIGILVEK	N-Term(Acetyl); M1(Oxidation)	1			1.41 a				
P35998	26S protease regulatory subunit 7	cytoplasm; cytosol; mitochondrion; nucleus; organelle lumen; proteasome	IDPTVTMMQVEEKPDVTYSDVGGCK	M7(Oxidation); C24(Carbamidomethyl)	2			1.21 a				#
			IDPTVTMMQVEEKPDVTYSDVGGCK	M7(Oxidation); M8(Oxidation); C24(Carbamidomethyl)	2	2.06 a						#
			IDPTVTMMQVEEKPDVTYSDVGGCK	M8(Oxidation); C24(Carbamidomethyl)	2			1.37 a				#
P62195	26S protease regulatory subunit 8	cytoplasm; cytosol; nucleus; organelle lumen; proteasome	ALDGPEQMELEEGK	N-Term(Acetyl); M8(Oxidation)	1			1.48 a				
O00487	26S proteasome non-ATPase regulatory subunit 14	cytoplasm; cytosol; nucleus; organelle lumen; proteasome	AVEEEDKMTPEQLAIK	M8(Oxidation)	1			1.31 a				
Q13200	26S proteasome non-ATPase regulatory subunit 2	cytoplasm; cytosol; nucleus; organelle lumen; proteasome	FAADIISVLAMTMSGER	M11(Oxidation)	2			1.79 a				
P25398	40S ribosomal protein S12	cytoplasm; cytosol; ribosome	AEEGIAAGGVMDVNTALQEVLK	N-Term(Acetyl); M11(Oxidation)	1			1.69 a				
P62263	40S ribosomal protein S14	cytoplasm; cytosol; mitochondrion; nucleus; organelle lumen;	DESSPYAAMLAAQDVAQR	M9(Oxidation)	1			1.94 a				
P08708	40S ribosomal protein S17	cytoplasm; cytosol; ribosome	LLDFGSLSNLQVTQPTVGMNFK	M19(Oxidation)	1			1.92 a				
P15880	40S ribosomal protein S2	cytoplasm; cytosol; nucleus; organelle lumen; ribosome	LLMMAGIDDCYTSAR	M3(Oxidation); M4(Oxidation); C10(Carbamidomethyl)	2	1.92 a						
P62081	40S ribosomal protein S7	cytoplasm; cytoskeleton; cytosol; nucleus; organelle lumen;	IVKPNGEKPDEFESGISQALLELEMNSDLK	M25(Oxidation)	1	1.23 a						
P08195	4F2 cell-surface antigen heavy chain	cell surface; cytoplasm; membrane	EVELNELEPEKQPMNAASGAAMSLAGAEK		2	-1.48 a		-1.65 a			-1.28 a	#
			EVELNELEPEKQPMNAASGAAMSLAGAEK	M14(Oxidation); M22(Oxidation)	2	3.98 a						#
			QPMNAASGAAMSLAGAEK	N-Term(Gln->pyro-Glu)	2				-1.17 a		-1.79 a	#
P10809	60 kDa heat shock protein, mitochondrial	cell surface; cytoplasm; cytosol; endosome; extracellular; membrane; mitochondrion; nucleus; organelle lumen	ALMLQGVDLLADAVAVTMGPK	M3(Oxidation); M18(Oxidation)	2	1.64 a						
			IMQSSSEVGYDAMAGDFVNMVEK	M2(Oxidation)	3			1.50 a				
P26373	60S ribosomal protein L13	cytoplasm; cytosol; ribosome	LATQLTGPVMPVR	M10(Oxidation)	1	1.75 a		1.66 a				
P62829	60S ribosomal protein L23	cytoplasm; cytosol; nucleus; organelle lumen; ribosome	LPAAGVGDMVMATVK	M9(Oxidation); M11(Oxidation)	2	2.64 a						
P11021	78 kDa glucose-regulated protein	cell surface; cytoplasm; cytosol; endoplasmic reticulum; extracellular; membrane; nucleus;	TFAPEEISAMVLTK	M10(Oxidation)	1	1.57 a						#
O96019	Actin-like protein 6A	chromosome; membrane; nucleus; organelle lumen	DDGSTLMEIDGDKGK	M7(Oxidation)	1			1.35 a				
P07108	Acyl-CoA-binding protein	cytoplasm; mitochondrion	QATVGDINTERPGMLDFTGK	M14(Oxidation)	1			1.72 a				
Q01518	Adenylyl cyclase-associated protein 1	cytoplasm; cytoskeleton; cytosol; extracellular; membrane	ADMQNLVER	N-Term(Acetyl); M3(Oxidation)	1	2.27 a		2.82 a				#
P05141	ADP/ATP translocase 2	cytoplasm; cytoskeleton; membrane; mitochondrion; organelle lumen	MTDAAVSFAK	N-Term(Acetyl)	1		-0.87 a					
			MTDAAVSFAK	N-Term(Acetyl); M1(Oxidation)	1			2.89 a				
P12236	ADP/ATP translocase 3	cytoplasm; membrane;	MTEQAISFAK	N-Term(Acetyl)	1		-0.80 a					
			MTEQAISFAK	N-Term(Acetyl); M1(Oxidation)	1			3.32 a				
P84077	ADP-ribosylation factor 1	cytoplasm; cytosol; Golgi; membrane; nucleus	QDLPNAMNAAEITDKLGLHSLR		1						-1.41 a	
			QDLPNAMNAAEITDKLGLHSLR	N-Term(Gln->pyro-Glu)	1						-1.19 a	
P62330	ADP-ribosylation factor 6	cytoplasm; endosome; Golgi; membrane; nucleus	QDLPDAMKPHEIQEK		1						-1.64 a	
			QDLPDAMKPHEIQEK	N-Term(Gln->pyro-Glu)	1						-1.71 a	

P40616	ADP-ribosylation factor-like protein 1	cytoplasm; Golgi; membrane;	QDMEQAMTSSEMANSLGLPALK		3											-0.94	a	
			QDMEQAMTSSEMANSLGLPALK	N-Term(Gln->pyro-Glu)	3											-1.02	a	
Q9NVJ2	ADP-ribosylation factor-like protein 8B	cytoplasm; cytoskeleton; endosome; membrane; nucleus;	GVNAIVYMIDAADREK		1											2.15	a	
P55196	Afadin	cytoplasm; cytosol; membrane; nucleus; organelle lumen	ITLDAQDVLAHLVQMAFK		1	-1.54	a											
P12814	Alpha-actinin-1	cytoplasm; cytoskeleton; cytosol; extracellular; membrane; nucleus; organelle lumen	ETADTDTADQVMASFK	M12(Oxidation)	1	2.44	a			2.89	a							#
			GISQEQMNEFR	M7(Oxidation)	1	1.89	a			2.26	a							#
			LSNRPAFMPSEGR	M8(Oxidation)	1					2.32	a							#
			MDHYDSQQTNDYMQPEEDWDRDLLLDPAWEK	N-Term(Acetyl); M1(Oxidation); M13(Oxidation)	2	1.72	a											#
			MVSDINNAWGCLEQVEK	M1(Oxidation); C11(Carbamidomethyl)	1					2.20	a							#
			VLAVNQENEQLMEDYEK	M12(Oxidation)	1	1.56	a											#
O43707	Alpha-actinin-4	cytoplasm; cytoskeleton; extracellular; nucleus; organelle lumen	GISQEQMQEFR	M7(Oxidation)	1	1.89	a			2.05	a							#
P61163	Alpha-centractin	cytoplasm; cytoskeleton; cytosol	MESYDVIANQPVVIDNGSGVIK	N-Term(Acetyl); M1(Oxidation)	1	1.35	a											
P06733	Alpha-enolase	cytoplasm; cytosol; membrane; nucleus	LMIEMDGTENK	M5(Oxidation)	2	1.16	a			1.97	a							#
P04083	Annexin A1	cytoplasm; cytoskeleton; extracellular; membrane; mitochondrion; nucleus	AMVSEFLK	N-Term(Acetyl); M2(Oxidation)	1	2.35	a											#
			MYGISLCQAILDETK	M1(Oxidation); C7(Carbamidomethyl)	1	2.00	a			2.72	a							#
			MYGISLCQAILDETKGDYEK	M1(Oxidation); C7(Carbamidomethyl)	1					2.62	a							#
P08758	Annexin A5	cell surface; cytoplasm; membrane	NFATSLYSMIK	M9(Oxidation)	1					2.32	a							
P00966	Argininosuccinate synthase	cytoplasm; cytosol; endoplasmic reticulum; membrane; mitochondrion; nucleus; vacuole	MPEFYNR	M1(Oxidation)	1	1.65	a											
Q12797	Aspartyl/asparaginyl beta-hydroxylase	cytoplasm; endoplasmic reticulum; membrane	QQFLGHMR	N-Term(Gln->pyro-Glu)	1											-2.03	a	
P06576	ATP synthase subunit beta, mitochondrial	cell surface; cytoplasm; membrane; mitochondrion; organelle lumen	EGNDLYHEMIESGVINLK		1					-1.29	a					-1.25	a	#
			FLSQPFQVAEVFTGHMGK		1											-1.04	a	#
			TREGNDLYHEMIESGVINLK		1					-1.40	a					-1.20	a	#
			TVLIMELINNVAK	M5(Oxidation)	1					2.44	a							#
P50895	Basal cell adhesion molecule	cell surface; membrane	LASAEMQGSELQVTMHDTR		2											-1.25	a	
P35613	Basigin	cytoplasm; Golgi; membrane; mitochondrion	SELHIENLNMEADPGQYR		1	-2.51	a			-2.96	a	-2.42	a			-2.55	a	
			SSEHINEGETAMLVCK	C15(Carbamidomethyl)	1	-1.60	a			-1.87	a	-1.46	a			-1.91	a	
			VDSDDQWGEYSCVFLPEPMGTANIQLHGPPR	C12(Carbamidomethyl)	1	-2.20	a											
P51572	B-cell receptor-associated protein 31	cytoplasm; cytosol; endoplasmic reticulum; Golgi; membrane	VNLQNNPGAMEHFHMK		2			-0.93	a	-3.66	a					-3.44	a	
Q16611	BCL2 antagonist killer 1	mitochondrion; endoplasmic reticulum; membrane	YDSEFQTMLQHLQPTAENAYEYFTK		1					-1.54	a							
Q9H3K6	BolA-like protein 2	extracellular vesicular exosome	MELSAEYLR	N-Term(Acetyl); M1(Oxidation)	1					2.07	a							
O75844	CAAX prenyl protease 1 homolog	cytoplasm; endoplasmic reticulum; Golgi; membrane	TTTHVPPELGQIMDSETFEK		1											-1.81	a	
Q6NUK1	Calcium-binding mitochondrial carrier protein SCaMC-1	cytoplasm; extracellular; membrane; mitochondrion	MNIFGGFR		1			0.00								-1.21	a	
			MNIFGGFR	M1(Oxidation)	1	1.61	a			2.24	a							
P62158	Calmodulin	cytoplasm; cytoskeleton; cytosol; extracellular; membrane; nucleus; organelle lumen	EADIDGDGQVNYEEFVQMMTAK	M18(Oxidation)	2	1.79	a			1.56	a							
			EADIDGDGQVNYEEFVQMMTAK	M18(Oxidation); M19(Oxidation)	2					4.34	a							
			EADIDGDGQVNYEEFVQMMTAK	M19(Oxidation)	2					1.32	a							
P04632	Calpain small subunit 1	cytoplasm; membrane; nucleus	GGGGGGGGGGLGGGLGNVLGGLISGAGGGGGGGGG GGGGGGGGGGTAMR	M50(Oxidation)	1					2.13	a							
			MFLVNSFLK	N-Term(Acetyl); M1(Oxidation)	1					1.40	a							
Q15417	Calponin-3	cytoskeleton	GASQAGMLAPGTR	M7(Oxidation)	1					1.47	a							
			GMSVYGLGR	M2(Oxidation)	1	1.24	a											
O43852	Calumenin	cytoplasm; endoplasmic reticulum; extracellular; Golgi; organelle	DIVVQETMEDIDK	M8(Oxidation)	1					3.18	a							
P10644	cAMP-dependent protein kinase type I-alpha regulatory subunit	cytoplasm; cytosol	MESGSTAASEEAR	N-Term(Acetyl); M1(Oxidation)	1					1.45	a							
P50416	Carnitine O-palmitoyltransferase 1, liver isoform	cytoplasm; membrane;	SEDPDTSMDSYAK		1			1.26	a									
P35221	Catenin alpha-1	cytoplasm; cytoskeleton; cytosol; membrane	MSASQLEALCPQVINAALALAAKPQSK	M1(Oxidation); C10(Carbamidomethyl)	1					3.24	a							#
O60716	Catenin delta-1	cytoplasm; cytosol; membrane; nucleus	DMDLTEVITGTLWNLSSHDSIK		1											-1.29	a	
			MEIVDHALHALTDEVIIPHSGWER		1	-2.29	a											
			QPELPEVIAMLGFR	M10(Oxidation)	1					2.17	a							
			QPELPEVIAMLGFR	N-Term(Gln->pyro-Glu); M10(Oxidation)	1					2.28	a							
			SMGYDDLDYGMMSDYGTAR		3					-1.65	a							
P07339	Cathepsin D	cytoplasm; extracellular; mitochondrion; vacuole	AIGAVPLIQGEYMIPCEK	C16(Carbamidomethyl)	1					-1.56	a							
			AIGAVPLIQGEYMIPCEK	M13(Oxidation); C16(Carbamidomethyl)	1					3.31	a							
			DPDAQPGGELMLGGTDSK	M11(Oxidation)	1			0.67	a	3.95	a							
			ISVNNVLPVFDNLMQQK	M14(Oxidation)	1			2.37	a									

P20645	Cation-dependent mannose-6-phosphate receptor	cell surface; cytoplasm; endosome; membrane; vacuole	GMEQFPHLAFWQDLGNLVADGCDFVCR	C22(Carbamidomethyl); C26(Carbamidomethyl)	1	-1.38	a											
			GVGDDQLGEESEERDDHLLPM		1	-1.39	a											
P16070	CD44 antigen	cell surface; cytoplasm; extracellular; Golgi; membrane;	ESSETPDQFMTADETR	M10(Oxidation)	1	3.71	a			4.03	a							
Q96JB5	CDK5 regulatory subunit-associated protein 3	centrosome ; cytoplasm ; endomembrane system ; membrane ; nucleus ; protein	KEEECQAGAAEMR	C5(Carbamidomethyl)	1			-0.79	a									
Q96S66	Chloride channel CLIC-like protein 1	cytoplasm; endoplasmic reticulum; Golgi; membrane; nucleus	GQMGPTEQGPYAK	M3(Oxidation)	1	1.75	a											
Q9Y696	Chloride intracellular channel protein 4	cell surface; cytoplasm;	ALSMPLNGLK	N-Term(Acetyl)	1			0.65	a									
		cytoskeleton; cytosol; membrane; mitochondrion; nucleus; organelle	ALSMPLNGLKEEDKEPLIELFVK	N-Term(Acetyl); M4(Oxidation)	1	1.71	a											
Q00610	Clathrin heavy chain 1	cytoplasm; cytoskeleton; cytosol; Golgi; membrane; mitochondrion	TLQIFNIEMK	M9(Oxidation)	1					1.56	a							
P48444	Coatomer subunit delta	cytoplasm; cytosol; Golgi;	MHAPPINMESVHMK		3					-1.24	a							
O14579	Coatomer subunit epsilon	cytoplasm; cytosol; Golgi;	MFADYLAHESR		1											-0.97	a	
P61923	Coatomer subunit zeta-1	cytoplasm; cytosol; Golgi;	MEALILEPSLYTVK	N-Term(Acetyl); M1(Oxidation)	1					2.59	a							
Q96A33	Coiled-coil domain-containing protein 47	cytoplasm; endoplasmic reticulum;	MMRPVSDQVQIK		2					-2.32	a							
		membrane	MVHFLTHYADKIESVHFSDQFSGPK		1	-1.84	a											
Q9NX63	Coiled-coil-helix-coiled-coil-helix domain-containing protein 3, mitochondrial	cytoplasm; membrane; mitochondrion	CSALATQYMHCVNHAK	C1(Carbamidomethyl); C11(Carbamidomethyl)	1					-2.79	a							
O75534	Cold shock domain-containing protein E1	cytoplasm	GPDNSMGFGAER	M6(Oxidation)	1					1.38	a							#
Q9UBI1	COMM domain-containing protein 3	Cytoplasm; nucleus	MELSESVQK	N-Term(Acetyl); M1(Oxidation)	1					2.23	a							
O75131	Copine-3	cytoplasm; cytosol	VECYDYDNDGSHDLIGTFQTTMTK	C3(Carbamidomethyl); M22(Oxidation)	1	1.14	a			1.99	a							
Q53SF7	Cordon-bleu protein-like 1	cell-cell adherens junction ; extracellular exosome	SMSVDETDKSPCEAGR	C12(Carbamidomethyl)	1					-2.11	a							
P22695	Cytochrome b-c1 complex subunit 2, mitochondrial	cytoplasm; membrane;	QVAEQFLNMR	M9(Oxidation)	1					4.24	a							
		mitochondrion; nucleus	QVAEQFLNMR	N-Term(Gln->pyro-Glu); M9(Oxidation)	1	1.76	a											
Q7L576	Cytoplasmic FMR1-interacting protein 1	cytoplasm; membrane	MADPQSIQESQNLSMFLANHNK		2											-1.21	a	
Q07065	Cytoskeleton-associated protein 4	cytoplasm; cytoskeleton;	LQHVEDGVLSMQVASAR		1					-2.10	a	-1.02	a					
		endoplasmic reticulum; membrane	SREWDMEALR		1	-1.33	a			-1.42	a					-1.96	a	
Q14126	Desmoglein-2	membrane; nucleus	QKPATETSMNTASHSLCEQTMVNSENTYSSGSSFPVPK	N-Term(Gln->pyro-Glu); C17(Carbamidomethyl)	2											-1.00	a	
			VLDINDNEPVFTQDVFVGSVEELSAAHTLVMK		1	-1.89	a			-1.70	a							
P15924	Desmoplakin	chromosome; cytoplasm; cytoskeleton; extracellular; membrane; mitochondrion;	QMGQPCDAYQK	N-Term(Gln->pyro-Glu); M2(Oxidation); C6(Carbamidomethyl)	1	2.24	a											
Q9P265	Disco-interacting protein 2 homolog B	nucleus	AQATPDHVLFMLLNAK		1											-1.28	a	
P26358	DNA (cytosine-5)-methyltransferase 1	chromosome; nucleus	EADDDEEVDDNIPEMPSPK		1			-0.64	a									
O15160	DNA-directed RNA polymerases I and III subunit RPAC1	nucleus; organelle lumen	AASQAVEEMR	N-Term(Acetyl); M9(Oxidation)	1					1.79	a							
P31689	DnaJ homolog subfamily A member 1	cytoplasm; cytoskeleton;	EVEETDEMDQVELVDFDPNQER	M8(Oxidation)	1	1.17	a											
Q13217	DnaJ homolog subfamily C member 3	cytoplasm; endoplasmic reticulum; membrane; organelle lumen	AEAYLIEEMYDEAIQDYETAQEHNENDQQIR		1					-1.23	a							
P39656	Dolichyl-diphosphooligosaccharide--protein glycosyltransferase 48 kDa subunit	cytoplasm; endoplasmic reticulum; membrane	MEPSTAAR		1											-1.13	a	
P50570	Dynamin-2	cytoplasm; cytoskeleton; cytosol; Golgi; membrane; nucleus	AGVYPEKDQAENEDGAQENTFSMDPQLER	M23(Oxidation)	1					1.75	a							
P49792	E3 SUMO-protein ligase RanBP2	cytoplasm; cytosol; membrane; nucleus	APGTNVAMASNQAVR	M8(Oxidation)	1					2.28	a							
Q9H4M9	EH domain-containing protein 1	cytoplasm; endosome; membrane	GGAFDGTMNGPFGHGYGEGAGEGIDDVEWVVGK		1	-1.27	a			-1.28	a							
Q9H223	EH domain-containing protein 4	cytoplasm; endoplasmic reticulum; endosome; membrane; nucleus	FMCSQLPNQVLK	M2(Oxidation); C3(Carbamidomethyl)	1					3.91	a							
P29692	Elongation factor 1-delta	cytoplasm; cytosol	FYEQMNGPVAGASR	M5(Oxidation)	1					1.75	a							
P26641	Elongation factor 1-gamma	cytoplasm; cytosol	AAAPAPEEEMDECEQALAAEPK	M10(Oxidation); C13(Carbamidomethyl)	1	1.43	a			1.61	a							
P13639	Elongation factor 2	cytoplasm; cytosol	MVNFTVDQIR		1			-1.09	a					-1.33	a			#
			QFAEMYVAK	M5(Oxidation)	1	1.68	a											#
			QFAEMYVAK	N-Term(Gln->pyro-Glu); M5(Oxidation)	1	1.93	a			2.36	a							#
Q9BW60	Elongation of very long chain fatty acids protein 1	cytoplasm; endoplasmic reticulum; membrane	MEAVVNLYQEVMK	N-Term(Acetyl)	2	-1.95	a			-2.56	a							
			MEAVVNLYQEVMK	N-Term(Acetyl); M1(Oxidation)	2					2.52	a							
Q9NYP7	Elongation of very long chain fatty acids protein 5	cytoplasm; endoplasmic reticulum; membrane	MEHFDASLSTYFK	N-Term(Acetyl)	1			-0.61	a									
Q9Y371	Endophilin-B1	cytoplasm; Golgi; membrane; mitochondrion; nucleus	MNIMDFNVK	N-Term(Acetyl)	2					-1.21	a							
Q9NZ08	Endoplasmic reticulum aminopeptidase 1	cytoplasm; cytosol; endoplasmic reticulum; extracellular; membrane; organelle lumen	HETEIMPVFQGLNELIPMYK		2					-1.56	a					-1.62	a	

Q7Z2K6	Endoplasmic reticulum metallopeptidase 1	cytoplasm; endoplasmic reticulum; membrane	HLATSDMLAAASK		1					-1.72	a							
P30040	Endoplasmic reticulum resident protein 29	cytoplasm; endoplasmic reticulum; organelle lumen	LAENSASSDDLLVAEVGISDYGDKLNMELSEK	M27(Oxidation)	1					2.71	a							
Q9BS26	Endoplasmic reticulum resident protein 44	cytoplasm; endoplasmic reticulum; membrane; organelle lumen	EITFENGEELTEEGLPFLILFHMK	M23(Oxidation)	1	1.43	a											
			FSQMLHPIFEEASDVIKEEFPNENQVVFAR		1					-2.04	a							
P14625	Endoplasmin	cytoplasm; cytosol; endoplasmic reticulum; membrane; organelle lumen	LTESPCALVASQYGWSGNMER	C6(Carbamidomethyl)	1											-1.10	a	
			LTESPCALVASQYGWSGNMER	C6(Carbamidomethyl); M19(Oxidation)	1					3.05	a							
			NLLHVTDTGVGMTR		1					-1.32	a					-1.35	a	
			RVFITDDFHDMMPK		2					-1.35	a							
			TETVEEPMEEEEAAK	M8(Oxidation)	1					2.09	a							
			TETVEEPMEEEEAAKEEK	M8(Oxidation)	1					2.75	a							
			TVWDWELMNDIKPIWQRPSK		1											-1.58	a	
			VFITDDFHDMMPK		2					-1.21	a					-1.05	a	
P00533	Epidermal growth factor receptor	cytoplasm; endoplasmic reticulum; endosome; extracellular; Golgi; membrane; nucleus	ACGADSYEMEEDGVR	C2(Carbamidomethyl); M9(Oxidation)	1					3.55	a							#
			ACGADSYEMEEDGVRK	C2(Carbamidomethyl); M9(Oxidation)	1					3.43	a							#
			ALMDEEDMDDVVDADEYLIPQQGFFSSPSTSR		2	-1.86	a											#
			DIVSSDFLSNMSMDFQNHLGSCQK	C22(Carbamidomethyl)	2							-1.41	a					#
			DTCPPLMLYNPTTYQMDVNPEGK	C3(Carbamidomethyl)	2	-1.27	a			-1.39	a	-1.18	a			-1.59	a	#
			DTCPPLMLYNPTTYQMDVNPEGK	C3(Carbamidomethyl); M7(Oxidation)	2					1.34	a							#
			DTCPPLMLYNPTTYQMDVNPEGK	C3(Carbamidomethyl); M7(Oxidation); M16(Oxidation)	2	4.73	a	2.91	a									#
			EILDEAYVMASVDNPHVCR	M9(Oxidation); C18(Carbamidomethyl)	1					1.87	a							#
			GNMYYENSYALAVLSNYDANK	M3(Oxidation)	1	1.98	a			3.07	a							#
			MHLPSPTDSNFYR		1	-1.89	a											#
			WMALESILHR		1	-1.34	a			-1.32	a	-1.24	a			-1.35	a	#
P61916	Epididymal secretory protein E1	cytoplasm; extracellular; nucleus; vacuole	AVVHGILMGVPVPFPIPEPDGCK	M8(Oxidation); C22(Carbamidomethyl)	1			1.36	a									
P16422	Epithelial cell adhesion molecule	cell surface; membrane	KMDLTVNGEQLDLDPGQTLIYYVDEK	M2(Oxidation)	1	2.60	a											
Q96HE7	ERO1-like protein alpha	cytoplasm; endoplasmic reticulum; membrane; organelle lumen	MLLLEILHEIK		1											-1.86	a	#
P60842	Eukaryotic initiation factor 4A-I	cytoplasm; cytosol	LNSNTQVVLLSATMPSDVLEVTKK	M14(Oxidation)	1					1.53	a							#
P05198	Eukaryotic translation initiation factor 2 subunit 1	cytoplasm; cytosol; nucleus	ENAEVDGDDDAEEMEAKAED	M14(Oxidation)	1					2.72	a							
O15371	Eukaryotic translation initiation factor 3 subunit D	cytoplasm; cytosol	NMLQFNLQILPK	M2(Oxidation)	1					2.13	a							
O15372	Eukaryotic translation initiation factor 3 subunit H	cytoplasm; cytosol	LEITNCFPFPQHTEDDADFDEVQYQMEMMR	C6(Carbamidomethyl); M26(Oxidation); M29(Oxidation)	3	1.47	a											
P78344	Eukaryotic translation initiation factor 4 gamma 2	cytoplasm; cytosol	MESAIAEGGASR	N-Term(Acetyl); M1(Oxidation)	1	1.88	a											
Q15056	Eukaryotic translation initiation factor 4H	cytoplasm; cytosol	GSNMDFREPTEEER	M4(Oxidation)	1					1.95	a							
P55010	Eukaryotic translation initiation factor 5	cytoplasm; cytosol	AMGPLVLTEVLFNEK	M2(Oxidation)	1	1.58	a			1.81	a							
P15311	Ezrin	cytoplasm; cytoskeleton; cytosol; membrane; nucleus; organelle	ILQLCMGNHELYMR	C5(Carbamidomethyl)	2	-1.31	a									-1.11	a	#
			THNDIIHNENMR		1	-1.24	a					-1.36	a			-1.23	a	#
P47756	F-actin-capping protein subunit beta	cytoplasm; cytoskeleton; cytosol; membrane	QMEKDETVSDCSPHIANIGR	M2(Oxidation); C11(Carbamidomethyl)	1					1.35	a							#
Q96CS3	FAS-associated factor 2	cytoplasm; endoplasmic reticulum; membrane	VTDPVGDIVSFMHSFEEK		1					-1.88	a							
P49327	Fatty acid synthase	cytoplasm; cytosol; Golgi; membrane; mitochondrion	EAHLPPGAMAAVGLSWEECK	C19(Carbamidomethyl)	1							-1.21	a					#
			LGMLSPEGTCK	M3(Oxidation); C10(Carbamidomethyl)	1					1.45	a							#
O75955	Flotillin-1	cytoplasm; cytoskeleton; endosome; Golgi; membrane;	SPPVMVAGGR	M5(Oxidation)	1					4.43	a							
Q16666	Gamma-interferon-inducible protein 16	cytoplasm; nucleus; organelle	TPQMPPTTPSSSFFTK	M4(Oxidation)	1	1.38	a											
O60763	General vesicular transport factor p115	cytoplasm; cytosol; Golgi;	LEVGIQAMEHLIHVLQTDR		1											-1.20	a	
P14314	Glucosidase 2 subunit beta	cytoplasm; endoplasmic reticulum; organelle lumen	SLEDQVEMLR	M8(Oxidation)	1					3.40	a							#
Q9Y2Q3	Glutathione S-transferase kappa 1	cytoplasm; membrane; mitochondrion; organelle lumen	YGAFGLPITVAHVDGQTHMLFGSDR		1					-1.20	a							
P43304	Glycerol-3-phosphate dehydrogenase	cytoplasm; membrane;	VLESINVQMDENTLHEILNEVDLNK		1					-1.58	a					-1.55	a	
P28799	Granulins	cytoplasm; extracellular; mitochondrion	CDMEVSCPDGYTCCR	C1(Carbamidomethyl); M3(Oxidation); C7(Carbamidomethyl); C13(Carbamidomethyl); C14(Carbamidomethyl)	1					3.90	a							
Q9HAV7	GrpE protein homolog 1, mitochondrial	cytoplasm; mitochondrion; organelle lumen	NSGQNLEEDMGQSEQK	M10(Oxidation)	1					1.42	a							
			NSGQNLEEDMGQSEQKADPPATEK	M10(Oxidation)	1					2.44	a							
P01111	GTPase NRas	cytoplasm; Golgi; membrane	QVVIDGETCLLDILDTAGQEEYSAMR	C9(Carbamidomethyl)	1					-1.14	a							
			QVVIDGETCLLDILDTAGQEEYSAMR	C9(Carbamidomethyl); M25(Oxidation)	1	2.22	a											
Q14C86	GTPase-activating protein and VPS9 domain-containing protein 1	cytoplasm; cytosol; endosome; membrane	FNLMQVGR	M4(Oxidation)	1					2.03	a							
Q9NR31	GTP-binding protein SAR1a	cytoplasm; endoplasmic reticulum; Golgi	LGQHVPTLHPTSEELTIAGMTFTTFDLGGHEQAR		1	-1.44	a											

Q9UBI6	Guanine nucleotide-binding protein G(I)/G(S)/G(O) subunit gamma-12	cytoskeleton; membrane	ASADLMSYCEEHAR	C9(Carbamidomethyl)	1					-1.19	a			
P62873	Guanine nucleotide-binding protein G(I)/G(S)/G(T) subunit beta-1	membrane	ADQELMTYSHDNIICGITSVSFSK	C15(Carbamidomethyl)	1	-2.43	a			-2.41	a			
P62879	Guanine nucleotide-binding protein G(I)/G(S)/G(T) subunit beta-2	cytoplasm; membrane	ADQELLMYSHDNIICGITSVAFSR	C15(Carbamidomethyl)	1	-1.76	a					-2.23	a	
P63244	Guanine nucleotide-binding protein subunit beta-2-like 1	cytoplasm; cytoskeleton; membrane; nucleus; ribosome	GHNGWVTQIATTPQFPDMILSASR	M18(Oxidation)	1					1.85	a			
P34932	Heat shock 70 kDa protein 4	cytoplasm; cytosol; nucleus; organelle lumen	NAVEEYVYEMR	M10(Oxidation)	1					1.27	a			
P11142	Heat shock cognate 71 kDa protein	cell surface; cytoplasm; cytosol; membrane	NQVAMNPTNTVFDAK	M5(Oxidation)	1	1.21	a			1.70	a			#
			NSLESYAFNMK	M10(Oxidation)	1					1.99	a			#
P07900	Heat shock protein HSP 90-alpha	cytoplasm; cytosol; membrane	LGLGIDEDDPTADDTSAAVTEEMPPLEGDDDTSR	M23(Oxidation)	1					1.28	a			#
			YYTSASGDEMVSLK	M10(Oxidation)	1	1.71	a			1.20	a			#
P08238	Heat shock protein HSP 90-beta	cytoplasm; cytosol; membrane; mitochondrion; nucleus	DNSTMGYMMAK	M8(Oxidation); M9(Oxidation)	3	2.45	a							#
			LVSSPCCIVTSTYGWTANMER	C6(Carbamidomethyl); C7(Carbamidomethyl); M19(Oxidation)	1					2.12	a			#
			VFIMDSCDELIPEYLNFIR	M4(Oxidation); C7(Carbamidomethyl)	1	1.16	a							#
P38159	Heterogeneous nuclear ribonucleoprotein G	chromosome; extracellular; nucleus; organelle lumen; spliceosomal complex	MVEADRPGK	N-Term(Acetyl)	1			-0.97	a					
P31943	Heterogeneous nuclear ribonucleoprotein H	cytoplasm; cytoskeleton; nucleus; organelle lumen; spliceosomal complex	MMLGTEGGEGFVVK	N-Term(Acetyl); M1(Oxidation); M2(Oxidation)	2					2.24	a			
P61978	Heterogeneous nuclear ribonucleoprotein K	chromosome; cytoplasm; nucleus; organelle lumen; spliceosomal complex	AQPYDPNFYDETYDYGGFTMMFDDRR	M20(Oxidation); M21(Oxidation)	2	2.27	a							
P52272	Heterogeneous nuclear ribonucleoprotein M	membrane; nucleus; organelle lumen; spliceosomal complex	MGAGMGFGLER	M1(Oxidation); M5(Oxidation)	2	1.97	a			2.02	a			
			MGGMEGPFGGGMENMGR	M12(Oxidation); M15(Oxidation)	4					2.65	a			
			MGPAMGPALGAGIER	M1(Oxidation); M5(Oxidation)	2	1.79	a			2.48	a			
O43390	Heterogeneous nuclear ribonucleoprotein R	cytoplasm; nucleus; organelle lumen; spliceosomal complex	LMMDPLSGQNR	M2(Oxidation); M3(Oxidation)	2	1.26	a							
Q00839	Heterogeneous nuclear ribonucleoprotein U	cell surface; cytoplasm; nucleus; organelle lumen; spliceosomal complex	MCLFAGFQR	M1(Oxidation); C2(Carbamidomethyl)	1	1.24	a							
P22626	Heterogeneous nuclear ribonucleoproteins A2/B1	cytoplasm; nucleus; organelle lumen; spliceosomal complex	NMGGPYGGGNYGPGGSGGSGGYGGR	M2(Oxidation)	1	1.33	a			1.18	a			
P19367	Hexokinase-1	cytoplasm; cytosol; membrane; mitochondrion; nucleus; organelle lumen	MVSGMYLGELVR	M5(Oxidation)	2					2.64	a			
O60814	Histone 1 H2bk	Nucleus ; Nucleolus	AMGIMNSFVNDIFER	M2(Oxidation); M5(Oxidation)	2	1.48	a							
P62805	Histone H4	chromosome; extracellular; nucleus; organelle lumen	TVTAMDVVYALK	M5(Oxidation)	1	1.34	a							
P10321	HLA class I histocompatibility antigen, Cw-7 alpha chain	cytoplasm; cytoskeleton; endoplasmic reticulum; endosome; extracellular; Golgi; membrane	MSGCDLGPDGR	M1(Oxidation); C4(Carbamidomethyl)	1	2.44	a							
Q16543	Hsp90 co-chaperone Cdc37	cytoplasm; cytosol	QYMEGFNDELEAFK	N-Term(Gln->pyro-Glu); M3(Oxidation)	1	1.35	a							
Q9Y4L1	Hypoxia up-regulated protein 1	cytoplasm; endoplasmic reticulum; organelle lumen	VAIVKPGVPMEIVLNK		1					-1.20	a	-1.24	a	
			VAIVKPGVPMEIVLNK	M10(Oxidation)	1					4.07	a			
O00410	Importin-5	cytoplasm; membrane; nucleus; organelle lumen	EFQQYLPVVMGPLMK	M10(Oxidation); M14(Oxidation)	2	1.44	a							
			SELLMIIQMETQSSMR	M5(Oxidation); M9(Oxidation)	3	2.06	a							
O95373	Importin-7	cytoplasm; Golgi; membrane;	MDPNTIIEALR	N-Term(Acetyl); M1(Oxidation)	1					1.63	a			
P05556	Integrin beta-1	cell surface; cytoplasm; endoplasmic reticulum; extracellular; membrane	HCECSTDEVNSEDMDAYCR	C2(Carbamidomethyl); C4(Carbamidomethyl); C18(Carbamidomethyl)	1							-1.97	a	#
P16144	Integrin beta-4	cell surface; membrane	KQDHTIVDTVLMAPR		1							-2.54	a	#
P14923	Junction plakoglobin	cytoplasm; cytoskeleton; cytosol; membrane; nucleus; organelle	EAADAIDAEGASAPLMELLHSR		1					-1.31	a	-1.10	a	
			VAAGVLCELAQDKEAADAIDAEGASAPLMELLHSR	C7(Carbamidomethyl)	1					-1.39	a			
Q9Y624	Junctional adhesion molecule A	membrane	EDTGTYTCMVSEEGGNSYGEVK	C8(Carbamidomethyl); M9(Oxidation)	1	4.08	a			4.11	a			
P02545	Lamin-A/C	cytoplasm; cytoskeleton; membrane; nucleus; organelle	LQTMKEELDFQK	M4(Oxidation)	1					3.58	a			
P20700	Lamin-B1	cytoplasm; cytoskeleton; membrane; mitochondrion; nucleus; organelle lumen	DQMQQQLNDYEQLLDVK	M3(Oxidation)	1			0.85	a					
			LAQALHEMR		1					-1.74	a			

Q13751	Laminin subunit beta-3	extracellular	AGGAFLMAGQVAEQLR	M7(Oxidation)	1					3.11	a							
Q13753	Laminin subunit gamma-2	cytoplasm; cytoskeleton; extracellular; membrane	ACNCNPMGSEPVGCR	C2(Carbamidomethyl); C4(Carbamidomethyl); M7(Oxidation); C14(Carbamidomethyl)	1	2.56	a			3.11	a							
Q9NS86	LanC-like protein 2	cytoplasm; cytoskeleton; cytosol; membrane; nucleus	VDQETLTEMVKPSIDYVR	M9(Oxidation)	1					2.14	a							#
P42704	Leucine-rich PPR motif-containing protein, mitochondrial	chromosome; cytoplasm; cytoskeleton; membrane; mitochondrion; nucleus; organelle lumen	GMQELGVHPDQETYTDYVIPCFDSVNSAR	C21(Carbamidomethyl)	1											-1.01	a	
P30740	Leukocyte elastase inhibitor	cytoplasm; extracellular	MEQLSSANTR	N-Term(Acetyl); M1(Oxidation)	1	1.65	a											
P00338	L-lactate dehydrogenase A chain	cytoplasm; cytosol; mitochondrion	LKGEMMDLQHGSLFLR	M5(Oxidation); M6(Oxidation)	2	1.63	a											
P07195	L-lactate dehydrogenase B chain	cytoplasm; cytosol; mitochondrion	GMYGIENEVFLSLPCILNAR	M2(Oxidation); C15(Carbamidomethyl)	1					1.84	a							
P33121	Long-chain-fatty-acid--CoA ligase 1	cytoplasm; cytosol; endoplasmic reticulum; membrane;	MQAHELFR	N-Term(Acetyl)	1					-2.40	a							
P40926	Malate dehydrogenase, mitochondrial	cytoplasm; membrane; mitochondrion; nucleus; organelle lumen	FVFSLVDAMNGK	M9(Oxidation)	1					2.23	a							#
O15173	Membrane-associated progesterone receptor	membrane	DEYDDLSDLNAVQMESVR	M14(Oxidation)	1					2.53	a							
P02795	Metallothionein-2	cytoplasm; cytosol; nucleus	MDPNCSCAAGDSCTCAGSCK	M1(Oxidation); N-Term(Acetyl); C5(Carbamidomethyl); C7(Carbamidomethyl); C13(Carbamidomethyl); C15(Carbamidomethyl); C19(Carbamidomethyl)	1					1.27	a							
Q8TCT9	Minor histocompatibility antigen H13	cell surface; cytoplasm; endoplasmic reticulum; membrane	GEVTEMFSYEESNPKDPAAVTESK	M6(Oxidation)	1					1.35	a							
Q9Y5L4	Mitochondrial import inner membrane translocase subunit Tim13	cytoplasm; membrane; mitochondrion	MEGGFGSDFGGSGSGK	N-Term(Acetyl); M1(Oxidation)	1	2.01	a			2.81	a							
Q9NS69	Mitochondrial import receptor subunit TOM22 homolog	cytoplasm; membrane;	LQMEQQQQLQQR	M3(Oxidation)	1	1.91	a			3.49	a							
O96008	Mitochondrial import receptor subunit TOM40 homolog	cytoplasm; membrane;	RPGEEGTVMSLAGK		1					-1.49	a					-1.30	a	
			RPGEEGTVMSLAGK	M9(Oxidation)	1	1.86	a	0.60	a									
O94826	Mitochondrial import receptor subunit TOM70	cytoplasm; membrane;	ASPAPGSGHPEGPGAHLDMNSLDR		1											-1.87	a	
			QAYTGNNSSQIQAAMK	M15(Oxidation)	1					2.50	a							
			SEMEMAHLYSLCDAAHAQTEVAK	C12(Carbamidomethyl)	2					-1.81	a					-1.37	a	
Q16891	Mitochondrial inner membrane protein	cytoplasm; membrane;	LFEMVLGPAAYNVPLPK	M4(Oxidation)	1	1.87	a	1.34	a									
			LHNMIVDLDNVVK		1					-1.82	a					-1.65	a	
			LHNMIVDLDNVVKK		1					-1.69	a					-1.79	a	
Q10713	Mitochondrial-processing peptidase subunit alpha	cytoplasm; membrane; mitochondrion; organelle lumen	DTTMYAVSADSK	M4(Oxidation)	1	1.51	a											
P28482	Mitogen-activated protein kinase 1	cytoplasm; cytoskeleton; cytosol; endosome; Golgi; membrane; mitochondrion; nucleus; organelle lumen	AAAAAAGAGPEMVR	N-Term(Acetyl); M12(Oxidation)	1	2.04	a			1.96	a							
P26038	Moesin	cell surface; cytoplasm; cytoskeleton; membrane; nucleus;	GMLREDAVLEYLK	M2(Oxidation)	1	1.81	a			1.95	a							#
			QRIDEFESM	M9(Oxidation)	1	2.08	a			2.85	a							#
P33527	Multidrug resistance-associated protein 1	cytoplasm; Golgi; membrane; nucleus; organelle lumen	SSLLSALLAEMDKVEGHVAIK		1	-1.45	a											
Q8NI22	Multiple coagulation factor deficiency protein 2	cytoplasm; endoplasmic reticulum; extracellular; Golgi; membrane	MHDYDGNNLLDGLELSTAITHVHK		1	-1.41	a											
Q9NZM1	Myoferlin	cytoplasm; cytoskeleton; membrane; nucleus	DIVIEMEDTKPLLASK	M6(Oxidation)	1	1.79	a			2.75	a							
P35579	Myosin-9	cytoplasm; cytoskeleton; cytosol; membrane; mitochondrion; nucleus	ALEQQVEEMKTQLEELEDELQATEDAK	M9(Oxidation)	1					2.41	a							#
			IIGLDQVAGMSETALPGAFK	M10(Oxidation)	1					1.74	a							#
			KMEDSVGCLETAEEVKR	M2(Oxidation); C8(Carbamidomethyl)	1					1.50	a							#
			TEMEDLMSSKDDVGK	M3(Oxidation); M7(Oxidation)	2	2.32	a			4.04	a							#
P15586	N-acetylglucosamine-6-sulfatase	cytoplasm; vacuole	LMMLQSCSGPTCR	C7(Carbamidomethyl); C12(Carbamidomethyl)	2					-1.76	a							
O95169	NADH dehydrogenase [ubiquinone] 1 beta subcomplex subunit 8	cytoplasm; endoplasmic reticulum; membrane; mitochondrion	VEDYEPYPDDGMGYGDYPK	M12(Oxidation)	1	2.21	a											
P00387	NADH-cytochrome b5 reductase 3	cytoplasm; cytosol; endoplasmic reticulum; membrane;	AIMKDPDDHTVCHLLFANQTEK	C12(Carbamidomethyl)	1	-1.38	a			-2.06	a					-1.99	a	
Q09666	Neuroblast differentiation-associated protein AHNAK	nucleus	GPGVDLPSVNLSMPK	M13(Oxidation)	1	2.35	a											
			IPMPDFDLHLK		1	0.66				-3.73	a							
			ISMPDFDLHLK		1	-1.21	a											
			ISMPDVDLHLK		1							-1.16	a					
			MDIDAPDVDVHGPDWHLK		1	-1.80	a											
			VEAPSLDVHMDSPDINIEGPDVK		1	-1.40	a					-1.40	a					

Q14697	Neutral alpha-glucosidase AB	cytoplasm; endoplasmic reticulum; Golgi; organelle lumen	MMDYLQGSGETPQTDVR		2					-1.27	a			-1.16	a	#
Q96TA1	Niban-like protein 1	cytoplasm; cytosol; membrane; nucleus	EQMDNAVYTFETLLHQELGK		1	-2.25	a			-1.61	a	-1.72	a	-1.94	a	
			NHVQPYIPSILEALMVPTSQGFTEVR		1	-1.17	a					-1.11	a			
Q15233	Non-POU domain-containing octamer-binding protein	nucleus; organelle lumen	MGQMAMGGAMGINNR	M1(Oxidation); M6(Oxidation); M10(Oxidation)	4					2.06	a					
			MGQMAMGGAMGINNR	M4(Oxidation); M6(Oxidation); M10(Oxidation)	4	1.44	a			2.41	a					
Q8WUM0	Nuclear pore complex protein Nup133	chromosome; cytoplasm; cytosol; membrane; nucleus	IHQHILPQGQGMLSGIGR		1									-2.01	a	
O75694	Nuclear pore complex protein Nup155	membrane; nucleus	QYSHHSSVQDAVSQLDSELMDITK	N-Term(Gln->pyro-Glu)	1									-1.13	a	
Q12769	Nuclear pore complex protein Nup160	chromosome; cytoplasm; cytosol; membrane; nucleus	EMDYETEVEMEK	M10(Oxidation)	2					2.20	a					
P35658	Nuclear pore complex protein Nup214	cytoplasm; cytosol; membrane; nucleus; organelle lumen	SHLVHGSSPGVMGTSVATSASK		1							-1.24	a			
Q9BW27	Nuclear pore complex protein Nup85	chromosome; cytoplasm; cytoskeleton; cytosol; membrane; nucleus	SFWMTLLTDALPLLEQK	M4(Oxidation)	1					1.54	a					
P80303	Nucleobindin-2	cytoplasm; cytosol; endoplasmic reticulum; extracellular; Golgi; membrane; nucleus	NEEDDMVEMEEER		2					-1.19	a					
			NEEDDMVEMEEER	M6(Oxidation); M9(Oxidation)	2			2.19	a							
Q96RS6	NudC domain-containing protein 1	cytoplasm, nucleus, membrane	MEVAANCSLR	N-Term(Acetyl); M1(Oxidation); C7(Carbamidomethyl)	1	1.43	a			1.12	a					
P22059	Oxysterol-binding protein 1	cytoplasm; Golgi; membrane	IPMPVNFNEPLSMLQR	M3(Oxidation)	2					2.51	a					
Q9BRP8	Partner of Y14 and mago	cytoplasm; nucleus; organelle	MEAAGSPAATETGK	N-Term(Acetyl); M1(Oxidation)	1	1.40	a			1.21	a					
P62937	Peptidyl-prolyl cis-trans isomerase A	cytoplasm; cytosol; extracellular; nucleus	MVNPTVFFDIAVDGEPLGR		1			-0.66	a							
			MVNPTVFFDIAVDGEPLGR	N-Term(Acetyl); M1(Oxidation)	1			-0.62	a							
			VKEGMNIVEAMER	M5(Oxidation)	2					2.05	a					
P23284	Peptidyl-prolyl cis-trans isomerase B	cytoplasm; endoplasmic reticulum; organelle lumen	VLEGMEVVR	M5(Oxidation)	1					2.73	a					
Q02790	Peptidyl-prolyl cis-trans isomerase FKBP4	cytoplasm; cytoskeleton; cytosol; membrane; nucleus; organelle	ATESGAQSAPLPMEGVDISPK	M13(Oxidation)	1	1.49	a									
O60664	Perilipin-3	cytoplasm; endosome; Golgi; membrane	SVVTGGVQSVMGSR	M11(Oxidation)	1					2.74	a					
Q00325	Phosphate carrier protein, mitochondrial	cytoplasm; membrane;	GVAPLWMR	M7(Oxidation)	1					2.95	a					
Q9NTJ5	Phosphatidylinositide phosphatase SAC1	cytoplasm; endoplasmic reticulum; Golgi; membrane	GSEKPLEQTFATMVSSLGSGMMR		3					-2.07	a					
			THLGLIMDGWNSMIR		2									-2.57	a	
P48739	Phosphatidylinositol transfer protein beta isoform	cytoplasm; Golgi; nucleus	MIAPEGSLVFHEK		1					-1.31	a					
O95394	Phosphoacetylglucosamine mutase	cytoplasm; cytosol	MGLLAVLR	M1(Oxidation)	1	2.46	a									
P00558	Phosphoglycerate kinase 1	cytoplasm; cytosol	VNEMIIGGGMAFTFLK	M4(Oxidation); M10(Oxidation)	2	1.11	a									#
P78330	Phosphoserine phosphatase	cytoplasm; cytosol; membrane	MVSHSELR	N-Term(Acetyl)	1			-0.68	a							
Q99959	Plakophilin-2	cytoskeleton; membrane; nucleus	AVSMLEADHMLPSR		2									-1.66	a	
Q8NC51	Plasminogen activator inhibitor 1 RNA-binding protein	cytoplasm; membrane; nucleus	SEEAHAEDSVMDHHFR		1									-1.50	a	#
			SKSEEAHAEDSVMDHHFR		1	-1.92	a									#
P13796	Plastin-2	cytoplasm; cytoskeleton; cytosol; extracellular; membrane	EGESLEDLMK	M9(Oxidation)	1					2.31	a					#
			GSVSDEEMMELR		2									-0.95	a	#
O15031	Plexin-B2	cytoplasm; cytoskeleton;	QMVQVSDQDMNTHLAEISR		2					-2.50	a					
P11940	Polyadenylate-binding protein 1	cytoplasm; cytosol; nucleus; spliceosomal complex	QAHLTNQYMQR	N-Term(Gln->pyro-Glu); M9(Oxidation)	1					1.30	a					#
Q6NZI2	Polymerase I and transcript release factor	cytoplasm; cytosol; endoplasmic reticulum; membrane; mitochondrion; nucleus; organelle	ATEMVEVGADDDEGGAER	M4(Oxidation)	1					3.35	a					
Q14435	Polypeptide N-acetylgalactosaminyltransferase 3	cytoplasm; Golgi; membrane;	MLDLMLEAVNNIK		2									-2.54	a	
			MQIGAPVR		1					-2.19	a					
O75915	PRA1 family protein 3	cytoplasm; endoplasmic reticulum; membrane	MDVNIAPLR	N-Term(Acetyl)	1					-1.43	a					
Q9UHG3	Prenylcysteine oxidase 1	cytoplasm; extracellular; membrane; vacuole	MHMWVEDVLDK		2			-0.66	a							
P07602	Proactivator polypeptide	cytoplasm; extracellular; Golgi; membrane; mitochondrion; organelle lumen; vacuole	LGPGMADICK	C9(Carbamidomethyl)	1							-1.08	a			
			LGPGMADICK	M5(Oxidation); C9(Carbamidomethyl)	1					2.09	a					
P12004	Proliferating cell nuclear antigen	chromosome; cytoplasm; cytoskeleton; nucleus; organelle lumen	LMDLDVEQLGIPEQEYSCVVK	M2(Oxidation); C18(Carbamidomethyl)	1					1.16	a					
Q5VYK3	Proteasome-associated protein ECM29 homolog	cytoplasm; cytoskeleton; cytosol; endoplasmic reticulum; endosome; Golgi; nucleus; proteasome	EVLPLAFLGMHEIADEEK		1							-1.29	a			

O14744	Protein arginine N-methyltransferase 5	cytoplasm; cytosol; nucleus	AAMAVGGAGGSR	N-Term(Acetyl); M3(Oxidation)	1	1.51	a			1.86	a							
Q9Y2B0	Protein canopy homolog 2	cytoplasm; endoplasmic reticulum; membrane	MKEYGEQIDPSTHR		1					-2.18	a					-2.03	a	
Q9UKY7	Protein CDV3 homolog	cytoplasm; nucleus; organelle	VQAMQISSEKEEDDNEK	M4(Oxidation)	1					1.71	a							
P07237	Protein disulfide-isomerase	cell surface; cytoplasm; endoplasmic reticulum; extracellular; membrane; organelle	IKPHLMSQELPEDWDKQPVK		1											-1.16	a	#
P49257	Protein ERGIC-53	cytoplasm; endoplasmic reticulum; Golgi; membrane	LVSGMQHPGSAGGVYETTQHFIDIK		1											-3.27	a	
Q13158	Protein FADD	cytoplasm; cytosol; Golgi; membrane; nucleus	SCQMNLVADLVQEVQQAR	C2(Carbamidomethyl); M4(Oxidation)	1	2.53	a											
Q96A26	Protein FAM162A	cytoplasm; membrane;	EDEIPETVSLEMLDAAK	M12(Oxidation)	1	2.65	a			5.05	a							
			FKKEDEIPETVSLEMLDAAK		1											1.17	a	
Q92597	Protein NDRG1	cytoplasm; cytoskeleton; cytosol; endosome; membrane; nucleus	SIIGMGTGAGAYILTR		1					1.20	a							
P60903	Protein S100-A10	cytoplasm; membrane;	MPSQMEHAMETMMFTFHK		5											-1.32	a	
			PSQMEHAMETMMFTFHK		4	-1.69	a			-1.43	a					-1.30	a	
			PSQMEHAMETMMFTFHK	M11(Oxidation)	4	-1.69	a											
Q9HCY8	Protein S100-A14	cytoplasm; cytoskeleton;	DLVTQQLPHLMPSNCGLEEK	C15(Carbamidomethyl)	1	-2.06	a			-1.97	a					-1.94	a	
P55735	Protein SEC13 homolog	chromosome; cytoplasm; cytosol; endoplasmic reticulum; membrane; nucleus	MVSVINTVDTSHEDMIHDAQMDYYGTR	N-Term(Acetyl)	3									-1.30	a			
Q92734	Protein TFG	cytoplasm; membrane	MNGQLDLSGK	N-Term(Acetyl); M1(Oxidation)	1	1.50	a											
Q15437	Protein transport protein Sec23B	cytoplasm; endoplasmic reticulum; Golgi; membrane	MVVPLACLLTPLK	M1(Oxidation); C7(Carbamidomethyl)	1					3.32	a							
P60468	Protein transport protein Sec61 subunit beta	cytoplasm; endoplasmic reticulum; membrane	TTSAGTGGMWR		1					-1.19	a							
P22061	Protein-L-isoaspartate(D-aspartate) O-methyltransferase	cytoplasm; endoplasmic reticulum	MGYAEEAPYDAIHVGAAAPVVPQALIDQLKPGGR	M1(Oxidation)	1					1.98	a							#
Q7L7V1	Putative pre-mRNA-splicing factor ATP-dependent RNA helicase DHX32	cytoplasm; mitochondrion; nucleus	EQQMCETCPETEQR	C5(Carbamidomethyl); C8(Carbamidomethyl)	1					-1.61	a							
P50395	Rab GDP dissociation inhibitor beta	cell surface; cytoplasm; cytosol; Golgi; membrane	IPGSPPESMGR	M9(Oxidation)	1					1.32	a							#
P62491	Ras-related protein Rab-11A	cytoplasm; endosome; Golgi; membrane; mitochondrion	DHADSNIVIMLVGNK		1							-0.96	a					
Q9H0U4	Ras-related protein Rab-1B	cytoplasm; Golgi; membrane; mitochondrion; nucleus	NATNVEQAFMTMAAEIK	M10(Oxidation)	2	2.30	a											
P61019	Ras-related protein Rab-2A	cytoplasm; endoplasmic reticulum; Golgi; membrane; nucleus	EHGLIFMETSAK		1					-1.39	a					-1.25	a	
			QHSNSNMVIMLIGNK		2											-2.05	a	
			QHSNSNMVIMLIGNK	N-Term(Gln->pyro-Glu)	2											-1.22	a	
Q15286	Ras-related protein Rab-35	cytoplasm; endosome; extracellular; membrane;	FAGQMGIQLFETSAK	M5(Oxidation)	1	2.33	a			3.35	a							
P51148	Ras-related protein Rab-5C	cytoplasm; endosome; membrane; nucleus	AVEFQEAQAYADDNSLLFMETSAK		1					-1.78	a					-1.32	a	
			TAMNVNEIFMAIAK		2											-0.92	a	
P62070	Ras-related protein R-Ras2	cytoplasm; endoplasmic reticulum; membrane	MNVDQAFHELVR		1					-1.52	a					-1.46	a	
Q9HAU5	Regulator of nonsense transcripts 2	cytoplasm; cytosol; nucleus	QQEEEAAAQMK	N-Term(Gln->pyro-Glu); M10(Oxidation)	1					1.34	a							
P52566	Rho GDP-dissociation inhibitor 2	cytoplasm; cytoskeleton; cytosol	ELQEMDKDDESLIK	M5(Oxidation)	1					1.90	a							
O75116	Rho-associated protein kinase 2	cytoplasm; cytoskeleton; cytosol; membrane; mitochondrion;	MPGAPETAPGDGAGASR	M1(Oxidation)	1					2.55	a							
Q9P2E9	Ribosome-binding protein 1	cytoplasm; endoplasmic reticulum; membrane; nucleus; organelle lumen; ribosome	HMAAASAECQNYAK	C9(Carbamidomethyl)	1					-1.14	a							
P62877	RING-box protein 1	cytoplasm; cytosol; nucleus	AAAMDVDTPSGTNSGAGK	N-Term(Acetyl); M4(Oxidation)	1					1.65	a							
P35637	RNA-binding protein FUS	cytoplasm; nucleus; organelle lumen	CPNPTCENMNFSWR	C1(Carbamidomethyl); C6(Carbamidomethyl); M9(Oxidation)	1	1.89	a											
Q9Y265	RuvB-like 1	chromosome; cytoplasm; cytoskeleton; Golgi; membrane; nucleus; organelle lumen	EVYEGEVTELTPCETENPMGGYGK	C13(Carbamidomethyl); M19(Oxidation)	1					1.53	a							#
Q9P0V9	Septin-10	cytoplasm; cytoskeleton; nucleus	TTCMSSQGSDDEQIKR	C3(Carbamidomethyl)	1					-1.20	a							
P83111	Serine beta-lactamase-like protein LACTB, mitochondrial	cytoplasm; mitochondrion	IFHDLDMLTTVQEENEPVIYNR		1											-1.36	a	
O15270	Serine palmitoyltransferase 2	cytoplasm; endoplasmic reticulum; membrane; mitochondrion	MRPEPGGCCCR	C8(Carbamidomethyl); C9(Carbamidomethyl); C10(Carbamidomethyl)	1			-0.74	a									
			MRPEPGGCCCR	N-Term(Acetyl); C8(Carbamidomethyl); C9(Carbamidomethyl); C10(Carbamidomethyl)	1	-1.61	a											
Q13188	Serine/threonine-protein kinase 3	cytoplasm; nucleus	MEQPPAPK	N-Term(Acetyl); M1(Oxidation)	1	1.49	a			1.48	a							

P30153	Serine/threonine-protein phosphatase 2A 65 kDa regulatory subunit A alpha isoform	chromosome; cytoplasm; cytoskeleton; cytosol; membrane; mitochondrion; nucleus	VLAMSGDPNYLHR	M4(Oxidation)	1					2.52	a							
P36952	Serpin B5	cytoplasm; extracellular	MDALQLANSAFAVDLFK	N-Term(Acetyl); M1(Oxidation)	1	1.60	a			1.36	a							
P50454	Serpin H1	cytoplasm; endoplasmic reticulum; organelle lumen	LQIVEMPLAHK		1											-1.43	a	
			LSSLIILMPHHVEPLER		1	-1.14	a			-2.50	a	-1.16	a			-2.32	a	
P49591	Seryl-tRNA synthetase, cytoplasmic	cytoplasm; cytosol; mitochondrion	MVLDLDLFR		1									-1.06	a			
Q15005	Signal peptidase complex subunit 2	cytoplasm; endoplasmic reticulum; membrane	DPTGMDPDDIWQLSSSLKR		1											-1.31	a	
			FFDHSGTLVMDAYEPEISR		1					-1.44	a							
			KDPTGMDPDDIWQLSSSLK		1											-1.19	a	
P08240	Signal recognition particle receptor subunit alpha	cytoplasm; endoplasmic reticulum; membrane	GTLGGMFGMLK		2											-1.17	a	
P05023	Sodium/potassium-transporting ATPase subunit alpha-1	cytoplasm; membrane	DMTSEQLDDILK		1											-0.93	a	#
			MTVAHMWFDNQIHEADTTENQSGVSFDK		2					-1.35	a							#
P05026	Sodium/potassium-transporting ATPase subunit beta-1	membrane	DDMIFEDCGDVPSEPK	M3(Oxidation); C8(Carbamidomethyl)	1					3.44	a							
P54709	Sodium/potassium-transporting ATPase subunit beta-3	cytoplasm; membrane	NEDIPNVAVYPHNGMIDLK		1	-2.16	a			-2.56	a							
Q01082	Spectrin beta chain, brain 1	cytoplasm; cytoskeleton; cytosol; membrane; nucleus; organelle	EVVAGSHELGQDYEHVTMLQER		1	-1.25	a											
			TQTAIASEDMPNTLTEAEK	M10(Oxidation)	1					2.01	a							
Q13435	Splicing factor 3B subunit 2	nucleus; organelle lumen; spliceosomal complex	QEEMNSQQEEEEMETDAR	M4(Oxidation); M13(Oxidation)	2	2.65	a			1.46	a							
Q01130	Splicing factor, arginine/serine-rich 2	nucleus; organelle lumen; spliceosomal complex	DAEDAMDAMDGAVLDGR	M6(Oxidation); M9(Oxidation)	2	1.17	a											
P23246	Splicing factor, proline- and glutamine-rich	cytoplasm; nucleus; organelle lumen	MGGGGAMNMGDPYGSGGQK	M1(Oxidation); M7(Oxidation); M9(Oxidation)	3					2.17	a							
			MGGGGAMNMGDPYGSGGQK	M7(Oxidation); M9(Oxidation)	3	1.46	a			1.99	a							
Q14247	Src substrate cortactin	cytoplasm; cytoskeleton	GPVSGTEPEPVYSMEAADYR	M14(Oxidation)	1					1.99	a							#
Q9UJZ1	Stomatin-like protein 2	cytoplasm; cytoskeleton; membrane; mitochondrion;	DSNTILLPSNPGDVTSMVAQAMGVYGALTK	M17(Oxidation)	2					2.06	a							
P38646	Stress-70 protein, mitochondrial	cell surface; cytoplasm; mitochondrion; organelle lumen	EQQIVIQSSGGLSKDDIENMVK	M20(Oxidation)	1					1.88	a							#
			ETGVDLTKDNMALQR	M11(Oxidation)	1					2.96	a							#
			VEAVNMAEGIIHDTETK	M6(Oxidation)	1					1.54	a							#
Q8NBJ7	Sulfatase-modifying factor 2	cytoplasm; endoplasmic reticulum; organelle lumen	FLMGTNSPDSR	M3(Oxidation)	1					2.33	a							
O94901	SUN domain-containing protein 1	cytoplasm; membrane; nucleus	VDQMEGGAAGPSASVR	M4(Oxidation)	1	2.93	a	1.08	a	4.39	a							
O15260	Surfeit locus protein 4	cytoplasm; endoplasmic reticulum; Golgi; membrane	GQNDLMGTAEDFADQFLR		1					-1.27	a					-1.27	a	
			MWFQWSEQR	M1(Oxidation)	1					2.75	a							
			SMFAGVPTMR		2					-1.82	a					-1.82	a	
			SMFAGVPTMR	M2(Oxidation); M9(Oxidation)	2	2.83	a											
O43752	Syntaxin-6	cytoplasm; endosome; Golgi; membrane	IGGELEEQAVMLEDFSHELESTQSR		1					-2.61	a							
Q4KMP7	TBC1 domain family member 10B	cytoplasm	QFPFHEMFAAR	N-Term(Gln->pyro-Glu)	1					-2.09	a					-1.69	a	
Q9BYX2	TBC1 domain family member 2A	cytoplasm; membrane; nucleus	HLGTEIQNTMHNIR		1	-1.74	a											
P17987	T-complex protein 1 subunit alpha	chromosome; cytoplasm; cytoskeleton; cytosol; Golgi; membrane; nucleus; organelle	MEGPLSVFGDR	N-Term(Acetyl); M1(Oxidation)	1	1.74	a											#
P48643	T-complex protein 1 subunit epsilon	cytoplasm; cytoskeleton; cytosol; nucleus; organelle lumen	QQISLATQMVR	N-Term(Gln->pyro-Glu); M9(Oxidation)	1	1.29	a											#
P50990	T-complex protein 1 subunit theta	cytoplasm; cytoskeleton; cytosol; nucleus	APGFAQMLK	M7(Oxidation)	1	1.25	a			1.44	a							
P13726	Tissue factor	cell surface; cytoplasm; extracellular; membrane	STDSPVECMGQEK	C8(Carbamidomethyl)	1					-1.30	a	-0.93	a					
Q6P9B6	TLD domain-containing protein KIAA1609	membrane; lysosome	AKGPSENVSQEQFTASMSHLLK		1											-1.22	a	
			GPSENVSQEQFTASMSHLLK		1					-1.20	a							
P37802	Transgelin-2	cytoskeleton; membrane; nucleus	GASQAGMTGYGMPR	M7(Oxidation); M12(Oxidation)	2	2.19	a			2.93	a							#
			NVIGLQMGTNR	M7(Oxidation)	1	1.34	a			2.10	a							#
			QMEQISQFLQAAER	M2(Oxidation)	1	1.43	a			2.33	a							#
			QMEQISQFLQAAER	N-Term(Gln->pyro-Glu); M2(Oxidation)	1					2.40	a							#
P55072	Transitional endoplasmic reticulum ATPase	cytoplasm; cytosol; endoplasmic reticulum; membrane; nucleus; organelle lumen; proteasome	KYEMFAQTLQQSR	M4(Oxidation)	1					1.98	a							
P13693	Translationally-controlled tumor protein	cytoplasm; cytoskeleton; endosome; extracellular; nucleus; organelle lumen	VKPFMTGAAEQIK	M5(Oxidation)	1	1.50	a			1.89	a							
P43307	Translocon-associated protein subunit alpha	cytoplasm; endoplasmic reticulum; membrane	VEMGTSSQNDVDMSWIPQETLNQINK		2											-1.10	a	
Q92544	Transmembrane 9 superfamily member 4	membrane	EDMEEDQEHTYR		1					-2.11	a							
P49755	Transmembrane emp24 domain-containing protein 10	cytoplasm; endoplasmic reticulum; Golgi; membrane	IPDQLVILDMK	M10(Oxidation)	1	2.57	a			4.75	a							

Q9BVK6	Transmembrane emp24 domain-containing protein 9	cytoplasm; endoplasmic reticulum; Golgi; membrane	FSLFAGGMLR	M8(Oxidation)	1					2.58	a							
Q9P0I2	Transmembrane protein 111	cytoplasm; Golgi; membrane	MMQEQMTGAAMAMPADTNK	M1(Oxidation); M2(Oxidation); M6(Oxidation); M11(Oxidation); M13(Oxidation)	5					6.11	a							
P57088	Transmembrane protein 33	cytoplasm; membrane	ADTTPNGPQGAGAVQFMMTNK	N-Term(Acetyl)	2					-1.90	a							
Q8WWA1	Transmembrane protein 40	membrane	METSASSSQPQDNSQVHR	N-Term(Acetyl); M1(Oxidation)	1					2.88	a							
P40939	Trifunctional enzyme subunit alpha	cytoplasm; membrane; mitochondrion; nucleus; organelle lumen	ALMGLYHGQVLCK	C12(Carbamidomethyl)	1					-1.31	a							#
			ELHSEFSEVMNEIWASDQIR		1											-1.21	a	#
			MVGVPAALDMMLTGR	M1(Oxidation)	3					1.69	a							#
			SAVLISSKPGCFIAGADINMLAACK	C11(Carbamidomethyl); M20(Oxidation); C24(Carbamidomethyl)	1					2.84	a							#
P55084	Trifunctional enzyme subunit beta, mitochondrial	cytoplasm; membrane; mitochondrion; organelle lumen	KLMLDLNK		1											-1.03	a	
Q9BQE3	Tubulin alpha-1C chain	cytoplasm; cytoskeleton	AVCMLSNTTAVAEAWAR	C3(Carbamidomethyl); M4(Oxidation)	1					1.30	a							#
P07437	Tubulin beta chain	cytoplasm; cytoskeleton; cytosol; membrane; nucleus	MAVTFIGNSTAIQELFK	M1(Oxidation)	1					1.94	a							
O43399	Tumor protein D54	cytoplasm	MDSAGQDINLNSPNK	N-Term(Acetyl); M1(Oxidation)	1	1.64	a											
P09758	Tumor-associated calcium signal transducer 2	cytoplasm; cytosol; membrane	MTVCSPDGPGGR	M1(Oxidation); C4(Carbamidomethyl)	1	3.18	a			4.53	a							
Q92890	Ubiquitin fusion degradation protein 1 homolog	cytoplasm; cytosol; nucleus	MFSFNMFDHPIPR	N-Term(Acetyl)	2							-1.10	a					
P62253	Ubiquitin-conjugating enzyme E2 G1	cytoplasm; nucleus; endoplasmic reticulum	MTELQSALLLR	N-Term(Acetyl)	1			-0.64	a									
Q9NYU2	UDP-glucose:glycoprotein glucosyltransferase 1	cytoplasm; endoplasmic reticulum; organelle lumen	EQLDPDELETITMHK		1											-2.51	a	
Q9P1F3	UPF0727 protein C6orf115		MNVDHEVNLLVEEIHR		1									0.81	a			
O75436	Vacuolar protein sorting-associated protein 26A	cytoplasm; cytosol; endosome; membrane	SYDFEFMQVEKPYESYIGANVR	M7(Oxidation)	1					1.79	a							
Q8N1B4	Vacuolar protein sorting-associated protein 52 homolog	cytoplasm; endosome; Golgi; membrane	AAAATMAAAAR	N-Term(Acetyl); M6(Oxidation)	1					3.33	a							
Q9BV40	Vesicle-associated membrane protein 8	cytoplasm; endosome; Golgi; membrane; mitochondrion;	MEEASEGGGNDR	N-Term(Acetyl); M1(Oxidation)	1					2.55	a							
Q9P0L0	Vesicle-associated membrane protein-associated protein A	cytoplasm; cytoskeleton; endoplasmic reticulum; membrane	CVFEMPNENDKLNDMEPSK	C1(Carbamidomethyl); M15(Oxidation)	2					4.02	a							
O75396	Vesicle-trafficking protein SEC22b	cytoplasm; endoplasmic reticulum; Golgi; membrane	IMVANIEEVLQR	M2(Oxidation)	1	1.40	a											
			VLLTMIAR		1			0.38								-1.21	a	
Q12907	Vesicular integral-membrane protein VIP36	cytoplasm; endoplasmic reticulum; Golgi; membrane	LFQLMVEHTPDEESIDWTK		1					-2.17	a					-1.82	a	
			LTVMTDLEDKNEWK		1					-1.33	a					-1.44	a	
P18206	Vinculin	cytoplasm; cytoskeleton; cytosol; extracellular; membrane	TNISDEESEQATEMLVHNAQNLMQSVK	M14(Oxidation)	2					1.37	a							
P21796	Voltage-dependent anion-selective channel protein 1	cytoplasm; membrane; mitochondrion; organelle lumen	EHINLGCDMDFDIAGPSIR	C7(Carbamidomethyl)	1					-1.43	a					-1.33	a	
Q9H6Y2	WD repeat-containing protein 55	cytoplasm; nucleus; organelle	TCEERPAEDGSDEEDPDSMEAPTR	C2(Carbamidomethyl)	1					-1.73	a							

Table S3. Theoretical calculation of how oxidation during sample preparation could affect the observed ratios of oxidized and reduced Met-peptides between PDT and control samples

Met-peptide	No. of Met-peptide in control sample	No. of Met-peptide in PDT sample	Met-peptide ratio (PDT/Ctrl)
Reduced form	99	90	0.91
Oxidized form	1	10	10
If 10% oxidation of Met residue occurred during sample preparation			
Reduced form	99-99*10% = 89.1	90-90*10% = 81	0.91
Oxidized form	1+99*10% = 10.9	10+90*10% = 19	1.74

Theoretically, the ratio of reduced Met-peptides in PDT versus control samples should represent the percentage of modified Met-peptides, and should not be influenced by modifications (such as oxidation) that occur during sample preparation. In contrast, the fold change of oxidized Met-peptides between PDT and control samples can be significantly affected by oxidation occurring during sample preparation. For example, in the control sample, one Met-peptide is oxidized and the other 99 Met-peptides are reduced. After PDT, we observe 10 oxidized Met-peptides and 90 reduced Met-peptides. The (PDT/Ctrl) ratio of the oxidized Met-peptides is 10/1=10, and that of the reduced Met-peptides is 90/99=0.91. Given an oxidation rate of 10% during sample preparation, the numbers of oxidized Met-peptides become 1+99*10%=10.9 in the control sample and 10+90*10%=19 in the PDT sample, while those of the reduced Met-peptides become 99-99*10%=89.1 and 90-90*10%=81, respectively. In this scenario, the PDT/Ctrl ratio of the reduced Met-peptides remains the same (0.91) before and after sample preparation. However, the ratio of the oxidized Met-peptides is greatly decreased (from 10 to 1.74) by sample preparation. Thus, measuring the changes in reduced Met-peptides (i.e., the parent peptides) enables a precise evaluation of the proportion of PDT-induced change. In contrast, although we must make the same measurement of oxidized Met-peptides to confirm that this is the relevant posttranslational modification, the calculation will underestimate changes of Met-peptide oxidation in the presence of protocol-related oxidation.

Table S4. Theoretical calculation of the percentage of Met oxidation of target Met peptides before and after PDT

Met-peptide	Before PDT	After PDT	Met-peptide ratio (PDT/Ctrl) detected
The percentage of oxidized form in	X%	aX%	a (assuming a-fold change detected)
The percentage of reduced form in	(100-X)%	(100-aX)%	b (assuming b-fold change detected)

Since the (after PDT/before PDT) ratio of reduced Met-peptide b = (100-aX)/(100-X), we can calculate X as (1-b)*100/(a-b). For example, if a = 3 (i.e., a 3-fold increase in the oxidized form of a Met-peptide is detected after PDT) and b = 0.5 (a 2-fold decrease in the reduced form of that Met-peptide is detected after PDT), then X (the percentage of Met oxidation of the Met-peptide before PDT) can be deduced as (1-0.5)*100/(3-0.5) = 20. After PDT, the oxidized Met-peptide percentage increased to aX%= 3*20% = 60%. This assumes that no other post-translational modification occurs simultaneously on the target Met-peptide during PDT.

Table S5. The Met-peptide pairs that showed significant changes in oxidation status under all three PDT conditions.

The oxidation percentage of each Met-peptide was calculated using the equation described in Table S4. Both oxidized and reduced forms Met-peptides identified in non-enriched samples and exhibited a significant change in oxidation/reduction (beyond mean ± 2SD in both swapping experiments) were analyzed. Theoretically, the peptide ratios (PDT/Ctrl) of the oxidized and reduced Met-peptide should be >1 and < 1, respectively, and the oxidation % before or after PDT should be between 0 and 100. The Met-peptides that did not fit these criteria are marked in gray. The red/bolded values in the "Oxidation % Difference" column represent Met-peptides that showed >20% oxidation changes after PDT.

					Peptide ratio (PDT/Ctrl)						Oxidation %							Protein ratio (PDT/Ctrl)		
					Oxidized Met-peptide			Reduced Met-peptide			Before PDT		After PDT		Difference					
Condition	Protein	Protein name	Sequence	Modification	Exp. 1	Exp. 2	Mean	Exp. 1	Exp. 2	Mean	Exp. 1	Exp. 2	Exp. 1	Exp. 2	Exp. 1	Exp. 2	Mean	Exp. 1	Exp. 2	Mean
I	P62258	14-3-3 protein epsilon	DNLTLWTSDMQGDGEEQNK	M10(Oxidation)	3.78	1.72	2.55	0.70	0.99	0.83	9.90	0.80	37.38	1.38	**27.48**	0.58	14.03	1.21	1.39	1.30
I	P62258	14-3-3 protein epsilon	MDDREDLVYQAK	N-Term(Acetyl); M1(Oxidation)	2.85	1.88	2.32	0.77	0.96	0.86	10.84	4.07	30.91	7.66	**20.07**	3.59	11.83	1.21	1.39	1.30
I	P62081	40S ribosomal protein S7	IVKPNGEKPDEFESGISQALLELEMNSDLK	M25(Oxidation)	3.78	4.12	3.94	0.57	0.94	0.73	13.27	1.92	50.14	7.90	**36.86**	5.98	**21.42**	1.17	1.02	1.09
I	P26373	60S ribosomal protein L13	LATQLTGPVMPVR	M10(Oxidation)	0.88	0.95	0.91	0.29	0.54	0.39	119.52	113.30	105.62	107.19	-13.89	-6.11	-10.00	0.89	1.01	0.95
I	P11021	78 kDa glucose-regulated protein	TFAPEEISAMVLTK	M10(Oxidation)	5.34	2.11	3.35	0.65	0.93	0.78	7.46	6.30	39.82	13.26	**32.36**	6.96	19.66	0.80	0.89	0.84
I	Q01518	Adenylyl cyclase-associated protein 1	ADMQNLVER	N-Term(Acetyl); M3(Oxidation)	5.13	2.21	3.37	0.36	0.95	0.59	13.35	4.05	68.54	8.96	**55.19**	4.91	**30.05**	0.78	1.03	0.89
I	P12814	Alpha-actinin-1	VLAVNQENEQLMEDYEK	M12(Oxidation)	4.42	2.20	3.12	0.67	1.03	0.83	8.76	-2.20	38.77	-4.83	**30.01**	-2.63	13.69	0.73	0.73	0.73
I	P12814	Alpha-actinin-1	ETADTDTADQVMASFK	M12(Oxidation)	3.10	1.80	2.37	0.69	0.86	0.77	12.81	15.11	39.71	27.27	**26.90**	12.16	19.53	0.73	0.73	0.73
I	P12814	Alpha-actinin-1	GISQEQMNEFR	M7(Oxidation)	2.91	1.67	2.21	0.58	0.92	0.73	18.03	10.73	52.41	17.97	**34.39**	7.24	**20.81**	0.73	0.73	0.73
I	O43707	Alpha-actinin-4	GISQEQMQEFR	M7(Oxidation)	3.34	3.45	3.40	0.70	1.03	0.85	11.27	-1.42	37.70	-4.90	**26.43**	-3.48	11.48	0.95	0.91	0.93
I	P61163	Alpha-centractin	MESYDVIANQPVVIDNGSGVIK	N-Term(Acetyl); M1(Oxidation)	5.24	1.89	3.15	0.89	1.02	0.95	2.54	-2.52	13.31	-4.76	10.77	-2.24	4.26	1.08	1.25	1.16
I	P04083	Annexin A1	AMVSEFLK	N-Term(Acetyl); M2(Oxidation)	7.09	3.28	4.82	0.59	0.91	0.73	6.37	3.70	45.19	12.14	**38.82**	8.43	**23.62**	0.52	0.72	0.61
I	P04083	Annexin A1	MYGISLCQAILDETK	M1(Oxidation); C7(Carbamidomethyl)	7.35	2.92	4.63	0.49	0.74	0.60	7.46	12.00	54.79	35.06	**47.33**	**23.06**	**35.20**	0.52	0.72	0.61
I	P00966	Argininosuccinate synthase	MPEFYNR	M1(Oxidation)	3.87	4.62	4.23	0.56	0.89	0.71	13.26	2.92	51.24	13.50	**37.98**	10.58	**24.28**	1.25	1.83	1.51
I	Q6NUK1	Calcium-binding mitochondrial carrier protein SCaMC-1	MNIFGGFR	M1(Oxidation)	3.02	4.08	3.51	0.45	0.86	0.62	21.34	4.31	64.51	17.58	**43.17**	13.28	**28.22**	0.95	1.06	1.00
I	Q15417	Calponin-3	GMSVYGLGR	M2(Oxidation)	1.08	1.56	1.30	0.29	0.58	0.41	89.52	42.44	96.94	66.33	7.41	**23.89**	15.65	1.17	1.02	1.09
I	P20645	Cation-dependent mannose-6-phosphate receptor	GMEQFPHLAFWQDLGNLVADGCDFVCR	M2(Oxidation); C22(Carbamidomethyl); C26(Carbamidomethyl)	4.92	4.53	4.72	0.36	0.80	0.54	13.99	5.32	68.86	24.12	**54.88**	18.80	**36.84**	1.31	1.28	1.30
I	P16070	CD44 antigen	ESSETPDQFMTADETR	M10(Oxidation)	3.18	2.92	3.05	0.23	0.85	0.44	26.16	7.06	83.27	20.59	**57.11**	13.53	**35.32**	1.35	0.78	1.03
I	O75131	Copine-3	VECYDYDNDGSHDLIGTFQTTMTK	C3(Carbamidomethyl); M22(Oxidation)	3.12	2.04	2.52	0.78	1.04	0.90	9.45	-4.27	29.48	-8.71	**20.03**	-4.45	7.79	0.87	1.03	0.94
I	P22695	Cytochrome b-c1 complex subunit 2, mitochondrial	QVAEQFLNMR	N-Term(Gln->pyro-Glu); M9(Oxidation)	15.23	2.60	6.29	0.87	1.04	0.95	0.88	-2.46	13.34	-6.41	12.46	-3.95	4.26	0.96	0.99	0.98
I	P15924	Desmoplakin	QMGQPCDAYQK	N-Term(Gln->pyro-Glu); M2(Oxidation); C6(Carbamidomethyl)	0.75	0.82	0.79	0.11	0.61	0.26	138.35	183.97	104.36	151.54	-33.99	-32.43	-33.21	1.05	0.77	0.90
I	P31689	DnaJ homolog subfamily A member 1	EVEETDEMDQVELVDFDPNQER	M8(Oxidation)	1.65	0.88	1.20	0.32	0.46	0.38	51.25	128.31	84.34	113.02	**33.08**	-15.29	8.90	1.01	0.88	0.94
I	Q9H4M9	EH domain-containing protein 1	GGAFDGTMNGPFGHGYGEGAGEGIDDVEWVVGK	M8(Oxidation)	14.21	12.13	13.13	0.56	1.12	0.79	3.25	-1.11	46.15	-13.52	**42.90**	-12.40	15.25	0.97	0.89	0.93
I	P26641	Elongation factor 1-gamma	AAAPAPEEEMDECEQALAAEPK	M10(Oxidation); C13(Carbamidomethyl)	5.24	2.15	3.36	0.63	0.84	0.73	8.00	12.52	41.89	26.90	**33.89**	14.39	**24.14**	0.90	0.98	0.94
I	P13639	Elongation factor 2	QFAEMYVAK	M5(Oxidation)	4.31	1.83	2.80	0.89	0.95	0.92	3.35	6.11	14.43	11.15	11.08	5.04	8.06	0.85	0.87	0.86
I	Q9BS26	Endoplasmic reticulum resident protein 44	EITFENGEELTEEGLPFLILFHMK	M23(Oxidation)	4.58	2.15	3.14	0.85	1.02	0.93	4.10	-1.49	18.80	-3.21	14.70	-1.72	6.49	1.02	0.85	0.93
I	P00533	Epidermal growth factor receptor	WMALESILHR	M2(Oxidation)	7.72	10.58	9.04	0.41	0.76	0.56	8.05	2.45	62.15	25.90	**54.10**	**23.45**	**38.77**	1.03	0.78	0.90
I	P00533	Epidermal growth factor receptor	GNMYYENSYALAVLSNYDANK	M3(Oxidation)	3.44	2.10	2.69	0.73	1.00	0.85	10.07	0.22	34.66	0.46	**24.59**	0.24	12.42	1.03	0.78	0.90
I	P78344	Eukaryotic translation initiation factor 4 gamma 2	MESAIAEGGASR	N-Term(Acetyl); M1(Oxidation)	12.56	22.67	16.87	0.46	0.59	0.52	4.49	1.86	56.44	42.16	**51.94**	**40.30**	**46.12**	0.90	0.96	0.93
I	P55010	Eukaryotic translation initiation factor 5	AMGPLVLTEVLFNEK	M2(Oxidation)	4.06	2.17	2.97	0.70	0.87	0.78	8.89	10.21	36.11	22.13	**27.23**	11.92	19.57	0.84	0.96	0.90
I	P01111	GTPase NRas	QVVIDGETCLLDILDTAGQEEYSAMR	C9(Carbamidomethyl); M25(Oxidation)	3.44	2.34	2.84	0.57	1.15	0.81	14.87	-12.35	51.19	-28.85	**36.32**	-16.50	9.91	1.02	0.97	1.00
I	P11142	Heat shock cognate 71 kDa protein	NQVAMNPTNTVFDAK	M5(Oxidation)	3.94	1.85	2.70	0.79	1.05	0.91	6.72	-5.85	26.49	-10.82	19.77	-4.97	7.40	1.01	1.08	1.04
I	P07900	Heat shock protein HSP 90-alpha	YYTSASGDEMVSLK	M10(Oxidation)	3.74	1.87	2.65	0.46	0.90	0.64	16.56	10.73	62.01	20.08	**45.45**	9.35	**27.40**	1.27	1.04	1.15
I	P08238	Heat shock protein HSP 90-beta	VFIMDSCDELIPEYLNFIR	M4(Oxidation); C7(Carbamidomethyl)	4.05	1.96	2.82	0.56	0.87	0.70	12.59	12.00	50.99	23.56	**38.40**	11.56	**24.98**	1.15	1.00	1.07
I	Q00839	Heterogeneous nuclear ribonucleoprotein U	MCLFAGFQR	M1(Oxidation); C2(Carbamidomethyl)	3.82	2.33	2.98	0.61	0.90	0.74	12.26	7.04	46.78	16.39	**34.52**	9.35	**21.93**	0.99	1.11	1.05
I	P22626	Heterogeneous nuclear ribonucleoproteins A2/B1	NMGGPYGGGNYGPGGSGGSGGYGGR	M2(Oxidation)	1.31	0.62	0.90	0.34	0.59	0.45	68.37	1437	89.31	884.93	**20.94**	-552.35	-265.70	0.92	1.07	0.99
I	P62805	Histone H4	TVTAMDVVYALK	M5(Oxidation)	2.87	2.09	2.45	0.65	1.03	0.81	15.89	-2.37	45.68	-4.96	**29.79**	-2.59	13.60	0.83	1.01	0.91
I	Q16543	Hsp90 co-chaperone Cdc37	QYMEGFNDELEAFK	N-Term(Gln->pyro-Glu); M3(Oxidation)	8.19	1.69	3.72	0.84	0.93	0.88	2.22	9.15	18.16	15.45	15.94	6.30	11.12	0.77	0.96	0.86
I	Q9Y624	Junctional adhesion molecule A	EDTGTYTCMVSEEGGNSYGEVK	C8(Carbamidomethyl); M9(Oxidation)	3.81	4.18	3.99	0.52	0.90	0.69	14.48	3.01	55.21	12.57	**40.73**	9.56	**25.15**	1.20	1.43	1.31
I	P30740	Leukocyte elastase inhibitor	MEQLSSANTR	N-Term(Acetyl); M1(Oxidation)	3.77	1.70	2.53	0.42	1.00	0.65	17.36	0.03	65.51	0.06	**48.15**	0.02	**24.09**	0.84	0.90	0.87
I	Q9Y5L4	Mitochondrial import inner membrane translocase subunit Tim13	MEGGFGSDFGGSGSGK	N-Term(Acetyl); M1(Oxidation)	3.49	1.59	2.36	0.49	0.98	0.70	16.87	3.23	58.92	5.14	**42.05**	1.91	**21.98**	0.67	0.73	0.70
I	Q9NS69	Mitochondrial import receptor subunit TOM22 homolog	LQMEQQQQLQQR	M3(Oxidation)	6.90	1.95	3.67	0.61	1.05	0.80	6.21	-6.02	42.85	-11.75	**36.64**	-5.72	15.46	0.93	0.97	0.95
I	P28482	Mitogen-activated protein kinase 1	AAAAAAGAGPEMVR	N-Term(Acetyl); M12(Oxidation)	2.89	2.20	2.52	0.86	0.96	0.91	6.80	3.11	19.65	6.84	12.85	3.73	8.29	1.37	0.93	1.13
I	P26038	Moesin	GMLREDAVLEYLK	M2(Oxidation)	3.07	1.66	2.26	0.97	1.13	1.05	1.55	-25.26	4.76	-41.93	3.21	-16.68	-6.73	0.66	0.75	0.70
I	P26038	Moesin	QRIDEFESM	M9(Oxidation)	9.79	3.39	5.76	0.78	1.05	0.91	2.42	-2.08	23.66	-7.04	**21.24**	-4.97	8.14	0.66	0.75	0.70
I	Q9NZM1	Myoferlin	DIVIEMEDTKPLLASK	M6(Oxidation)	5.31	2.59	3.71	0.84	1.03	0.93	3.50	-2.00	18.56	-5.18	15.06	-3.18	5.94	1.58	1.56	1.57
I	O95169	NADH dehydrogenase [ubiquinone] 1 beta subcomplex subunit 8	VEDYEPYPDDGMGYGDYPK	M12(Oxidation)	4.05	1.79	2.69	0.73	0.96	0.84	8.17	4.84	33.09	8.68	**24.92**	3.84	14.38	0.84	1.25	1.02
I	Q09666	Neuroblast differentiation-associated protein AHNAK	VEAPSLDVHMDSPDINIEGPDVK	M10(Oxidation)	3.54	1.60	2.38	0.64	0.99	0.80	12.26	1.80	43.41	2.87	**31.15**	1.07	16.11	1.01	1.02	1.02
I	Q96TA1	Niban-like protein 1	NHVQPYIPSILEALMVPTSQGFTEVR	M15(Oxidation)	0.92	1.44	1.15	0.24	0.59	0.38	111.13	48.21	102.69	69.31	-8.44	**21.10**	6.33	1.10	0.88	0.99
I	Q96RS6	NudC domain-containing protein 1	MEVAANCSLR	N-Term(Acetyl); M1(Oxidation); C7(Carbamidomethyl)	5.18	3.13	4.03	0.46	0.92	0.65	11.44	3.71	59.23	11.61	**47.79**	7.90	**27.84**	0.93	0.79	0.86
I	Q9BRP8	Partner of Y14 and mago	MEAAGSPAATETGK	N-Term(Acetyl); M1(Oxidation)	3.07	1.63	2.24	0.88	0.95	0.91	5.31	7.80	16.34	12.72	11.02	4.91	7.97	0.77	0.96	0.86
I	Q02790	Peptidyl-prolyl cis-trans isomerase FKBP4	ATESGAQSAPLPMEGVDISPK	M13(Oxidation)	4.60	2.99	3.71	0.85	1.00	0.92	4.10	0.16	18.88	0.48	14.78	0.32	7.55	1.02	1.09	1.05
I	O95394	Phosphoacetylglucosamine mutase	MGLLAVLR	M1(Oxidation)	4.97	2.83	3.75	0.60	0.96	0.76	9.21	2.25	45.77	6.36	**36.56**	4.11	**20.34**	1.47	1.19	1.32
I	Q8NC51	Plasminogen activator inhibitor 1 RNA-binding protein	SKSEEAHAEDSVMDHHFR	M13(Oxidation)	3.84	2.38	3.02	0.94	1.09	1.01	1.98	-7.01	7.61	-16.68	5.63	-9.67	-2.02	0.81	0.92	0.86
I	O14744	Protein arginine N-methyltransferase 5	AAMAVGGAGGSR	N-Term(Acetyl); M3(Oxidation)	3.17	1.73	2.34	0.86	1.02	0.93	6.16	-2.54	19.52	-4.40	13.37	-1.86	5.75	1.08	0.99	1.03

I	Q13158	Protein FADD	SCQMNLVADLVQEVQQAR	C2(Carbamidomethyl); M4(Oxidation)	4.74	1.65	2.80	0.62	0.85	0.72	9.26	19.05	43.88	31.50	34.62	12.45	23.54	1.48	0.91	1.16
I	Q96A26	Protein FAM162A	EDEIPETVSLEMLDAAK	M12(Oxidation)	14.95	1.74	5.10	0.57	1.02	0.76	2.99	-2.49	44.69	-4.33	41.70	-1.84	19.93	1.46	1.03	1.23
I	Q92734	Protein TFG	MNGQLDLSGK	N-Term(Acetyl); M1(Oxidation)	8.59	1.97	4.12	1.13	1.10	1.11	-1.68	-11.15	-14.43	-21.98	-12.75	-10.84	-11.79	0.74	0.90	0.82
I	P35637	RNA-binding protein FUS	CPNPTCENMNFSWR	C1(Carbamidomethyl); C6(Carbamidomethyl); M9(Oxidation)	4.02	2.17	2.95	0.74	1.01	0.87	7.93	-0.99	31.85	-2.15	23.92	-1.16	11.38	1.21	1.02	1.11
I	Q13188	Serine/threonine-protein kinase 3	MEQPPAPK	N-Term(Acetyl); M1(Oxidation)	3.40	1.92	2.56	0.49	0.95	0.68	17.42	5.57	59.18	10.71	41.75	5.14	23.45	0.86	0.96	0.91
I	P36952	Serpin B5	MDALQLANSAFAVDLFK	N-Term(Acetyl); M1(Oxidation)	2.93	7.41	4.66	0.42	0.86	0.60	23.24	2.21	68.05	16.33	44.81	14.13	29.47	0.94	0.90	0.92
I	P17987	T-complex protein 1 subunit alpha	MEGPLSVFGDR	N-Term(Acetyl); M1(Oxidation)	3.62	1.74	2.51	0.44	0.94	0.64	17.60	7.11	63.77	12.39	46.17	5.27	25.72	1.15	1.22	1.18
I	P48643	T-complex protein 1 subunit epsilon	QQISLATQMVR	N-Term(Gln->pyro-Glu); M9(Oxidation)	5.12	1.59	2.86	0.88	1.00	0.94	2.73	-0.11	13.98	-0.17	11.25	-0.06	5.60	1.15	1.19	1.17
I	P50990	T-complex protein 1 subunit theta	APGFAQMLK	M7(Oxidation)	7.14	4.11	5.42	0.79	0.97	0.88	3.24	1.00	23.13	4.11	19.89	3.11	11.50	1.28	1.31	1.29
I	P37802	Transgelin-2	NVIGLQMGTNR	M7(Oxidation)	3.59	1.95	2.64	0.67	1.09	0.85	11.40	-10.48	40.92	-20.40	29.53	-9.91	9.81	1.17	1.49	1.32
I	P37802	Transgelin-2	QMEQISQFLQAAER	M2(Oxidation)	14.72	2.04	5.49	1.83	0.94	1.31	-6.45	5.65	-94.98	11.55	-88.52	5.90	-41.31	1.17	1.49	1.32
I	P13693	Translationally-controlled tumor protein	VKPFMTGAAEQIK	M5(Oxidation)	8.00	4.43	5.95	1.07	1.05	1.06	-1.04	-1.62	-8.34	-7.17	-7.30	-5.55	-6.42	0.97	1.23	1.09
I	P49755	Transmembrane emp24 domain-containing protein 10	IPDQLVILDMK	M10(Oxidation)	3.77	1.94	2.70	1.02	1.07	1.04	-0.62	-7.96	-2.32	-15.45	-1.70	-7.48	-4.59	1.70	0.64	1.04
I	O43399	Tumor protein D54	MDSAGQDINLNSPNK	N-Term(Acetyl); M1(Oxidation)	5.26	2.04	3.28	0.82	0.98	0.90	4.04	2.02	21.28	4.12	17.23	2.10	9.67	0.92	1.14	1.02
I	P09758	Tumor-associated calcium signal transducer 2	MTVCSPDGPGGR	M1(Oxidation); C4(Carbamidomethyl)	3.48	3.46	3.47	0.54	0.80	0.66	15.78	7.37	54.84	25.53	39.06	18.16	28.61	0.87	0.32	0.52
I	O75396	Vesicle-trafficking protein SEC22b	IMVANIEEVLQR	M2(Oxidation)	4.44	2.31	3.21	0.71	0.95	0.83	7.67	3.33	34.05	7.71	26.38	4.38	15.38	0.75	0.98	0.86
II	P07339	Cathepsin D	DPDAQPGGELMLGGTDSK	M11(Oxidation)	0.34	0.71	0.49	0.46	0.57	0.51	-445.35	305.84	-150.43	217.47	294.92	-88.37	103.28	1.91	1.10	1.45
II	P07339	Cathepsin D	ISVNNVLPVFDNLMQQK	M14(Oxidation)	1.47	1.57	1.52	1.01	0.80	0.90	-3.08	25.79	-4.51	40.58	-1.43	14.79	6.68	1.91	1.10	1.45
II	Q9NYP7	Elongation of very long chain fatty acids protein 5	MEHFDASLSTYFK	N-Term(Acetyl); M1(Oxidation)	1.50	1.70	1.60	0.74	1.16	0.93	34.11	-28.86	51.19	-49.03	17.08	-20.16	-1.54	0.92	1.12	1.02
II	P61916	Epididymal secretory protein E1	AVVHGILMGVPVPFPIPEPDGCK	M8(Oxidation); C22(Carbamidomethyl)	2.52	2.61	2.56	0.73	0.71	0.72	15.21	15.02	38.27	39.26	23.07	24.24	23.65	1.04	0.87	0.95
II	P38159	Heterogeneous nuclear ribonucleoprotein G	MVEADRPGK	N-Term(Acetyl); M1(Oxidation)	5.28	5.06	5.16	1.00	0.77	0.88	0.00	5.27	0.02	26.64	0.01	21.37	10.69	0.86	1.05	0.95
II	O96008	Mitochondrial import receptor subunit TOM40 homolog	RPGEEGTVMSLAGK	M9(Oxidation)	0.64	0.67	0.65	0.53	0.69	0.60	430.80	-1547	274.10	-1034	-156.70	513.08	178.19	0.91	0.91	0.91
II	Q16891	Mitochondrial inner membrane protein	LFEMVLGPAAYNVPLPK	M4(Oxidation)	2.59	2.46	2.52	1.04	1.05	1.05	-2.60	-3.83	-6.72	-9.44	-4.12	-5.61	-4.86	1.05	0.78	0.91
II	P62937	Peptidyl-prolyl cis-trans isomerase A	MVNPTVFFDIAVDGEPLGR	N-Term(Acetyl); M1(Oxidation)	1.08	1.54	1.29	0.66	0.66	0.66	81.40	38.93	87.75	59.99	6.35	21.06	13.70	1.20	1.22	1.21
III	P62258	14-3-3 protein epsilon	DNLTLWTSDMQGDGEEQNK	M10(Oxidation)	8.53	2.38	4.50	0.72	0.67	0.69	3.61	19.45	30.80	46.19	27.18	26.74	26.96	1.32	1.40	1.36
III	P62258	14-3-3 protein epsilon	DNLTLWTSDMQGDGEEQNKEALQDVEDENQ	M10(Oxidation)	18.67	5.23	9.89	0.38	0.31	0.34	3.41	14.08	63.70	73.69	60.29	59.61	59.95	1.32	1.40	1.36
III	P31947	14-3-3 protein sigma	SAYQEAMDISKK	M7(Oxidation)	6.71	4.91	5.74	0.61	0.57	0.59	6.42	9.83	43.09	48.23	36.67	38.40	37.54	0.79	0.91	0.85
III	P31947	14-3-3 protein sigma	TTFDEAMADLHTLSEDSYK	M7(Oxidation)	2.93	2.51	2.71	0.87	1.00	0.93	6.47	0.15	18.93	0.37	12.46	0.22	6.34	0.79	0.91	0.85
III	P43686	26S protease regulatory subunit 6B	MEEIGILVEK	N-Term(Acetyl); M1(Oxidation)	5.12	3.80	4.41	0.95	0.86	0.91	1.12	4.74	5.75	18.02	4.62	13.27	8.95	0.89	1.03	0.95
III	P62195	26S protease regulatory subunit 8	ALDGPEQMELEEGK	N-Term(Acetyl); M8(Oxidation)	6.27	8.77	7.42	0.94	1.00	0.97	1.07	-0.01	6.69	-0.11	5.63	-0.10	2.76	0.94	1.05	0.99
III	O00487	26S proteasome non-ATPase regulatory subunit 14	AVEEEDKMTPEQLAIK	M8(Oxidation)	10.52	2.79	5.42	0.76	0.77	0.77	2.42	11.24	25.45	31.37	23.03	20.13	21.58	1.00	0.96	0.98
III	P25398	40S ribosomal protein S12	AEEGIAAGGVMDVNTALQEVLK	N-Term(Acetyl); M11(Oxidation)	6.82	7.36	7.08	0.77	0.74	0.76	3.79	3.89	25.88	28.64	22.09	24.75	23.42	0.97	1.10	1.03
III	P62263	40S ribosomal protein S14	DESSPYAAMLAAQDVAQR	M9(Oxidation)	2.17	3.53	2.77	1.07	0.96	1.02	-6.78	1.37	-14.74	4.83	-7.96	3.46	-2.25	0.94	1.17	1.05
III	P08708	40S ribosomal protein S17	LLDFGSLSNLQVTQPTVGMNFK	M19(Oxidation)	2.14	2.73	2.42	0.90	1.20	1.04	7.85	-13.40	16.82	-36.55	8.97	-23.16	-7.09	0.98	1.11	1.04
III	P26373	60S ribosomal protein L13	LATQLTGPVMPVR	M10(Oxidation)	2.13	3.37	2.68	1.03	0.87	0.95	-3.17	5.16	-6.73	17.39	-3.56	12.23	4.33	0.90	1.15	1.02
III	O96019	Actin-like protein 6A	DDGSTLMEIDGDKGK	M7(Oxidation)	9.89	2.26	4.73	0.49	0.39	0.44	5.42	32.48	53.56	73.40	48.15	40.93	44.54	1.03	1.26	1.14
III	P07108	Acyl-CoA-binding protein	QATVGDINTERPGMLDFTGK	M14(Oxidation)	2.25	4.76	3.27	1.11	1.07	1.09	-9.61	-1.77	-21.63	-8.41	-12.02	-6.64	-9.33	1.19	1.22	1.21
III	Q01518	Adenylyl cyclase-associated protein 1	ADMQNLVER	N-Term(Acetyl); M3(Oxidation)	4.02	2.68	3.29	0.91	0.78	0.84	2.95	11.69	11.89	31.38	8.93	19.69	14.31	0.81	0.89	0.85
III	P05141	ADP/ATP translocase 2	MTDAAVSFAK	N-Term(Acetyl); M1(Oxidation)	6.25	5.12	5.66	0.85	0.75	0.80	2.74	5.68	17.13	29.09	14.39	23.41	18.90	0.78	0.93	0.85
III	P12236	ADP/ATP translocase 3	MTEQAISFAK	N-Term(Acetyl); M1(Oxidation)	3.89	3.88	3.89	1.21	1.28	1.24	-7.93	-10.63	-30.88	-41.29	-22.95	-30.66	-26.80	0.82	0.99	0.90
III	P12814	Alpha-actinin-1	ETADTDTADQVMASFK	M12(Oxidation)	4.82	4.90	4.86	0.92	0.72	0.81	2.00	6.77	9.63	33.17	7.64	26.40	17.02	0.66	0.67	0.66
III	P12814	Alpha-actinin-1	GISQEQMNEFR	M7(Oxidation)	12.16	11.24	11.69	0.65	0.73	0.69	3.04	2.59	36.95	29.08	33.92	26.49	30.20	0.66	0.67	0.66
III	P12814	Alpha-actinin-1	MVSDINNAWGCLEQVEK	M1(Oxidation); C11(Carbamidomethyl)	9.72	4.64	6.72	0.54	0.50	0.52	5.06	12.02	49.21	55.85	44.14	43.83	43.99	0.66	0.67	0.66
III	O43707	Alpha-actinin-4	GISQEQMQEFR	M7(Oxidation)	8.73	13.33	10.79	0.61	0.72	0.66	4.85	2.25	42.32	29.96	37.47	27.71	32.59	0.98	0.81	0.89
III	P04083	Annexin A1	MYGISLCQAILDETK	M1(Oxidation); C7(Carbamidomethyl)	3.28	2.43	2.82	0.74	0.80	0.77	10.33	12.14	33.92	29.48	23.59	17.34	20.46	0.60	0.66	0.63
III	P04083	Annexin A1	MYGISLCQAILDETKGDYEK	M1(Oxidation); C7(Carbamidomethyl)	2.27	2.08	2.17	0.85	0.88	0.87	10.30	10.29	23.33	21.43	13.03	11.14	12.09	0.60	0.66	0.63
III	P08758	Annexin A5	NFATSLYSMIK	M9(Oxidation)	9.67	4.52	6.61	0.56	0.53	0.55	4.80	11.70	46.42	52.93	41.62	41.22	41.42	0.89	0.82	0.85
III	P06576	ATP synthase subunit beta, mitochondrial	TVLIMELINNVAK	M5(Oxidation)	5.33	3.45	4.29	1.04	0.76	0.89	-0.83	8.77	-4.42	30.22	-3.59	21.45	8.93	1.04	1.14	1.09
III	Q9H3K6	BolA-like protein 2	MELSAEYLR	N-Term(Acetyl); M1(Oxidation)	7.79	2.81	4.68	0.62	0.57	0.59	5.33	19.11	41.50	53.66	36.17	34.55	35.36	1.43	0.93	1.16
III	Q6NUK1	Calcium-binding mitochondrial carrier protein SCaMC-1	MNIFGGFR	M1(Oxidation)	4.20	11.72	7.01	0.55	0.47	0.51	12.45	4.70	52.25	55.12	39.80	50.42	45.11	1.00	1.12	1.06
III	P04632	Calpain small subunit 1	MFLVNSFLK	N-Term(Acetyl); M1(Oxidation)	2.85	2.27	2.54	0.84	0.73	0.78	7.79	17.52	22.22	39.75	14.43	22.23	18.33	1.10	1.04	1.07
III	Q15417	Calponin-3	GASQAGMLAPGTR	M7(Oxidation)	4.70	3.88	4.27	0.76	0.80	0.78	6.03	6.56	28.32	25.44	22.29	18.88	20.59	1.19	0.81	0.98
III	P10644	cAMP-dependent protein kinase type I-alpha regulatory subunit	MESGSTAASEEAR	N-Term(Acetyl); M1(Oxidation)	4.80	6.98	5.79	0.68	0.71	0.69	7.86	4.63	37.78	32.33	29.92	27.69	28.81	1.04	0.90	0.97
III	P35221	Catenin alpha-1	MSASQLEALCPQVINAALALAAKPQSK	M1(Oxidation); C10(Carbamidomethyl)	9.13	13.92	11.27	0.66	0.55	0.61	3.97	3.34	36.24	46.50	32.27	43.16	37.72	1.20	0.68	0.90
III	O60716	Catenin delta-1	QPELPEVIAMLGFR	M10(Oxidation)	4.43	3.13	3.72	1.18	1.16	1.17	-5.55	-8.18	-24.58	-25.60	-19.04	-17.42	-18.23	1.30	0.81	1.03
III	P07339	Cathepsin D	AIGAVPLIQGEYMIPCEK	M13(Oxidation); C16(Carbamidomethyl)	3.03	4.85	3.83	0.90	0.92	0.91	4.69	1.93	14.19	9.35	9.50	7.42	8.46	1.75	1.46	1.60
III	P07339	Cathepsin D	DPDAQPGGELMLGGTDSK	M11(Oxidation)	11.15	21.28	15.41	0.86	0.57	0.70	1.38	2.06	15.42	43.93	14.04	41.87	27.95	1.75	1.46	1.60
III	P16070	CD44 antigen	ESSETPDQFMTADETR	M10(Oxidation)	3.48	3.23	3.35	0.97	0.84	0.90	1.00	6.81	3.49	22.03	2.49	15.22	8.85	0.97	1.29	1.12
III	Q00610	Clathrin heavy chain 1	TLQIFNIEMK	M9(Oxidation)	3.69	2.29	2.91	0.62	0.56	0.59	12.50	25.31	46.09	57.92	33.60	32.61	33.10	1.19	1.09	1.14
III	P61923	Coatomer subunit zeta-1	MEALILEPSLYTVK	N-Term(Acetyl); M1(Oxidation)	12.22	11.68	11.95	0.58	0.68	0.63	3.64	2.92	44.53	34.08	40.88	31.16	36.02	0.87	1.00	0.93
III	O75534	Cold shock domain-containing protein E1	GPDNSMGFGAER	M6(Oxidation)	7.18	6.06	6.60	0.83	1.07	0.94	2.67	-1.31	19.16	-7.92	16.49	-6.61	4.94	1.07	0.93	1.00
III	O75131	Copine-3	VECYDYDNDGSHDLIGTFQTTMTK	C3(Carbamidomethyl); M22(Oxidation)	12.55	2.86	5.99	0.59	0.31	0.43	3.46	27.00	43.44	77.17	39.98	50.17	45.07	1.12	1.06	1.09
III	P22695	Cytochrome b-c1 complex subunit 2, mitochondrial	QVAEQFLNMR	M9(Oxidation)	3.22	3.05	3.13	0.87	0.93	0.90	5.54	3.28	17.82	10.01	12.29	6.72	9.51	0.98	1.03	1.00
III	O15160	DNA-directed RNA polymerases I and III subunit RPAC1	AASQAVEEMR	N-Term(Acetyl); M9(Oxidation)	4.98	2.85	3.77	0.91	0.94	0.93	2.26	2.99	11.25	8.55	9.00	5.55	7.27	0.73	0.80	0.76
III	P50570	Dynamin-2	AGVYPEKDQAENEDGAQENTFSMDPQLER	M23(Oxidation)	11.75	8.49	9.99	0.54	0.46	0.50	4.13	6.72	48.53	57.03	44.40	50.31	47.36	1.08	1.07	1.08
III	P49792	E3 SUMO-protein ligase RanBP2	APGTNVAMASNQAVR	M8(Oxidation)	7.23	6.02	6.60	0.63	0.61	0.62	5.63	7.12	40.70	42.90	35.07	35.78	35.42	0.97	1.02	1.00
III	Q9H223	EH domain-containing protein 4	FMCSQLPNQVLK	M2(Oxidation); C3(Carbamidomethyl)	3.65	7.18	5.12	0.84	0.87	0.85	5.72	2.12	20.86	15.22	15.14	13.10	14.12	0.72	0.80	0.76
III	P29692	Elongation factor 1-delta	FYEQMNGPVAGASR	M5(Oxidation)	8.24	8.38	8.31	0.45	0.55	0.50	7.01	5.79	57.77	48.55	50.76	42.76	46.76	0.83	0.98	0.90
III	P26641	Elongation factor 1-gamma	AAAPAPEEEMDECEQALAAEPK	M10(Oxidation); C13(Carbamidomethyl)	3.25	3.19	3.22	0.96	1.00	0.98	1.90	-0.17	6.18	-0.53	4.28	-0.36	1.96	0.82	0.99	0.90
III	P13639	Elongation factor 2	QFAEMYVAK	N-Term(Gln->pyro-Glu); M5(Oxidation)	15.80	6.29	9.97	0.74	0.80	0.77	1.72	3.64	27.17	22.89	25.45	19.25	22.35	0.70	0.84	0.77
III	P30040	Endoplasmic reticulum resident protein 29	LAENSASSDDLLVAEVGISDYGDKLNMELSEK	M27(Oxidation)	17.34	12.77	14.88	0.55	0.65	0.60	2.70	2.85	46.91	36.41	44.20	33.56	38.88	1.04	1.16	1.10

III	P14625	Endoplasmin	NLLHVTDTGVGMTR	M12(Oxidation)	4.29	3.96	4.12	0.93	0.94	0.93	2.20	1.97	9.45	7.83	7.25	5.85	6.55	0.87	1.18	1.01
III	P14625	Endoplasmin	LTESPCALVASQYGWSGNMER	C6(Carbamidomethyl); M19(Oxidation)	2.27	2.25	2.26	0.93	0.96	0.94	4.89	3.46	11.10	7.76	6.21	4.31	5.26	0.87	1.18	1.01
III	P14625	Endoplasmin	TETVEEPMEEEEAAK	M8(Oxidation)	4.32	3.12	3.67	0.86	1.00	0.93	4.04	-0.23	17.47	-0.73	13.42	-0.50	6.46	0.87	1.18	1.01
III	P14625	Endoplasmin	TETVEEPMEEEEAAKEEK	M8(Oxidation)	10.97	5.52	7.78	0.83	0.74	0.79	1.64	5.43	17.94	29.96	16.31	24.53	20.42	0.87	1.18	1.01
III	P00533	Epidermal growth factor receptor	WMALESILHR	M2(Oxidation)	2.78	2.55	2.66	0.84	1.02	0.92	8.39	-1.04	23.31	-2.65	14.92	-1.61	6.65	0.99	0.57	0.75
III	P00533	Epidermal growth factor receptor	ACGADSYEMEEDGVR	C2(Carbamidomethyl); M9(Oxidation)	3.34	2.99	3.16	0.90	0.90	0.90	4.21	4.71	14.06	14.10	9.85	9.38	9.61	0.99	0.57	0.75
III	P00533	Epidermal growth factor receptor	ACGADSYEMEEDGVRK	C2(Carbamidomethyl); M9(Oxidation)	2.95	2.20	2.55	0.96	0.86	0.91	2.04	10.67	6.04	23.42	4.00	12.76	8.38	0.99	0.57	0.75
III	P00533	Epidermal growth factor receptor	EILDEAYVMASVDNPHVCR	M9(Oxidation); C18(Carbamidomethyl)	2.28	2.97	2.60	0.96	0.95	0.96	2.86	2.34	6.52	6.94	3.66	4.61	4.13	0.99	0.57	0.75
III	P00533	Epidermal growth factor receptor	GNMYYENSYALAVLSNYDANK	M3(Oxidation)	3.70	2.24	2.88	0.88	0.89	0.88	4.29	8.32	15.87	18.66	11.59	10.34	10.96	0.99	0.57	0.75
III	P60842	Eukaryotic initiation factor 4A-I	LNSNTQVVLLSATMPSDVLEVTKK	M14(Oxidation)	3.77	4.19	3.97	0.93	0.94	0.93	2.63	1.88	9.93	7.88	7.30	6.00	6.65	0.66	0.91	0.77
III	P05198	Eukaryotic translation initiation factor 2 subunit 1	ENAEVDGDDDAEEMEAKAED	M14(Oxidation)	19.20	18.67	18.94	0.75	0.68	0.71	1.36	1.78	26.14	33.29	24.78	31.51	28.14	0.84	0.91	0.87
III	O15371	Eukaryotic translation initiation factor 3 subunit D	NMLQFNLQILPK	M2(Oxidation)	8.36	7.18	7.75	0.64	0.51	0.57	4.72	7.38	39.45	53.02	34.73	45.64	40.19	0.95	1.15	1.05
III	Q15056	Eukaryotic translation initiation factor 4H	GSNMDFREPTEEER	M4(Oxidation)	3.99	3.76	3.87	0.78	0.88	0.83	6.96	4.02	27.77	15.08	20.82	11.07	15.94	0.95	1.12	1.03
III	P55010	Eukaryotic translation initiation factor 5	AMGPLVLTEVLFNEK	M2(Oxidation)	4.70	4.52	4.61	0.79	0.90	0.84	5.42	2.80	25.43	12.66	20.01	9.86	14.94	0.91	1.01	0.96
III	P47756	F-actin-capping protein subunit beta	QMEKDETVSDCSPHIANIGR	M2(Oxidation); C11(Carbamidomethyl)	2.56	2.44	2.50	0.87	0.72	0.80	7.48	16.04	19.11	39.20	11.63	23.16	17.40	1.26	1.24	1.25
III	P49327	Fatty acid synthase	LGMLSPEGTCK	M3(Oxidation); C10(Carbamidomethyl)	5.17	5.69	5.43	1.17	0.92	1.03	-4.12	1.75	-21.31	9.94	-17.19	8.19	-4.50	1.96	2.57	2.25
III	O75955	Flotillin-1	SPPVMVAGGR	M5(Oxidation)	2.59	6.23	4.02	0.98	1.12	1.05	1.32	-2.40	3.43	-14.94	2.11	-12.54	-5.22	1.99	0.38	0.86
III	P14314	Glucosidase 2 subunit beta	SLEDQVEMLR	M8(Oxidation)	7.75	15.18	10.85	0.51	0.49	0.50	6.74	3.45	52.27	52.36	45.52	48.91	47.22	0.93	0.57	0.73
III	P28799	Granulins	CDMEVSCPDGYTCCR	C1(Carbamidomethyl); M3(Oxidation); C7(Carbamidomethyl); C13(Carbamidomethyl); C14(Carbamidomethyl)	3.98	4.41	4.19	1.27	1.15	1.21	-9.94	-4.54	-39.54	-20.02	-29.60	-15.48	-22.54	1.27	0.40	0.71
III	Q9HAV7	GrpE protein homolog 1, mitochondrial	NSGQNLEEDMGQSEQK	M10(Oxidation)	2.60	2.33	2.46	0.89	0.77	0.83	6.30	14.86	16.37	34.58	10.07	19.73	14.90	0.84	1.02	0.92
III	Q9HAV7	GrpE protein homolog 1, mitochondrial	NSGQNLEEDMGQSEQKADPPATEK	M10(Oxidation)	9.77	10.71	10.23	0.65	0.54	0.59	3.81	4.56	37.24	48.85	33.43	44.29	38.86	0.84	1.02	0.92
III	P63244	Guanine nucleotide-binding protein subunit beta-2-like 1	GHNGWVTQIATTPQFPDMILSASR	M18(Oxidation)	1.04	0.53	0.74	0.44	0.37	0.40	93.54	387.91	97.19	205.60	3.65	-182.31	-89.33	0.96	1.15	1.05
III	P34932	Heat shock 70 kDa protein 4	NAVEEYVYEMR	M10(Oxidation)	2.28	2.36	2.32	0.85	0.97	0.91	10.22	2.28	23.25	5.38	13.04	3.10	8.07	0.91	1.18	1.03
III	P11142	Heat shock cognate 71 kDa protein	NQVAMNPTNTVFDAK	M5(Oxidation)	4.06	6.07	4.97	0.91	0.81	0.86	2.83	3.55	11.49	21.57	8.66	18.02	13.34	0.96	1.24	1.09
III	P11142	Heat shock cognate 71 kDa protein	NSLESYAFNMK	M10(Oxidation)	7.07	2.55	4.24	0.52	0.54	0.53	7.29	23.07	51.52	58.78	44.23	35.71	39.97	0.96	1.24	1.09
III	P07900	Heat shock protein HSP 90-alpha	YYTSASGDEMVSLK	M10(Oxidation)	2.45	14.03	5.86	1.48	1.16	1.31	-50.01	-1.28	-122.51	-17.99	-72.50	-16.71	-44.60	1.16	1.41	1.28
III	P08238	Heat shock protein HSP 90-beta	LVSSPCCIVTSTYGWTANMER	C6(Carbamidomethyl); C7(Carbamidomethyl); M19(Oxidation)	25.73	10.92	16.76	0.46	0.41	0.44	2.14	5.58	55.15	60.86	53.00	55.29	54.15	0.95	1.32	1.12
III	P22626	Heterogeneous nuclear ribonucleoproteins A2/B1	NMGGPYGGGNYGPGGSGGSGGYGGR	M2(Oxidation)	9.36	4.76	6.67	1.02	0.99	1.00	-0.19	0.36	-1.81	1.72	-1.61	1.36	-0.13	1.06	1.19	1.12
III	Q9Y4L1	Hypoxia up-regulated protein 1	VAIVKPGVPMEIVLNK	M10(Oxidation)	4.69	2.51	3.43	0.77	0.77	0.77	5.99	13.44	28.06	33.70	22.07	20.26	21.16	0.70	0.94	0.81
III	Q9Y4L1	Hypoxia up-regulated protein 1	VAIVKPGVPMEIVLNK	M10(Oxidation)	5.98	4.22	5.03	0.84	0.79	0.81	3.08	6.24	18.42	26.34	15.34	20.11	17.72	0.70	0.94	0.81
III	O95373	Importin-7	MDPNTIIEALR	N-Term(Acetyl); M1(Oxidation)	4.48	0.97	2.08	0.39	0.38	0.38	14.97	105.67	66.98	102.13	52.01	-3.54	24.24	0.83	1.03	0.92
III	P14923	Junction plakoglobin	VAAGVLCELAQDKEAADAIDAEGASAPLMELLHSR	C7(Carbamidomethyl); M29(Oxidation)	2.69	2.61	2.65	0.97	1.01	0.99	1.66	-0.32	4.47	-0.83	2.81	-0.51	1.15	1.39	0.52	0.85
III	Q9Y624	Junctional adhesion molecule A	EDTGTYTCMVSEEGGNSYGEVK	C8(Carbamidomethyl); M9(Oxidation)	2.53	3.45	2.95	0.88	0.87	0.87	7.49	4.96	18.91	17.14	11.42	12.18	11.80	1.70	1.00	1.30
III	P02545	Lamin-A/C	LQTMKEELDFQK	M4(Oxidation)	4.98	3.81	4.36	0.84	0.83	0.83	3.96	5.65	19.71	21.55	15.75	15.89	15.82	1.73	1.52	1.62
III	Q13751	Laminin subunit beta-3	AGGAFLMAGQVAEQLR	M7(Oxidation)	10.25	5.40	7.44	0.61	0.44	0.52	4.09	11.32	41.91	61.10	37.82	49.77	43.80	0.33	1.91	0.79
III	Q9NS86	LanC-like protein 2	VDQETLTEMVKPSIDYVR	M9(Oxidation)	5.16	3.03	3.96	0.91	0.98	0.94	2.18	1.00	11.27	3.03	9.09	2.03	5.56	0.94	0.95	0.94
III	P07195	L-lactate dehydrogenase B chain	GMYGIENEVFLSLPCILNAR	M2(Oxidation); C15(Carbamidomethyl)	10.04	6.99	8.38	0.55	0.54	0.54	4.74	7.14	47.65	49.91	42.90	42.77	42.84	0.84	1.12	0.97
III	P40926	Malate dehydrogenase, mitochondrial	FVFSLVDAMNGK	M9(Oxidation)	0.56	0.40	0.47	0.40	0.40	0.40	377.31	-213114	210.85	-85186	-166.46	127928	63881	1.18	1.23	1.21
III	O15173	Membrane-associated progesterone receptor component 2	DEYDDLSDLNAVQMESVR	M14(Oxidation)	2.36	3.16	2.73	0.80	0.95	0.87	12.63	2.43	29.83	7.69	17.20	5.25	11.23	0.74	0.74	0.74
III	P02795	Metallothionein-2	MDPNCSCAAGDSCTCAGSCK	M1(Oxidation); N-Term(Acetyl); C5(Carbamidomethyl); C7(Carbamidomethyl); C13(Carbamidomethyl); C15(Carbamidomethyl); C19(Carbamidomethyl)	3.35	2.85	3.09	0.96	0.97	0.96	1.59	1.74	5.34	4.96	3.74	3.22	3.48	0.45	0.47	0.46
III	Q8TCT9	Minor histocompatibility antigen H13	GEVTEMFSYEESNPKDPAAVTESK	M6(Oxidation)	6.26	7.27	6.75	0.74	0.70	0.72	4.77	4.58	29.86	33.29	25.09	28.71	26.90	0.98	1.56	1.24
III	Q9Y5L4	Mitochondrial import inner membrane translocase subunit Tim13	MEGGFGSDFGGSGSGK	N-Term(Acetyl); M1(Oxidation)	5.57	2.31	3.58	0.76	0.77	0.77	4.91	15.08	27.34	34.79	22.43	19.71	21.07	0.55	0.71	0.62
III	Q9NS69	Mitochondrial import receptor subunit TOM22 homolog	LQMEQQQQLQQR	M3(Oxidation)	2.44	2.16	2.29	0.97	0.95	0.96	2.18	4.40	5.32	9.50	3.14	5.10	4.12	1.04	1.06	1.05
III	O94826	Mitochondrial import receptor subunit TOM70	QAYTGNNSSQIQAAMK	M15(Oxidation)	26.02	41.97	33.04	1.49	2.39	1.89	-2.00	-3.51	-51.99	-147.10	-49.99	-143.59	-96.79	0.85	0.98	0.91
III	P28482	Mitogen-activated protein kinase 1	AAAAAAGAGPEMVR	N-Term(Acetyl); M12(Oxidation)	2.43	3.20	2.79	0.96	1.02	0.99	2.92	-0.80	7.09	-2.56	4.17	-1.76	1.21	1.38	0.75	1.02
III	P26038	Moesin	GMLREDAVLEYLK	M2(Oxidation)	4.38	3.06	3.66	0.64	0.68	0.66	9.58	13.42	41.93	41.10	32.36	27.68	30.02	0.68	0.67	0.67
III	Q9NZM1	Myoferlin	DIVIEMEDTKPLLASK	M6(Oxidation)	10.72	8.29	9.43	0.76	0.83	0.79	2.43	2.31	26.09	19.18	23.66	16.86	20.26	2.05	1.64	1.83
III	P35579	Myosin-9	IIGLDQVAGMSETALPGAFK	M10(Oxidation)	2.71	2.41	2.55	0.72	0.57	0.64	13.86	23.41	37.60	56.32	23.74	32.91	28.32	0.70	0.65	0.67
III	P35579	Myosin-9	ALEQQVEEMKTQLEELEDELQATEDAK	M9(Oxidation)	4.79	4.82	4.81	1.02	0.95	0.98	-0.53	1.40	-2.56	6.76	-2.03	5.35	1.66	0.70	0.65	0.67
III	P35579	Myosin-9	KMEDSVGCLETAEEVKR	M2(Oxidation); C8(Carbamidomethyl)	9.28	50.27	21.60	0.42	1.10	0.68	6.57	-0.21	60.96	-10.67	54.40	-10.46	21.97	0.70	0.65	0.67
III	Q96RS6	NudC domain-containing protein 1	MEVAANCSLR	N-Term(Acetyl); M1(Oxidation); C7(Carbamidomethyl)	2.53	2.30	2.41	0.88	0.84	0.86	7.11	11.15	17.95	25.65	10.84	14.50	12.67	0.74	0.94	0.83
III	Q9BRP8	Partner of Y14 and mago	MEAAGSPAATETGK	N-Term(Acetyl); M1(Oxidation)	16.56	17.91	17.22	0.52	0.52	0.52	2.97	2.78	49.18	49.73	46.21	46.95	46.58	0.92	1.04	0.98
III	P23284	Peptidyl-prolyl cis-trans isomerase B	VLEGMEVVR	M5(Oxidation)	6.23	2.35	3.83	1.08	0.95	1.01	-1.47	3.56	-9.16	8.38	-7.69	4.82	-1.43	0.80	0.98	0.88
III	O60664	Perilipin-3	SVVTGGVQSVMGSR	M11(Oxidation)	3.80	4.17	3.98	0.85	0.93	0.89	5.20	2.27	19.76	9.47	14.56	7.20	10.88	1.07	0.94	1.01
III	Q00325	Phosphate carrier protein, mitochondrial	GVAPLWMR	M7(Oxidation)	4.56	2.71	3.51	0.79	0.83	0.81	5.57	9.10	25.40	24.62	19.83	15.52	17.68	0.68	0.76	0.72
III	P13796	Plastin-2	EGESLEDLMK	M9(Oxidation)	6.56	5.56	6.04	0.73	0.81	0.77	4.59	4.00	30.08	22.24	25.49	18.24	21.87	0.73	0.83	0.78
III	P11940	Polyadenylate-binding protein 1	QAHLTNQYMQR	N-Term(Gln->pyro-Glu); M9(Oxidation)	3.12	4.23	3.63	0.88	1.07	0.97	5.42	-2.36	16.93	-9.97	11.51	-7.61	1.95	0.82	1.06	0.93
III	Q6NZI2	Polymerase I and transcript release factor	ATEMVEVGADDDEGGAER	M4(Oxidation)	8.11	4.67	6.16	0.69	0.59	0.64	4.22	9.93	34.19	46.43	29.98	36.50	33.24	1.64	0.85	1.18
III	P07602	Proactivator polypeptide	LGPGMADICK	M5(Oxidation); C9(Carbamidomethyl)	3.24	4.01	3.60	0.88	0.92	0.90	4.99	2.44	16.15	9.77	11.16	7.33	9.25	1.23	0.76	0.97
III	P12004	Proliferating cell nuclear antigen	LMDLDVEQLGIPEQEYSCVVK	M2(Oxidation); C18(Carbamidomethyl)	3.46	3.04	3.24	0.91	0.95	0.93	3.67	2.23	12.70	6.77	9.03	4.54	6.79	1.00	1.21	1.10
III	O14744	Protein arginine N-methyltransferase 5	AAMAVGGAGGSR	N-Term(Acetyl); M3(Oxidation)	3.68	3.98	3.83	0.96	0.92	0.94	1.35	2.68	4.97	10.66	3.62	7.98	5.80	0.86	1.01	0.93
III	Q9UKY7	Protein CDV3 homolog	VQAMQISSEKEEDDNEK	M4(Oxidation)	26.61	20.08	23.12	1.06	0.41	0.66	-0.22	2.98	-5.97	59.76	-5.74	56.79	25.52	0.84	0.76	0.80

III	Q96A26	Protein FAM162A	EDEIPETVSLEMLDAAK	M12(Oxidation)	18.22	14.72	16.37	0.63	0.49	0.56	2.12	3.56	38.66	52.38	36.54	48.82	42.68	2.68	0.25	0.81
III	Q15437	Protein transport protein Sec23B	MVVPLACLLTPLK	M1(Oxidation); C7(Carbamidomethyl)	4.52	6.21	5.30	0.93	0.98	0.95	2.00	0.35	9.03	2.18	7.03	1.83	4.43	0.99	0.98	0.98
III	P50395	Rab GDP dissociation inhibitor beta	IPGSPPESMGR	M9(Oxidation)	2.81	2.64	2.72	0.92	0.83	0.87	4.20	9.54	11.82	25.17	7.62	15.63	11.62	1.00	1.16	1.08
III	Q15286	Ras-related protein Rab-35	FAGQMGIQLFETSAK	M5(Oxidation)	2.82	3.32	3.06	0.74	0.79	0.77	12.39	8.22	34.95	27.31	22.56	19.10	20.83	0.98	0.99	0.99
III	P52566	Rho GDP-dissociation inhibitor 2	ELQEMDKDDESLIK	M5(Oxidation)	3.63	4.38	3.98	0.76	0.69	0.72	8.29	8.45	30.07	36.99	21.78	28.54	25.16	1.43	1.08	1.24
III	P62877	RING-box protein 1	AAAMDVDTPSGTNSGAGK	N-Term(Acetyl); M4(Oxidation)	2.23	2.76	2.48	1.04	1.03	1.03	-3.05	-1.95	-6.79	-5.37	-3.75	-3.42	-3.58	1.10	1.19	1.15
III	Q9Y265	RuvB-like 1	EVYEGEVTELTPCETENPMGGYGK	C13(Carbamidomethyl); M19(Oxidation)	3.28	3.44	3.36	0.95	0.95	0.95	2.36	1.97	7.72	6.80	5.37	4.82	5.09	0.90	1.12	1.01
III	P30153	Serine/threonine-protein phosphatase 2A 65 kDa regulatory subunit A alpha isoform	VLAMSGDPNYLHR	M4(Oxidation)	2.53	2.40	2.47	1.03	0.90	0.96	-1.70	6.40	-4.30	15.39	-2.60	8.99	3.20	1.03	0.94	0.98
III	P05026	Sodium/potassium-transporting ATPase subunit beta-1	DDMIFEDCGDVPSEPK	M3(Oxidation); C8(Carbamidomethyl)	2.53	2.77	2.65	0.92	0.89	0.91	5.03	5.79	12.69	16.05	7.66	10.27	8.96	1.58	1.33	1.45
III	Q01082	Spectrin beta chain, brain 1	TQTAIASEDMPNTLTEAEK	M10(Oxidation)	12.28	3.69	6.73	0.70	0.66	0.68	2.61	11.31	32.06	41.73	29.45	30.43	29.94	1.25	0.64	0.90
III	Q14247	Src substrate cortactin	GPVSGTEPEPVYSMEAADYR	M14(Oxidation)	4.33	4.44	4.39	0.91	0.86	0.89	2.59	3.78	11.24	16.78	8.64	13.00	10.82	0.87	0.90	0.88
III	P38646	Stress-70 protein, mitochondrial	EQQIVIQSSGGLSKDDIENMVK	M20(Oxidation)	3.52	3.41	3.46	1.16	1.00	1.08	-6.56	-0.17	-23.08	-0.59	-16.52	-0.42	-8.47	0.78	0.98	0.88
III	P38646	Stress-70 protein, mitochondrial	ETGVDLTKDNMALQR	M11(Oxidation)	10.82	20.93	15.05	0.64	0.58	0.61	3.55	2.05	38.40	42.88	34.85	40.83	37.84	0.78	0.98	0.88
III	P38646	Stress-70 protein, mitochondrial	VEAVNMAEGIIHDTETK	M6(Oxidation)	3.72	3.70	3.71	0.77	0.97	0.87	7.69	1.16	28.56	4.28	20.87	3.12	12.00	0.78	0.98	0.88
III	O15260	Surfeit locus protein 4	MWFQWSEQR	M1(Oxidation)	3.31	3.38	3.34	0.88	1.01	0.94	5.07	-0.26	16.78	-0.87	11.70	-0.61	5.55	0.86	0.93	0.90
III	P50990	T-complex protein 1 subunit theta	APGFAQMLK	M7(Oxidation)	4.32	3.96	4.14	0.98	0.74	0.85	0.55	8.18	2.37	32.42	1.82	24.23	13.03	1.12	1.37	1.24
III	P37802	Transgelin-2	NVIGLQMGTNR	M7(Oxidation)	2.12	2.36	2.24	0.96	0.92	0.94	3.08	5.74	6.54	13.58	3.46	7.83	5.64	1.39	1.19	1.29
III	P37802	Transgelin-2	QMEQISQFLQAAER	M2(Oxidation)	14.39	7.18	10.17	0.72	0.43	0.56	2.06	8.40	29.71	60.35	27.64	51.95	39.80	1.39	1.19	1.29
III	P55072	Transitional endoplasmic reticulum ATPase	KYEMFAQTLQQSR	M4(Oxidation)	12.30	9.04	10.55	0.81	1.06	0.93	1.64	-0.77	20.19	-7.00	18.55	-6.23	6.16	0.92	1.00	0.96
III	P13693	Translationally-controlled tumor protein	VKPFMTGAAEQIK	M5(Oxidation)	11.23	4.56	7.16	0.65	0.63	0.64	3.34	9.39	37.47	42.81	34.14	33.43	33.78	0.83	0.93	0.88
III	Q9BVK6	Transmembrane emp24 domain-containing protein 9	FSLFAGGMLR	M8(Oxidation)	8.87	4.85	6.56	0.69	0.52	0.60	3.84	11.08	34.03	53.73	30.19	42.65	36.42	0.93	0.93	0.93
III	P40939	Trifunctional enzyme subunit alpha, mitochondrial	SAVLISSKPGCFIAGADINMLAACK	C11(Carbamidomethyl); M20(Oxidation); C24(Carbamidomethyl)	2.86	2.90	2.88	0.86	0.86	0.86	7.07	6.70	20.27	19.40	13.19	12.70	12.95	1.05	1.11	1.08
III	Q9BQE3	Tubulin alpha-1C chain	AVCMLSNTTAVAEAWAR	C3(Carbamidomethyl); M4(Oxidation)	6.83	3.66	5.00	0.76	0.83	0.79	3.89	6.10	26.57	22.35	22.69	16.25	19.47	0.90	1.08	0.99
III	P07437	Tubulin beta chain	MAVTFIGNSTAIQELFK	M1(Oxidation)	25.73	10.92	16.76	0.46	0.41	0.44	2.14	5.58	55.15	60.86	53.00	55.29	54.15	1.06	1.06	1.06
III	P09758	Tumor-associated calcium signal transducer 2	MTVCSPDGPGGR	M1(Oxidation); C4(Carbamidomethyl)	7.13	2.10	3.87	0.63	0.73	0.68	5.73	19.83	40.81	41.58	35.08	21.75	28.42	5.96	0.13	0.88
III	Q9BV40	Vesicle-associated membrane protein 8	MEEASEGGGNDR	N-Term(Acetyl); M1(Oxidation)	8.41	8.92	8.66	0.45	0.63	0.53	6.94	4.42	58.41	39.41	51.47	34.99	43.23	2.44	0.43	1.02

Table S6. List of potential Photofrin-binding proteins identified by affinity purification coupled with quantitative proteomics approach.
A total of 803 proteins were identified as potential Photofrin-interacting proteins (mean P/C Log2 ratio >1.1 in swapping experiments; Exp.1, Photofrin/Ctrl = Light/Heavy; Exp. 2, Photofrin/Ctrl = Heavy/Light). Among them, 94 proteins were identified as being severely oxidized under Photofrin-PDT (Table S2) and are indicated in this table with "a".

Protein accession No.	Description	P/C=L/H (Log2 ratio)	P/C=H/L (Log2 ratio)	mean	Severely oxidized[a]
Q9BUP3	Oxidoreductase HTATIP2	13.98	12.07	13.03	
Q9Y2R0	Cytochrome c oxidase assembly factor 3 homolog	14.56	11.40	12.98	
Q9HBL7	Plasminogen receptor (KT)	11.10	14.67	12.88	
Q6ZXV5	Transmembrane and TPR repeat-containing protein 3	12.65	12.72	12.68	
A6NHL2	Tubulin alpha chain-like 3	14.80	10.36	12.58	
Q58FF6	Putative heat shock protein HSP 90-beta 4	13.26	11.47	12.37	
Q9Y277	Voltage-dependent anion-selective channel protein 3	12.52	12.18	12.35	
O75964	ATP synthase subunit	11.80	12.83	12.32	
Q53GQ0	Very-long-chain 3-oxoacyl-CoA reductase	12.71	11.92	12.31	
Q9UM00	Transmembrane and coiled-coil domain-containing protein 1	14.52	9.97	12.24	
Q9P032	NADH dehydrogenase [ubiquinone] 1 alpha subcomplex assembly factor 4	12.53	11.78	12.16	
P60059	Protein transport protein Sec61 subunit gamma	12.04	12.15	12.10	
P29034	Protein S100-A2	12.36	11.69	12.03	
Q9NRV9	Heme-binding protein 1	12.51	11.32	11.92	
Q69YL0	Uncharacterized protein NCBP2-AS2	12.54	10.81	11.68	
Q8N5K1	CDGSH iron-sulfur domain-containing protein 2	12.17	11.09	11.63	
P50281	Matrix metalloproteinase-14	11.16	11.80	11.48	
Q96IX5	Up-regulated during skeletal muscle growth protein 5	11.86	10.96	11.41	
P12235	ADP/ATP translocase 1	10.82	11.97	11.40	
Q5JTJ3	Cytochrome c oxidase assembly factor 6 homolog	12.09	10.57	11.33	
Q6ZS86	Putative glycerol kinase 5	11.59	11.02	11.30	
Q14435	Polypeptide N-acetylgalactosaminyltransferase 3	11.94	10.65	11.29	a
Q9BRQ8	Apoptosis-inducing factor 2	12.04	10.49	11.27	
Q96A26	Protein FAM162A	12.72	9.75	11.23	a
Q8N1F7	Nuclear pore complex protein Nup93	11.84	10.50	11.17	
Q9Y3B3	Transmembrane emp24 domain-containing protein 7	11.30	11.02	11.16	
Q9Y241	HIG1 domain family member 1A	11.72	10.52	11.12	
Q6NUK1	Calcium-binding mitochondrial carrier protein SCaMC-1	11.72	10.40	11.06	a
O15173	Membrane-associated progesterone receptor component 2	11.78	10.29	11.04	a
P43304	Glycerol-3-phosphate dehydrogenase	11.19	10.86	11.03	a
Q15363	Transmembrane emp24 domain-containing protein 2	11.75	10.19	10.97	
P06703	Protein S100-A6	10.79	11.13	10.96	
Q9Y320	Thioredoxin-related transmembrane protein 2	11.41	10.50	10.95	
Q92797	Symplekin	11.34	10.44	10.89	
P20340	Ras-related protein Rab-6A	12.08	9.57	10.83	
P21796	Voltage-dependent anion-selective channel protein 1	11.42	10.21	10.82	a
Q96DA6	Mitochondrial import inner membrane translocase subunit TIM14	11.62	9.99	10.81	
Q03518	Antigen peptide transporter 1	10.41	11.03	10.72	
O60704	Protein-tyrosine sulfotransferase 2	11.86	9.52	10.69	
Q8NBX0	Saccharopine dehydrogenase-like oxidoreductase	10.59	10.74	10.67	
Q9Y512	Sorting and assembly machinery component 50 homolog	12.29	9.03	10.66	
Q6P1Q0	LETM1 domain-containing protein 1	11.14	10.14	10.64	
Q9H9B4	Sideroflexin-1	11.49	9.76	10.62	
Q9UBE0	SUMO-activating enzyme subunit 1	9.87	11.31	10.59	
Q6UXV4	MIC complex subunit MIC27	11.22	9.89	10.55	
Q86SF2	N-acetylgalactosaminyltransferase 7	10.46	10.60	10.53	
Q969V3	Nicalin	11.16	9.87	10.52	
O14981	TATA-binding protein-associated factor 172	13.75	7.24	10.50	
Q8TC12	Retinol dehydrogenase 11	11.09	9.89	10.49	
Q969Z3	Mitochondrial amidoxime reducing component 2	11.93	9.03	10.48	
Q9C0E8	Protein lunapark	9.51	11.41	10.46	
Q9Y2Q3	Glutathione S-transferase kappa 1	11.52	9.37	10.44	a
P49755	Transmembrane emp24 domain-containing protein 10	11.43	9.40	10.41	a
O00116	Alkyldihydroxyacetonephosphate synthase	11.20	9.57	10.39	
Q96GE9	Transmembrane protein 261	11.73	9.02	10.38	
Q9BZG1	Ras-related protein Rab-34	11.07	9.68	10.37	
O76095	Protein JTB	13.15	7.58	10.37	
P48960	CD97 antigen	10.99	9.73	10.36	
Q02127	Dihydroorotate dehydrogenase (quinone)	9.95	10.76	10.35	
Q7LGA3	Heparan sulfate 2-O-sulfotransferase 1	11.26	9.41	10.34	
P62072	Mitochondrial import inner membrane translocase subunit Tim10	11.09	9.58	10.33	
Q7L5L3	Glycerophosphodiester phosphodiesterase domain-containing protein 3	10.67	9.98	10.32	

Q5VYK3	Proteasome-associated protein ECM29 homolog	10.92	9.71	10.32	a
O43837	Isocitrate dehydrogenase [NAD] subunit beta	11.43	9.17	10.30	
Q99623	Prohibitin-2	10.37	10.22	10.30	
Q969N2	GPI transamidase component PIG-T	10.49	10.09	10.29	
P51153	Ras-related protein Rab-13	11.80	8.73	10.27	
Q96EE4	Coiled-coil domain-containing protein 126	11.61	8.91	10.26	
Q8N2G8	GH3 domain-containing protein	10.53	9.86	10.19	
O95202	LETM1 and EF-hand domain-containing protein 1	10.75	9.63	10.19	
O75477	Erlin-1	10.81	9.57	10.19	
P60953	Cell division control protein 42 homolog	10.88	9.49	10.19	
Q9Y6C9	Mitochondrial carrier homolog 2	11.29	9.06	10.17	
Q10471	Polypeptide N-acetylgalactosaminyltransferase 2	10.55	9.79	10.17	
Q9Y5J6	Mitochondrial import inner membrane translocase subunit Tim10 B	11.93	8.39	10.16	
P49748	Very long-chain specific acyl-CoA dehydrogenase	10.87	9.44	10.15	
Q9BVC4	Target of rapamycin complex subunit LST8	10.93	9.37	10.15	
Q96TA2	ATP-dependent zinc metalloprotease YME1L1	10.43	9.85	10.14	
Q9UH99	SUN domain-containing protein 2	10.89	9.37	10.13	
Q9P0U1	Mitochondrial import receptor subunit TOM7 homolog	12.32	7.93	10.12	
Q9H845	Acyl-CoA dehydrogenase family member 9	10.05	10.19	10.12	
Q53GS7	Nucleoporin GLE1	10.92	9.30	10.11	
Q68D91	Metallo-beta-lactamase domain-containing protein 2	11.13	9.09	10.11	
Q5JPH6	Probable glutamate--tRNA ligase	11.16	9.04	10.10	
O75431	Metaxin-2	10.27	9.87	10.07	
A1L0T0	Acetolactate synthase-like protein	12.03	8.10	10.07	
P38606	V-type proton ATPase catalytic subunit A	10.38	9.75	10.06	
Q8NBT2	Kinetochore protein Spc24	10.50	9.58	10.04	
O43615	Mitochondrial import inner membrane translocase subunit TIM44	10.92	9.14	10.03	
O94874	E3 UFM1-protein ligase 1	10.54	9.51	10.03	
Q70UQ0	Inhibitor of nuclear factor kappa-B kinase-interacting protein	9.96	10.07	10.02	
Q92544	Transmembrane 9 superfamily member 4	10.57	9.44	10.01	a
P60602	Reactive oxygen species modulator 1	10.25	9.73	9.99	
O75911	Short-chain dehydrogenase/reductase 3	9.94	10.03	9.99	
P06702	Protein S100-A9	10.54	9.37	9.95	
P61160	Actin-related protein 2	10.41	9.48	9.95	
Q02978	Mitochondrial 2-oxoglutarate/malate carrier protein	10.59	9.27	9.93	
Q8NCL4	Polypeptide N-acetylgalactosaminyltransferase 6	10.65	9.20	9.93	
P56378	6.8 kDa mitochondrial proteolipid	9.81	10.03	9.92	
O60488	Long-chain-fatty-acid--CoA ligase 4	9.32	10.51	9.91	
O15270	Serine palmitoyltransferase 2	10.28	9.52	9.90	a
Q8N4V1	Membrane magnesium transporter 1	9.98	9.79	9.89	
Q9Y6E2	Basic leucine zipper and W2 domain-containing protein 2	10.12	9.64	9.88	
Q9UBF2	Coatomer subunit gamma-2	10.79	8.94	9.87	
Q9UP83	Conserved oligomeric Golgi complex subunit 5	10.30	9.43	9.86	
Q9Y3D7	Mitochondrial import inner membrane translocase subunit TIM16	10.78	8.94	9.86	
Q86VP6	Cullin-associated NEDD8-dissociated protein 1	9.75	9.96	9.85	
Q9HBH5	Retinol dehydrogenase 14	9.77	9.91	9.84	
Q03519	Antigen peptide transporter 2	9.53	10.14	9.83	
P33240	Cleavage stimulation factor subunit 2	10.82	8.83	9.83	
Q92520	Protein FAM3C	10.31	9.31	9.81	
P42704	Leucine-rich PPR motif-containing protein	11.17	8.45	9.81	a
Q9GZY8	Mitochondrial fission factor	10.12	9.49	9.81	
Q9NX40	OCIA domain-containing protein 1	11.06	8.53	9.80	
P33121	Long-chain-fatty-acid--CoA ligase 1	10.58	8.99	9.79	a
P43353	Aldehyde dehydrogenase family 3 member B1	10.90	8.63	9.77	
Q9H4I3	TraB domain-containing protein	10.42	9.12	9.77	
Q9Y5J7	Mitochondrial import inner membrane translocase subunit Tim9	10.14	9.29	9.71	
Q96ER9	Coiled-coil domain-containing protein 51	9.51	9.92	9.71	
Q9H7Z7	Prostaglandin E synthase 2	10.85	8.57	9.71	
P54920	Alpha-soluble NSF attachment protein	10.20	9.15	9.67	
Q96HY6	DDRGK domain-containing protein 1	10.03	9.31	9.67	
Q96I36	Cytochrome c oxidase assembly protein COX14	9.88	9.42	9.65	
Q9NP72	Ras-related protein Rab-18	10.21	9.08	9.64	
Q8WTV0	Scavenger receptor class B member 1	12.42	6.85	9.63	
Q9BTX1	Nucleoporin NDC1	12.33	6.93	9.63	
Q5UCC4	ER membrane protein complex subunit 10	10.87	8.36	9.62	
Q9NR77	Peroxisomal membrane protein 2	10.40	8.82	9.61	
Q99595	Mitochondrial import inner membrane translocase subunit Tim17-A	11.19	8.03	9.61	
Q9NX14	NADH dehydrogenase [ubiquinone] 1 beta subcomplex subunit 11	10.18	9.02	9.60	
Q29RF7	Sister chromatid cohesion protein PDS5 homolog A	10.00	9.17	9.59	

O43264	Centromere/kinetochore protein zw10 homolog	10.13	8.94	9.54	
O96008	Mitochondrial import receptor subunit TOM40 homolog	10.06	9.01	9.53	a
Q16864	V-type proton ATPase subunit F	10.37	8.66	9.52	
O75027	ATP-binding cassette sub-family B member 7	10.16	8.86	9.51	
Q8TB36	Ganglioside-induced differentiation-associated protein 1	10.06	8.96	9.51	
Q9UFG5	UPF0449 protein C19orf25	10.49	8.52	9.51	
Q9NP84	Tumor necrosis factor receptor superfamily member 12A	11.33	7.69	9.51	
Q8N4H5	Mitochondrial import receptor subunit TOM5 homolog	8.01	10.99	9.50	
Q9BQE5	Apolipoprotein L2	10.05	8.89	9.47	
O60830	Mitochondrial import inner membrane translocase subunit Tim17-B	10.02	8.90	9.46	
P98172	Ephrin-B1	9.66	9.24	9.45	
Q9H0P0	Cytosolic 5'-nucleotidase 3A	9.73	9.16	9.44	
Q9HB09	Bcl-2-like protein 12	9.41	9.44	9.42	
Q5TGZ0	MIC complex subunit MIC10	9.59	9.25	9.42	
Q8NBJ4	Golgi membrane protein 1	11.88	6.92	9.40	
O75146	Huntingtin-interacting protein 1-related protein	9.94	8.85	9.40	
Q8NF37	Lysophosphatidylcholine acyltransferase 1	8.84	9.90	9.37	
Q8N1B4	Vacuolar protein sorting-associated protein 52 homolog	8.76	9.96	9.36	a
O75427	Leucine-rich repeat and calponin homology domain-containing protein 4	10.22	8.44	9.33	
Q9HDC9	Adipocyte plasma membrane-associated protein	9.27	9.34	9.30	
Q7Z434	Mitochondrial antiviral-signaling protein	10.11	8.48	9.30	
Q9UL46	Proteasome activator complex subunit 2	11.21	7.35	9.28	
P30043	Flavin reductase (NADPH)	10.43	8.13	9.28	
P51648	Fatty aldehyde dehydrogenase	10.37	8.16	9.27	
Q9UHR4	Brain-specific angiogenesis inhibitor 1-associated protein 2-like protein 1	9.75	8.78	9.26	
Q92616	Translational activator GCN1	10.25	8.26	9.25	
Q8WVM8	Sec1 family domain-containing protein 1	10.97	7.45	9.21	
O94826	Mitochondrial import receptor subunit TOM70	9.97	8.44	9.20	a
O95249	Golgi SNAP receptor complex member 1	9.73	8.65	9.19	
Q96FV9	THO complex subunit 1	10.14	8.23	9.18	
Q9NRR3	CDC42 small effector protein 2	9.48	8.86	9.17	
P57105	Synaptojanin-2-binding protein	9.98	8.35	9.16	
Q15628	Tumor necrosis factor receptor type 1-associated DEATH domain protein	9.95	8.37	9.16	
P50151	Guanine nucleotide-binding protein G(I)/G(S)/G(O) subunit gamma-10	9.10	9.21	9.16	
Q9UH62	Armadillo repeat-containing X-linked protein 3	8.79	9.51	9.15	
Q96D53	AarF domain-containing protein kinase 4	8.47	9.83	9.15	
O60313	Dynamin-like 120 kDa protein	9.37	8.91	9.14	
Q9H3K2	Growth hormone-inducible transmembrane protein	10.54	7.65	9.09	
Q8NBN7	Retinol dehydrogenase 13	9.68	8.48	9.08	
Q9NRK6	ATP-binding cassette sub-family B member 10	8.85	9.28	9.07	
Q8WU76	Sec1 family domain-containing protein 2	9.73	8.36	9.04	
Q2TAA5	GDP-Man:Man(3)GlcNAc(2)-PP-Dol alpha-1,2-mannosyltransferase	9.38	8.69	9.03	
Q9BUR5	MIC complex subunit MIC26	10.20	7.81	9.00	
Q7L1Q6	Basic leucine zipper and W2 domain-containing protein 1	9.64	8.33	8.99	
P37268	Squalene synthase	9.54	8.40	8.97	
Q14739	Lamin-B receptor	8.38	9.39	8.89	
Q9UBV8	Peflin	11.93	5.84	8.89	
Q14746	Conserved oligomeric Golgi complex subunit 2	10.31	7.43	8.87	
Q9Y276	Mitochondrial chaperone BCS1	9.19	8.48	8.83	
P62256	Ubiquitin-conjugating enzyme E2 H	8.56	9.08	8.82	
Q9BRT2	Ubiquinol-cytochrome-c reductase complex assembly factor 2	9.13	8.50	8.82	
Q9BVK6	Transmembrane emp24 domain-containing protein 9	11.51	6.12	8.81	a
Q9NWT8	Aurora kinase A-interacting protein	9.23	8.28	8.76	
O95219	Sorting nexin-4	9.03	8.49	8.76	
Q969P0	Immunoglobulin superfamily member 8	9.38	8.10	8.74	
O75396	Vesicle-trafficking protein SEC22b	9.19	8.26	8.73	a
Q9UIA9	Exportin-7	8.75	8.66	8.71	
P22830	Ferrochelatase	10.67	6.71	8.69	
P41440	Folate transporter 1	8.71	8.60	8.65	
Q9C002	Normal mucosa of esophagus-specific gene 1 protein	11.45	5.84	8.64	
Q5HYI8	Rab-like protein 3	9.35	7.93	8.64	
Q6IAN0	Dehydrogenase/reductase SDR family member 7B	10.58	6.60	8.59	
P31949	Protein S100-A11	8.02	9.13	8.57	
Q32NC0	UPF0711 protein C18orf21	6.78	10.33	8.56	
P05556	Integrin beta-1	9.98	7.12	8.55	a
Q92947	Glutaryl-CoA dehydrogenase	9.12	7.98	8.55	
Q9NX63	MIC complex subunit MIC19	9.57	7.51	8.54	a
Q9HBM1	Kinetochore protein Spc25	10.84	6.18	8.51	
O75251	NADH dehydrogenase [ubiquinone] iron-sulfur protein 7	12.51	4.51	8.51	

Q8N2F6	Armadillo repeat-containing protein 10	8.55	8.46	8.50	
P51148	Ras-related protein Rab-5C	10.29	6.65	8.47	a
O95801	Tetratricopeptide repeat protein 4	8.65	8.28	8.46	
P0DJI8	Serum amyloid A-1 protein	9.37	7.55	8.46	
P18859	ATP synthase-coupling factor 6	10.27	6.59	8.43	
Q9UI12	V-type proton ATPase subunit H	9.06	7.74	8.40	
Q92621	Nuclear pore complex protein Nup205	9.15	7.65	8.40	
Q56VL3	OCIA domain-containing protein 2	10.12	6.60	8.36	
Q05209	Tyrosine-protein phosphatase non-receptor type 12	9.25	7.44	8.35	
P60604	Ubiquitin-conjugating enzyme E2 G2	7.00	9.67	8.33	
P20674	Cytochrome c oxidase subunit 5A	10.46	6.16	8.31	
O00410	Importin-5	9.14	7.47	8.30	a
P02686	Myelin basic protein	8.13	8.27	8.20	
Q9BWM7	Sideroflexin-3	10.01	6.25	8.13	
P50416	Carnitine O-palmitoyltransferase 1, liver isoform	8.48	7.76	8.12	a
Q5RI15	Cytochrome c oxidase protein 20 homolog	9.20	7.01	8.11	
Q16890	Tumor protein D53	5.07	11.09	8.08	
P61966	AP-1 complex subunit sigma-1A	8.70	7.44	8.07	
O95573	Long-chain-fatty-acid--CoA ligase 3	7.16	8.97	8.06	
P56589	Peroxisomal biogenesis factor 3	6.56	9.54	8.05	
Q8IWL3	Iron-sulfur cluster co-chaperone protein HscB	6.65	9.42	8.03	
Q9BU23	Lipase maturation factor 2	8.78	7.28	8.03	
Q86WV6	Stimulator of interferon genes protein	10.38	5.61	7.99	
P49585	Choline-phosphate cytidylyltransferase A	9.58	6.40	7.99	
P35232	Prohibitin	9.69	6.29	7.99	
O94919	Endonuclease domain-containing 1 protein	9.23	6.72	7.98	
P56556	NADH dehydrogenase [ubiquinone] 1 alpha subcomplex subunit 6	4.98	10.93	7.96	
Q9UHQ9	NADH-cytochrome b5 reductase 1	8.41	7.51	7.96	
Q9NVH1	DnaJ homolog subfamily C member 11	9.21	6.64	7.93	
O15260	Surfeit locus protein 4	8.98	6.82	7.90	a
Q9NTI5	Sister chromatid cohesion protein PDS5 homolog B	8.12	7.68	7.90	
Q9UKV5	E3 ubiquitin-protein ligase AMFR	8.36	7.39	7.87	
P63000	Ras-related C3 botulinum toxin substrate 1	11.21	4.50	7.85	
Q9ULC5	Long-chain-fatty-acid--CoA ligase 5	7.39	8.30	7.85	
Q96NB2	Sideroflexin-2	10.33	5.34	7.84	
Q8NBU5	ATPase family AAA domain-containing protein 1	4.41	11.26	7.83	
P56134	ATP synthase subunit f	4.45	11.21	7.83	
Q8N183	Mimitin	9.48	6.15	7.81	
Q00059	Transcription factor A	2.84	12.78	7.81	
Q9Y6K5	2'-5'-oligoadenylate synthase 3	10.67	4.86	7.77	
Q9H0U4	Ras-related protein Rab-1B	4.23	11.28	7.76	a
P0CW22	40S ribosomal protein S17-like	8.74	6.75	7.74	
Q86UD0	Suppressor APC domain-containing protein 2	9.26	6.22	7.74	
Q9NZV5	Selenoprotein N	8.49	6.97	7.73	
P15153	Ras-related C3 botulinum toxin substrate 2	7.98	7.29	7.64	
P42766	60S ribosomal protein L35	10.46	4.79	7.63	
Q6P4A7	Sideroflexin-4	5.30	9.93	7.62	
P21912	Succinate dehydrogenase [ubiquinone] iron-sulfur subunit	7.44	7.75	7.60	
Q0VGL1	Ragulator complex protein LAMTOR4	3.42	11.72	7.57	
Q9BU61	NADH dehydrogenase [ubiquinone] 1 alpha subcomplex assembly factor 3	9.82	5.32	7.57	
Q9NZ43	Vesicle transport protein USE1	8.27	6.80	7.54	
P02649	Apolipoprotein E	3.76	11.30	7.53	
Q9NVI7	ATPase family AAA domain-containing protein 3A	10.70	4.34	7.52	
Q00325	Phosphate carrier protein	11.48	3.54	7.51	a
Q15392	Delta(24)-sterol reductase	11.17	3.85	7.51	a
P56385	ATP synthase subunit e	8.93	6.05	7.49	
Q15006	ER membrane protein complex subunit 2	9.36	5.58	7.47	
Q9BQD3	KxDL motif-containing protein 1	5.15	9.78	7.47	
P20591	Interferon-induced GTP-binding protein Mx1	5.60	9.33	7.46	
Q9BRQ6	MIC complex subunit MIC25	9.02	5.89	7.46	
Q9UNL2	Translocon-associated protein subunit gamma	1.57	13.31	7.44	
O00124	UBX domain-containing protein 8	7.90	6.88	7.39	
P29966	Myristoylated alanine-rich C-kinase substrate	8.31	6.46	7.38	
P16422	Epithelial cell adhesion molecule	11.79	2.94	7.36	a
P45880	Voltage-dependent anion-selective channel protein 2	9.12	5.57	7.35	
P61225	Ras-related protein Rap-2b	4.20	10.42	7.31	
Q6IN85	Serine/threonine-protein phosphatase 4 regulatory subunit 3A	10.62	3.97	7.30	
P21583	Kit ligand	8.04	6.51	7.28	
P30049	ATP synthase subunit delta	9.79	4.76	7.28	

Q6YN16	Hydroxysteroid dehydrogenase-like protein 2	11.20	3.35	7.28	
Q96S66	Chloride channel CLIC-like protein 1	8.84	5.68	7.26	a
P36542	ATP synthase subunit gamma	10.55	3.91	7.23	
P33981	Dual specificity protein kinase TTK	10.73	3.73	7.23	
Q9BPX5	Actin-related protein 2/3 complex subunit 5-like protein	11.89	2.56	7.23	
Q8NCE2	Myotubularin-related protein 14	11.00	3.38	7.19	
P84095	Rho-related GTP-binding protein RhoG	10.47	3.90	7.18	
P61020	Ras-related protein Rab-5B	10.11	4.22	7.17	
P24539	ATP synthase F(0) complex subunit B1	3.62	10.52	7.07	
Q96B49	Mitochondrial import receptor subunit TOM6 homolog	4.55	9.56	7.05	
O75306	NADH dehydrogenase [ubiquinone] iron-sulfur protein 2	10.34	3.75	7.04	
Q6PIJ6	F-box only protein 38	7.99	6.09	7.04	
Q6P3X3	Tetratricopeptide repeat protein 27	5.25	8.68	6.97	
Q6P1M0	Long-chain fatty acid transport protein 4	8.52	5.28	6.90	
P09669	Cytochrome c oxidase subunit 6C	8.32	5.46	6.89	
Q96NT0	Coiled-coil domain-containing protein 115	10.31	3.39	6.85	
Q5XKP0	MIC complex subunit MIC13	10.03	3.63	6.83	
P31040	Succinate dehydrogenase [ubiquinone] flavoprotein subunit	7.14	6.51	6.82	
Q92973	Transportin-1	9.89	3.69	6.79	
Q6NZ67	Mitotic-spindle organizing protein 2B	7.67	5.88	6.78	
Q9NP73	Putative bifunctional UDP-N-acetylglucosamine transferase and deubiquitinase ALG13	11.07	2.42	6.74	
E0CX11	Uncharacterized protein C7orf73	4.61	8.86	6.73	
O76071	Probable cytosolic iron-sulfur protein assembly protein CIAO1	7.65	5.78	6.72	
Q9UBI6	Guanine nucleotide-binding protein G(I)/G(S)/G(O) subunit gamma-12	8.94	4.47	6.71	a
Q9Y6M9	NADH dehydrogenase [ubiquinone] 1 beta subcomplex subunit 9	3.05	10.34	6.69	
Q7Z7L1	Schlafen family member 11	11.08	2.29	6.69	
O14925	Mitochondrial import inner membrane translocase subunit Tim23	6.97	6.38	6.68	
Q7Z2K6	Endoplasmic reticulum metallopeptidase 1	10.20	3.14	6.67	a
Q8IUH4	Palmitoyltransferase ZDHHC13	10.31	3.01	6.66	
P11233	Ras-related protein Ral-A	6.21	7.09	6.65	
Q96BP2	Coiled-coil-helix-coiled-coil-helix domain-containing protein 1	5.59	7.70	6.65	
Q96FZ7	Charged multivesicular body protein 6	7.22	6.06	6.64	
Q14558	Phosphoribosyl pyrophosphate synthase-associated protein 1	9.63	3.64	6.64	
Q9Y613	FH1/FH2 domain-containing protein 1	9.93	3.25	6.59	
O43402	ER membrane protein complex subunit 8	10.14	3.03	6.59	
Q9UPT5	Exocyst complex component 7	10.80	2.31	6.55	
O14929	Histone acetyltransferase type B catalytic subunit	8.81	4.29	6.55	
O94905	Erlin-2	10.03	3.05	6.54	
O43847	Nardilysin	7.29	5.73	6.51	
P63244	Guanine nucleotide-binding protein subunit beta-2-like 1	7.29	5.70	6.49	a
P57086	SCAN domain-containing protein 1	9.85	3.12	6.49	
Q16891	MIC complex subunit MIC60	7.47	5.45	6.46	a
Q96EI5	Transcription elongation factor A protein-like 4	7.92	4.97	6.45	
P55957	BH3-interacting domain death agonist	8.28	4.60	6.44	
P62158	Calmodulin	10.04	2.82	6.43	a
Q86VU5	Catechol O-methyltransferase domain-containing protein 1	2.97	9.86	6.42	
P56381	ATP synthase subunit epsilon	8.25	4.56	6.41	
Q712K3	Ubiquitin-conjugating enzyme E2 R2	9.21	3.59	6.40	
P30044	Peroxiredoxin-5	8.23	4.56	6.40	
P50895	Basal cell adhesion molecule	8.93	3.80	6.37	a
P30519	Heme oxygenase 2	8.59	4.09	6.34	
P42167	Lamina-associated polypeptide 2, isoforms beta/gamma	10.79	1.87	6.33	
Q86SX3	Uncharacterized protein C14orf80	9.98	2.66	6.32	
Q9UIV1	CCR4-NOT transcription complex subunit 7	3.49	9.15	6.32	
P07602	Prosaposin	10.12	2.51	6.31	a
P49591	Serine--tRNA ligase	11.62	1.01	6.31	a
Q13158	FAS-associated death domain protein	6.95	5.66	6.31	a
Q15388	Mitochondrial import receptor subunit TOM20 homolog	7.79	4.78	6.28	
P04083	Annexin A1	3.06	9.44	6.25	a
Q99615	DnaJ homolog subfamily C member 7	8.39	4.10	6.25	
P40938	Replication factor C subunit 3	8.35	4.08	6.21	
P11413	Glucose-6-phosphate 1-dehydrogenase	7.20	5.19	6.19	
Q9NS69	Mitochondrial import receptor subunit TOM22 homolog	7.18	5.21	6.19	a
Q9UII2	ATPase inhibitor	8.20	4.14	6.17	
P07942	Laminin subunit beta-1	1.92	10.41	6.16	
Q8NBI6	Xyloside xylosyltransferase 1	3.26	9.06	6.16	
Q99961	Endophilin-A2	10.78	1.53	6.16	
Q15021	Condensin complex subunit 1	9.61	2.69	6.15	

P51571	Translocon-associated protein subunit delta	9.84	2.44	6.14	
Q8NBM4	Ubiquitin-associated domain-containing protein 2	8.36	3.89	6.13	
P55327	Tumor protein D52	9.11	3.13	6.12	
Q9BX59	Tapasin-related protein	10.33	1.91	6.12	
P06576	ATP synthase subunit beta	6.36	5.76	6.06	a
Q06210	Glutamine--fructose-6-phosphate aminotransferase [isomerizing] 1	8.54	3.44	5.99	
Q8NBZ7	UDP-glucuronic acid decarboxylase 1	6.43	5.55	5.99	
P98179	RNA-binding protein 3	11.61	0.35	5.98	
Q92478	C-type lectin domain family 2 member B	9.04	2.90	5.97	
O15304	Apoptosis regulatory protein Siva	9.29	2.64	5.96	
O43824	Putative GTP-binding protein 6	2.10	9.82	5.96	
P49006	MARCKS-related protein	6.59	5.31	5.95	
Q99653	Calcineurin B homologous protein 1	7.27	4.50	5.88	
P17706	Tyrosine-protein phosphatase non-receptor type 2	8.74	3.01	5.87	
P62820	Ras-related protein Rab-1A	4.95	6.79	5.87	
Q9P0L0	Vesicle-associated membrane protein-associated protein A	11.49	0.23	5.86	a
Q12774	Rho guanine nucleotide exchange factor 5	9.31	2.41	5.86	
P01889	HLA class I histocompatibility antigen, B-7 alpha chain	10.39	1.31	5.85	
Q96M89	Coiled-coil domain-containing protein 138	8.84	2.84	5.84	
P06493	Cyclin-dependent kinase 1	9.38	2.30	5.84	
Q13015	Protein AF1q	10.10	1.57	5.83	
Q562E7	WD repeat-containing protein 81	11.38	0.28	5.83	
Q9NQR4	Omega-amidase NIT2	8.73	2.91	5.82	
Q9Y3E5	Peptidyl-tRNA hydrolase 2	8.77	2.85	5.81	
Q6IAA8	Ragulator complex protein LAMTOR1	3.82	7.78	5.80	
P50552	Vasodilator-stimulated phosphoprotein	6.30	5.29	5.79	
Q9Y312	Protein AAR2 homolog	9.72	1.83	5.78	
Q9C0B5	Palmitoyltransferase ZDHHC5	9.51	2.02	5.76	
Q9Y371	Endophilin-B1	8.80	2.69	5.75	a
P51617	Interleukin-1 receptor-associated kinase 1	9.49	1.96	5.72	
P62873	Guanine nucleotide-binding protein G(I)/G(S)/G(T) subunit beta-1	8.45	2.97	5.71	a
Q9P0J0	NADH dehydrogenase [ubiquinone] 1 alpha subcomplex subunit 13	7.31	4.07	5.69	
Q9H3H1	tRNA dimethylallyltransferase	10.97	0.41	5.69	
Q9HAV4	Exportin-5	7.32	4.04	5.68	
O43819	Protein SCO2 homolog	5.88	5.43	5.65	
O00232	26S proteasome non-ATPase regulatory subunit 12	11.01	0.29	5.65	
P11310	Medium-chain specific acyl-CoA dehydrogenase	9.07	2.21	5.64	
P11234	Ras-related protein Ral-B	8.62	2.62	5.62	
Q9UHR6	Zinc finger HIT domain-containing protein 2	1.93	9.26	5.60	
Q9UJU6	Drebrin-like protein	9.68	1.49	5.58	
O75340	Programmed cell death protein 6	6.84	4.28	5.56	
P08754	Guanine nucleotide-binding protein G(k) subunit alpha	7.75	3.35	5.55	
Q15024	Exosome complex component RRP42	7.72	3.36	5.54	
Q92896	Golgi apparatus protein 1	8.52	2.56	5.54	
P18669	Phosphoglycerate mutase 1	2.82	8.24	5.53	
O43169	Cytochrome b5 type B	9.26	1.79	5.53	
Q15910	Histone-lysine N-methyltransferase EZH2	10.07	0.97	5.52	
P30536	Translocator protein	5.25	5.75	5.50	
P12814	Alpha-actinin-1	9.06	1.91	5.48	a
P40937	Replication factor C subunit 5	10.58	0.39	5.48	
O60942	mRNA-capping enzyme	6.26	4.60	5.43	
Q9H4M9	EH domain-containing protein 1	9.32	1.52	5.42	a
P53007	Tricarboxylate transport protein	4.38	6.45	5.41	
Q8TDX7	Serine/threonine-protein kinase Nek7	2.01	8.76	5.39	
Q8WTT2	Nucleolar complex protein 3 homolog	2.21	8.56	5.38	
P04004	Vitronectin	1.39	9.37	5.38	
Q9NVU7	Protein SDA1 homolog	7.77	2.97	5.37	
Q53H12	Acylglycerol kinase	5.86	4.80	5.33	
O15392	Baculoviral IAP repeat-containing protein 5	1.14	9.48	5.31	
O43181	NADH dehydrogenase [ubiquinone] iron-sulfur protein 4	8.24	2.35	5.29	
P63172	Dynein light chain Tctex-type 1	9.63	0.94	5.29	
Q9NY27	Serine/threonine-protein phosphatase 4 regulatory subunit 2	8.70	1.86	5.28	
Q9NPD3	Exosome complex component RRP41	1.28	9.22	5.25	
Q9NRG9	Aladin	4.19	6.31	5.25	
P07204	Thrombomodulin	7.14	3.35	5.24	
Q9UEU0	Vesicle transport through interaction with t-SNAREs homolog 1B	7.30	3.17	5.23	
O00151	PDZ and LIM domain protein 1	9.42	1.00	5.21	
Q6UW78	Ubiquinol-cytochrome-c reductase complex assembly factor 3	1.79	8.62	5.20	
P47895	Aldehyde dehydrogenase family 1 member A3	6.78	3.62	5.20	

Q99417	C-Myc-binding protein	4.43	5.97	5.20	
P12236	ADP/ATP translocase 3	4.16	6.19	5.18	a
O15231	Zinc finger protein 185	9.29	1.04	5.16	
P14406	Cytochrome c oxidase subunit 7A2	5.46	4.86	5.16	
Q00587	Cdc42 effector protein 1	8.99	1.33	5.16	
Q8N766	ER membrane protein complex subunit 1	3.76	6.51	5.14	
Q6PIW4	Fidgetin-like protein 1	8.62	1.61	5.12	
Q9ULC4	Malignant T-cell-amplified sequence 1	1.38	8.83	5.10	
Q96EY5	Multivesicular body subunit 12A	10.09	0.09	5.09	
P53367	Arfaptin-1	3.53	6.61	5.07	
Q9HCY8	Protein S100-A14	5.39	4.73	5.06	a
Q9BW60	Elongation of very long chain fatty acids protein 1	5.36	4.69	5.03	a
P78346	Ribonuclease P protein subunit p30	9.28	0.74	5.01	
Q15185	Prostaglandin E synthase 3	7.05	2.97	5.01	
O75554	WW domain-binding protein 4	0.50	9.46	4.98	
Q96P11	Probable 28S rRNA (cytosine-C(5))-methyltransferase	1.42	8.52	4.97	
Q9UGV2	Protein NDRG3	0.72	9.15	4.93	
P50897	Palmitoyl-protein thioesterase 1	6.77	3.08	4.92	
Q15907	Ras-related protein Rab-11B	6.38	3.41	4.89	
P09629	Homeobox protein Hox-B7	8.18	1.57	4.88	
P61106	Ras-related protein Rab-14	6.73	3.01	4.87	
P60468	Protein transport protein Sec61 subunit beta	5.50	4.22	4.86	a
P62312	U6 snRNA-associated Sm-like protein LSm6	7.09	2.56	4.82	
Q9NR33	DNA polymerase epsilon subunit 4	3.48	6.17	4.82	
P10606	Cytochrome c oxidase subunit 5B	4.89	4.73	4.81	
P67812	Signal peptidase complex catalytic subunit SEC11A	7.69	1.90	4.80	
O75947	ATP synthase subunit d	5.33	4.16	4.74	
P05362	Intercellular adhesion molecule 1	2.44	7.04	4.74	
P30837	Aldehyde dehydrogenase X	6.29	3.12	4.71	
Q96S38	Ribosomal protein S6 kinase delta-1	8.15	1.18	4.66	
Q53GA4	Pleckstrin homology-like domain family A member 2	7.21	2.11	4.66	
Q27J81	Inverted formin-2	7.73	1.53	4.63	
Q9BWH6	RNA polymerase II-associated protein 1	8.74	0.50	4.62	
O96000	NADH dehydrogenase [ubiquinone] 1 beta subcomplex subunit 10	5.46	3.73	4.60	
P48047	ATP synthase subunit O	5.14	4.01	4.57	
Q9NP64	Nucleolar protein of 40 kDa	0.77	8.32	4.54	
P61163	Alpha-centractin	0.07	9.00	4.53	a
Q9Y605	MORF4 family-associated protein 1	6.19	2.87	4.53	
O43399	Tumor protein D54	3.37	5.67	4.52	a
Q86UE4	Protein LYRIC	3.81	5.22	4.51	
P61586	Transforming protein RhoA	2.56	6.46	4.51	
Q9GZT3	SRA stem-loop-interacting RNA-binding protein	4.67	4.28	4.47	
P62070	Ras-related protein R-Ras2	4.54	4.40	4.47	a
Q7Z6M4	Transcription termination factor 4	1.11	7.79	4.45	
O00264	Membrane-associated progesterone receptor component 1	4.61	4.27	4.44	
P31153	S-adenosylmethionine synthase isoform type-2	7.44	1.41	4.43	
Q9UNF1	Melanoma-associated antigen D2	6.01	2.83	4.42	
Q9UJZ1	Stomatin-like protein 2	5.05	3.65	4.35	a
P19525	Interferon-induced, double-stranded RNA-activated protein kinase	6.47	2.20	4.34	
O43396	Thioredoxin-like protein 1	7.82	0.84	4.33	
Q9UG56	Phosphatidylserine decarboxylase proenzyme	5.01	3.63	4.32	
Q5T0W9	Protein FAM83B	3.12	5.52	4.32	
P61006	Ras-related protein Rab-8A	4.25	4.38	4.32	
Q9NW81	ATP synthase subunit s-like protein	2.96	5.67	4.31	
P02795	Metallothionein-2	6.39	2.22	4.31	a
O75880	Protein SCO1 homolog	5.14	3.47	4.30	
P13073	Cytochrome c oxidase subunit 4 isoform 1	4.15	4.42	4.29	
Q9NQE9	Histidine triad nucleotide-binding protein 3	3.16	5.36	4.26	
Q6NYC8	Phostensin	6.19	2.29	4.24	
Q9Y3L3	SH3 domain-binding protein 1	3.86	4.61	4.24	
O43504	Ragulator complex protein LAMTOR5	6.24	2.22	4.23	
P60510	Serine/threonine-protein phosphatase 4 catalytic subunit	3.09	5.34	4.22	
P13804	Electron transfer flavoprotein subunit alpha	3.18	5.23	4.21	
Q9Y4Y9	U6 snRNA-associated Sm-like protein LSm5	7.06	1.36	4.21	
P25705	ATP synthase subunit alpha	4.66	3.75	4.20	
O95229	ZW10 interactor	6.10	2.17	4.14	
P78347	General transcription factor II-I	6.72	1.51	4.12	
Q8IWB9	Testis-expressed sequence 2 protein	6.70	1.49	4.10	
P40939	Trifunctional enzyme subunit alpha	4.72	3.43	4.08	a

Q6ZSJ8	Uncharacterized protein C1orf122	4.52	3.59	4.05	
O43291	Kunitz-type protease inhibitor 2	5.66	2.44	4.05	
Q96AG4	Leucine-rich repeat-containing protein 59	4.16	3.92	4.04	
O15031	Plexin-B2	6.01	2.02	4.02	a
P04899	Guanine nucleotide-binding protein G(i) subunit alpha-2	5.13	2.86	4.00	
P08574	Cytochrome c1, heme protein	3.11	4.87	3.99	
Q12962	Transcription initiation factor TFIID subunit 10	6.07	1.87	3.97	
P39656	Dolichyl-diphosphooligosaccharide--protein glycosyltransferase 48 kDa subunit	4.21	3.72	3.96	a
Q8IW35	Centrosomal protein of 97 kDa	7.04	0.88	3.96	
Q9P2B2	Prostaglandin F2 receptor negative regulator	5.32	2.59	3.96	
P63220	40S ribosomal protein S21	4.83	3.08	3.96	
Q9Y4W6	AFG3-like protein 2	5.07	2.84	3.96	
Q6PIU2	Neutral cholesterol ester hydrolase 1	4.05	3.83	3.94	
Q9UKX7	Nuclear pore complex protein Nup50	6.81	0.89	3.85	
O15269	Serine palmitoyltransferase 1	4.23	3.45	3.84	
O95721	Synaptosomal-associated protein 29	3.48	4.19	3.83	
Q9NPQ8	Synembryn-A	3.83	3.82	3.82	
Q9UJA5	tRNA (adenine(58)-N(1))-methyltransferase non-catalytic subunit TRM6	5.99	1.64	3.81	
Q9H936	Mitochondrial glutamate carrier 1	4.86	2.74	3.80	
O75955	Flotillin-1	5.64	1.92	3.78	a
P55084	Trifunctional enzyme subunit beta	3.91	3.64	3.78	a
P27482	Calmodulin-like protein 3	6.00	1.54	3.77	
Q99439	Calponin-2	6.82	0.72	3.77	
P13726	Tissue factor	3.96	3.58	3.77	a
P50454	Serpin H1	4.11	3.42	3.76	a
Q9UK45	U6 snRNA-associated Sm-like protein LSm7	5.40	2.10	3.75	
Q9BSQ5	Cerebral cavernous malformations 2 protein	7.17	0.32	3.74	
P14854	Cytochrome c oxidase subunit 6B1	4.53	2.91	3.72	
P04183	Thymidine kinase	6.03	1.37	3.70	
Q5D862	Filaggrin-2	5.49	1.91	3.70	
Q6ZVH7	Espin-like protein	1.80	5.59	3.70	
Q9NX20	39S ribosomal protein L16	6.35	1.04	3.69	
Q8WUF5	RelA-associated inhibitor	5.98	1.39	3.69	
O60762	Dolichol-phosphate mannosyltransferase subunit 1	4.13	3.21	3.67	
P08134	Rho-related GTP-binding protein RhoC	3.62	3.71	3.67	
O00483	Cytochrome c oxidase subunit NDUFA4	3.68	3.60	3.64	
Q8WVV9	Heterogeneous nuclear ribonucleoprotein L-like	2.03	5.25	3.64	
Q15738	Sterol-4-alpha-carboxylate 3-dehydrogenase	4.02	3.25	3.64	
Q8NCH0	Carbohydrate sulfotransferase 14	3.51	3.75	3.63	
Q8NI60	Atypical kinase ADCK3	3.82	3.43	3.62	
A0FGR8	Extended synaptotagmin-2	5.24	1.97	3.60	
Q96FQ6	Protein S100-A16	3.68	3.50	3.59	
P62879	Guanine nucleotide-binding protein G(I)/G(S)/G(T) subunit beta-2	3.72	3.45	3.59	a
P25789	Proteasome subunit alpha type-4	1.25	5.92	3.58	
P61224	Ras-related protein Rap-1b	4.17	2.97	3.57	
Q12846	Syntaxin-4	4.54	2.59	3.56	
Q08AF3	Schlafen family member 5	6.21	0.91	3.56	
P62841	40S ribosomal protein S15	4.04	3.05	3.55	
Q13426	DNA repair protein XRCC4	1.82	5.27	3.54	
O43175	D-3-phosphoglycerate dehydrogenase	3.93	3.16	3.54	
O14957	Cytochrome b-c1 complex subunit 10	3.70	3.38	3.54	
P01111	GTPase NRas	3.65	3.36	3.51	a
P05141	ADP/ATP translocase 2	3.74	3.25	3.49	a
Q9NXV6	CDKN2A-interacting protein	5.38	1.57	3.48	
Q9H061	Transmembrane protein 126A	4.04	2.91	3.47	
P78545	ETS-related transcription factor Elf-3	5.95	0.94	3.45	
Q9Y2Q5	Ragulator complex protein LAMTOR2	4.30	2.58	3.44	
O14980	Exportin-1	2.46	4.41	3.44	
P08240	Signal recognition particle receptor subunit alpha	3.38	3.45	3.41	a
P06733	Alpha-enolase	1.90	4.90	3.40	a
P09758	Tumor-associated calcium signal transducer 2	4.32	2.46	3.39	a
Q9NV56	MRG/MORF4L-binding protein	1.28	5.45	3.37	
P51149	Ras-related protein Rab-7a	3.71	3.00	3.36	
P10599	Thioredoxin	6.14	0.55	3.35	
Q9BTU6	Phosphatidylinositol 4-kinase type 2-alpha	1.62	5.07	3.34	
Q9BVJ7	Dual specificity protein phosphatase 23	3.65	3.03	3.34	
Q8NFH5	Nucleoporin NUP53	3.48	3.20	3.34	
P08865	40S ribosomal protein SA	3.97	2.66	3.31	
Q13151	Heterogeneous nuclear ribonucleoprotein A0	0.94	5.68	3.31	

Q99959	Plakophilin-2	5.51	1.09	3.30	a
Q13098	COP9 signalosome complex subunit 1	2.68	3.92	3.30	
O75489	NADH dehydrogenase [ubiquinone] iron-sulfur protein 3	3.32	3.28	3.30	
P49821	NADH dehydrogenase [ubiquinone] flavoprotein 1	3.57	2.99	3.28	
P18031	Tyrosine-protein phosphatase non-receptor type 1	2.68	3.86	3.27	
P09038	Fibroblast growth factor 2	0.92	5.61	3.26	
P78316	Nucleolar protein 14	6.08	0.41	3.25	
Q8WVX9	Fatty acyl-CoA reductase 1	3.92	2.54	3.23	
P28331	NADH-ubiquinone oxidoreductase 75 kDa subunit	3.77	2.66	3.22	
Q15390	Mitochondrial fission regulator 1	3.44	2.99	3.22	
Q9Y6J9	TAF6-like RNA polymerase II p300/CBP-associated factor-associated factor 65 kDa subunit 6L	0.31	6.11	3.21	
O15164	Transcription intermediary factor 1-alpha	3.32	3.08	3.20	
P61019	Ras-related protein Rab-2A	3.75	2.61	3.18	a
Q9Y3B4	Splicing factor 3B subunit 6	3.39	2.92	3.16	
O14964	Hepatocyte growth factor-regulated tyrosine kinase substrate	4.15	2.15	3.15	
P04632	Calpain small subunit 1	4.17	2.13	3.15	a
P62834	Ras-related protein Rap-1A	3.48	2.79	3.14	
P33316	Deoxyuridine 5'-triphosphate nucleotidohydrolase	3.54	2.73	3.14	
Q9UJS0	Calcium-binding mitochondrial carrier protein Aralar2	3.96	2.31	3.13	
P61769	Beta-2-microglobulin	3.37	2.88	3.12	
Q9H6Y2	WD repeat-containing protein 55	3.54	2.69	3.11	a
Q9UPN9	E3 ubiquitin-protein ligase TRIM33	4.03	2.18	3.10	
Q96E14	RecQ-mediated genome instability protein 2	1.53	4.66	3.10	
Q13049	E3 ubiquitin-protein ligase TRIM32	3.48	2.71	3.10	
O00418	Eukaryotic elongation factor 2 kinase	3.98	2.20	3.09	
O60664	Perilipin-3	3.71	2.47	3.09	a
P22234	Multifunctional protein ADE2	1.84	4.34	3.09	
Q13724	Mannosyl-oligosaccharide glucosidase	2.74	3.43	3.08	
O75438	NADH dehydrogenase [ubiquinone] 1 beta subcomplex subunit 1	3.37	2.79	3.08	
Q00403	Transcription initiation factor IIB	2.29	3.86	3.07	
Q96EV2	RNA-binding protein 33	1.06	5.07	3.07	
O00159	Unconventional myosin-Ic	5.12	0.99	3.05	
P54709	Sodium/potassium-transporting ATPase subunit beta-3	3.89	2.21	3.05	a
P24666	Low molecular weight phosphotyrosine protein phosphatase	5.28	0.79	3.04	
Q16718	NADH dehydrogenase [ubiquinone] 1 alpha subcomplex subunit 5	3.44	2.62	3.03	
Q96EY4	Translation machinery-associated protein 16	0.97	5.08	3.03	
P62273	40S ribosomal protein S29	3.16	2.88	3.02	
Q9BQG0	Myb-binding protein 1A	3.86	2.16	3.01	
Q9BPX6	Calcium uptake protein 1	3.40	2.60	3.00	
Q9UN37	Vacuolar protein sorting-associated protein 4A	3.31	2.69	3.00	
O00217	NADH dehydrogenase [ubiquinone] iron-sulfur protein 8	3.19	2.79	2.99	
Q3ZCQ8	Mitochondrial import inner membrane translocase subunit TIM50	3.34	2.60	2.97	
Q8N8A6	ATP-dependent RNA helicase DDX51	5.49	0.43	2.96	
Q99747	Gamma-soluble NSF attachment protein	2.90	3.01	2.95	
P62277	40S ribosomal protein S13	3.11	2.73	2.92	
O43678	NADH dehydrogenase [ubiquinone] 1 alpha subcomplex subunit 2	3.36	2.44	2.90	
Q5T9A4	ATPase family AAA domain-containing protein 3B	3.02	2.74	2.88	
Q9Y285	Phenylalanine--tRNA ligase alpha subunit	3.07	2.68	2.88	
P09497	Clathrin light chain B	0.87	4.86	2.87	
O14681	Etoposide-induced protein 2.4 homolog	3.28	2.45	2.86	
O14548	Cytochrome c oxidase subunit 7A-related protein	1.71	4.01	2.86	
Q99497	Protein deglycase DJ-1	1.33	4.38	2.85	
P04844	Dolichyl-diphosphooligosaccharide--protein glycosyltransferase subunit 2	1.10	4.57	2.84	
Q96A65	Exocyst complex component 4	3.13	2.52	2.83	
Q8WUH6	Transmembrane protein 263	3.22	2.39	2.81	
Q68CP9	AT-rich interactive domain-containing protein 2	3.28	2.33	2.80	
O43920	NADH dehydrogenase [ubiquinone] iron-sulfur protein 5	2.75	2.85	2.80	
P19404	NADH dehydrogenase [ubiquinone] flavoprotein 2	3.45	2.15	2.80	
Q9BUQ8	Probable ATP-dependent RNA helicase DDX23	5.45	0.14	2.80	
O75380	NADH dehydrogenase [ubiquinone] iron-sulfur protein 6	3.47	2.12	2.80	
Q96IZ0	PRKC apoptosis WT1 regulator protein	4.79	0.72	2.75	
Q13425	Beta-2-syntrophin	2.32	3.18	2.75	
Q9HCU5	Prolactin regulatory element-binding protein	3.14	2.36	2.75	
P17812	CTP synthase 1	2.63	2.85	2.74	
P30153	Serine/threonine-protein phosphatase 2A 65 kDa regulatory subunit A alpha isoform	4.22	1.27	2.74	a
Q9P287	BRCA2 and CDKN1A-interacting protein	3.10	2.34	2.72	
Q9H1A4	Anaphase-promoting complex subunit 1	0.33	5.11	2.72	

P35268	60S ribosomal protein L22	3.29	2.13	2.71	
Q9Y6N5	Sulfide:quinone oxidoreductase	3.30	2.11	2.70	
P55196	Afadin	3.52	1.84	2.68	a
Q5JTZ9	Alanine--tRNA ligase	2.61	2.74	2.67	
Q16342	Programmed cell death protein 2	3.15	2.19	2.67	
P78527	DNA-dependent protein kinase catalytic subunit	3.39	1.95	2.67	
Q14680	Maternal embryonic leucine zipper kinase	1.12	4.21	2.67	
O76094	Signal recognition particle subunit SRP72	3.18	2.15	2.67	
P52943	Cysteine-rich protein 2	3.37	1.93	2.65	
Q6PJF5	Inactive rhomboid protein 2	3.13	2.16	2.64	
P10321	HLA class I histocompatibility antigen, Cw-7 alpha chain	2.03	3.21	2.62	a
P56182	Ribosomal RNA processing protein 1 homolog A	2.89	2.32	2.61	
P61026	Ras-related protein Rab-10	1.95	3.22	2.59	
Q9NWB7	Intraflagellar transport protein 57 homolog	5.04	0.11	2.58	
Q9NSI2	Protein FAM207A	3.48	1.67	2.58	
P46459	Vesicle-fusing ATPase	2.83	2.28	2.55	
Q8WWI1	LIM domain only protein 7	2.59	2.50	2.54	
P35080	Profilin-2	2.66	2.43	2.54	
Q52LJ0	Protein FAM98B	2.48	2.57	2.53	
O14579	Coatomer subunit epsilon	3.05	1.99	2.52	a
Q9Y6D0	Selenoprotein K	2.72	2.30	2.51	
P61289	Proteasome activator complex subunit 3	2.57	2.44	2.50	
Q96FX7	tRNA (adenine(58)-N(1))-methyltransferase catalytic subunit TRMT61A	2.87	2.13	2.50	
Q9H4L7	SWI/SNF-related matrix-associated actin-dependent regulator of chromatin subfamily A containing DEAD/H box 1	2.49	2.51	2.50	
O00193	Small acidic protein	3.06	1.94	2.50	
P62269	40S ribosomal protein S18	2.72	2.26	2.49	
Q5JTH9	RRP12-like protein	1.84	3.13	2.49	
P02786	Transferrin receptor protein 1	2.74	2.21	2.48	
Q9H6Z4	Ran-binding protein 3	2.82	2.13	2.47	
P46783	40S ribosomal protein S10	2.72	2.23	2.47	
O14519	Cyclin-dependent kinase 2-associated protein 1	2.84	2.11	2.47	
Q07065	Cytoskeleton-associated protein 4	2.92	2.03	2.47	a
Q9Y3T9	Nucleolar complex protein 2 homolog	3.86	1.08	2.47	
P98170	E3 ubiquitin-protein ligase XIAP	2.58	2.34	2.46	
Q9BPX3	Condensin complex subunit 3	2.83	2.07	2.45	
P26006	Integrin alpha-3	2.54	2.35	2.44	
Q92522	Histone H1x	2.68	2.20	2.44	
Q14738	Serine/threonine-protein phosphatase 2A 56 kDa regulatory subunit delta	2.20	2.66	2.43	
P34897	Serine hydroxymethyltransferase	2.49	2.37	2.43	
Q14847	LIM and SH3 domain protein 1	2.78	2.07	2.42	
P46109	Crk-like protein	3.11	1.74	2.42	
P35659	Protein DEK	2.70	2.14	2.42	
O75746	Calcium-binding mitochondrial carrier protein Aralar1	2.75	2.08	2.42	
Q14318	Peptidyl-prolyl cis-trans isomerase FKBP8	2.88	1.95	2.41	
P00533	Epidermal growth factor receptor	2.91	1.91	2.41	a
O95182	NADH dehydrogenase [ubiquinone] 1 alpha subcomplex subunit 7	2.99	1.79	2.39	
Q86WX3	Active regulator of SIRT1	2.78	2.00	2.39	
P63151	Serine/threonine-protein phosphatase 2A 55 kDa regulatory subunit B alpha isoform	2.72	2.05	2.39	
Q9Y4P3	Transducin beta-like protein 2	2.92	1.83	2.38	
P26373	60S ribosomal protein L13	2.79	1.95	2.37	a
P22223	Cadherin-3	0.30	4.44	2.37	
P41208	Centrin-2	2.44	2.29	2.36	
P61254	60S ribosomal protein L26	2.43	2.28	2.36	
Q14671	Pumilio homolog 1	2.86	1.84	2.35	
Q96TA1	Niban-like protein 1	1.01	3.69	2.35	a
P49790	Nuclear pore complex protein Nup153	2.78	1.91	2.34	
Q9H0B6	Kinesin light chain 2	2.92	1.74	2.33	
Q9NQU5	Serine/threonine-protein kinase PAK 6	3.24	1.41	2.33	
Q8IXK0	Polyhomeotic-like protein 2	0.32	4.30	2.31	
Q86YZ3	Hornerin	3.30	1.30	2.30	
Q9Y5M8	Signal recognition particle receptor subunit beta	2.51	2.09	2.30	
Q8TBA6	Golgin subfamily A member 5	2.59	1.97	2.28	
Q9BR61	Acyl-CoA-binding domain-containing protein 6	2.74	1.81	2.27	
P04732	Metallothionein-1E	2.47	2.05	2.26	
O43395	U4/U6 small nuclear ribonucleoprotein Prp3	4.16	0.35	2.26	
Q9Y5P4	Collagen type IV alpha-3-binding protein	2.30	2.20	2.25	
P08195	4F2 cell-surface antigen heavy chain	2.71	1.79	2.25	a

P63173	60S ribosomal protein L38	2.69	1.80	2.24	
P56962	Syntaxin-17	2.69	1.80	2.24	
P24752	Acetyl-CoA acetyltransferase	2.63	1.85	2.24	
Q9Y508	E3 ubiquitin-protein ligase RNF114	1.61	2.85	2.23	
Q9UPR3	Protein SMG5	2.65	1.81	2.23	
P51970	NADH dehydrogenase [ubiquinone] 1 alpha subcomplex subunit 8	2.48	1.98	2.23	
P46781	40S ribosomal protein S9	2.48	1.97	2.22	
Q3B7T1	Erythroid differentiation-related factor 1	3.04	1.39	2.22	
P10809	60 kDa heat shock protein	2.62	1.81	2.21	a
Q9H9Q2	COP9 signalosome complex subunit 7b	2.91	1.51	2.21	
P04406	Glyceraldehyde-3-phosphate dehydrogenase	2.54	1.87	2.20	
Q14204	Cytoplasmic dynein 1 heavy chain 1	2.79	1.57	2.18	
Q9H3K6	BolA-like protein 2	2.78	1.56	2.17	a
Q9NT62	Ubiquitin-like-conjugating enzyme ATG3	2.53	1.80	2.17	
Q9HBH1	Peptide deformylase	2.58	1.75	2.17	
P00403	Cytochrome c oxidase subunit 2	2.72	1.60	2.16	
P61011	Signal recognition particle 54 kDa protein	2.34	1.97	2.16	
Q9UBX3	Mitochondrial dicarboxylate carrier	2.20	2.11	2.16	
P62937	Peptidyl-prolyl cis-trans isomerase A	2.54	1.76	2.15	a
P23528	Cofilin-1	2.59	1.70	2.14	
Q9UNS2	COP9 signalosome complex subunit 3	2.24	2.03	2.13	
P35606	Coatomer subunit beta'	1.80	2.46	2.13	
Q5UIP0	Telomere-associated protein RIF1	2.69	1.55	2.12	
Q8WXC6	Myeloma-overexpressed gene 2 protein	2.28	1.95	2.11	
P14927	Cytochrome b-c1 complex subunit 7	2.45	1.76	2.11	
P25686	DnaJ homolog subfamily B member 2	3.11	1.02	2.07	
Q5JTV8	Torsin-1A-interacting protein 1	2.58	1.55	2.07	
Q14126	Desmoglein-2	2.56	1.56	2.06	a
Q96SY0	von Willebrand factor A domain-containing protein 9	1.68	2.44	2.06	
P31930	Cytochrome b-c1 complex subunit 1	2.26	1.84	2.05	
Q9UHB9	Signal recognition particle subunit SRP68	2.35	1.75	2.05	
P04439	HLA class I histocompatibility antigen, A-3 alpha chain	2.01	2.09	2.05	
P51858	Hepatoma-derived growth factor	2.33	1.75	2.04	
P17275	Transcription factor jun-B	2.58	1.49	2.03	
Q9NRF9	DNA polymerase epsilon subunit 3	2.92	1.13	2.03	
Q9Y3D0	Mitotic spindle-associated MMXD complex subunit MIP18	2.62	1.43	2.03	
P06753	Tropomyosin alpha-3 chain	2.48	1.54	2.01	
P57740	Nuclear pore complex protein Nup107	2.39	1.59	1.99	
Q9BSD7	Cancer-related nucleoside-triphosphatase	2.36	1.61	1.98	
P61923	Coatomer subunit zeta-1	2.29	1.68	1.98	a
Q96HQ2	CDKN2AIP N-terminal-like protein	2.62	1.33	1.98	
Q02413	Desmoglein-1	2.35	1.57	1.96	
Q9UHB6	LIM domain and actin-binding protein 1	2.36	1.55	1.96	
Q9UI30	Multifunctional methyltransferase subunit TRM112-like protein	2.21	1.69	1.95	
P11279	Lysosome-associated membrane glycoprotein 1	2.57	1.33	1.95	
Q96CB8	Integrator complex subunit 12	2.10	1.80	1.95	
Q9UK22	F-box only protein 2	1.75	2.14	1.95	
Q9BWF3	RNA-binding protein 4	2.69	1.20	1.94	
Q16527	Cysteine and glycine-rich protein 2	2.68	1.20	1.94	
O60888	Protein CutA	3.26	0.61	1.93	
P16401	Histone H1.5	2.68	1.16	1.92	
P69905	Hemoglobin subunit alpha	2.53	1.29	1.91	
Q03252	Lamin-B2	2.36	1.44	1.90	
Q99442	Translocation protein SEC62	2.47	1.33	1.90	
P84098	60S ribosomal protein L19	2.36	1.44	1.90	
P15954	Cytochrome c oxidase subunit 7C	3.59	0.21	1.90	
Q99627	COP9 signalosome complex subunit 8	2.01	1.78	1.90	
Q9H5X1	MIP18 family protein FAM96A	1.70	2.04	1.87	
Q9Y2U8	Inner nuclear membrane protein Man1	2.04	1.68	1.86	
O60613	15 kDa selenoprotein	2.25	1.47	1.86	
P16403	Histone H1.2	2.28	1.43	1.85	
P62899	60S ribosomal protein L31	2.35	1.34	1.85	
Q86V81	THO complex subunit 4	2.12	1.57	1.85	
P46776	60S ribosomal protein L27a	2.12	1.56	1.84	
Q00610	Clathrin heavy chain 1	1.54	2.12	1.83	a
Q9Y4E8	Ubiquitin carboxyl-terminal hydrolase 15	2.37	1.27	1.82	
Q9H3U1	Protein unc-45 homolog A	2.36	1.27	1.82	
P35249	Replication factor C subunit 4	2.02	1.61	1.81	
P13667	Protein disulfide-isomerase A4	2.16	1.42	1.79	

Q9NVH0	Exonuclease 3'-5' domain-containing protein 2	2.14	1.43	1.79	
Q8TEX9	Importin-4	1.40	2.16	1.78	
O60248	Protein SOX-15	2.52	1.04	1.78	
Q9BSV6	tRNA-splicing endonuclease subunit Sen34	2.73	0.79	1.76	
P61201	COP9 signalosome complex subunit 2	2.12	1.41	1.76	
P07339	Cathepsin D	0.61	2.89	1.75	a
P04843	Dolichyl-diphosphooligosaccharide--protein glycosyltransferase subunit 1	2.11	1.38	1.75	
P32119	Peroxiredoxin-2	2.14	1.35	1.75	
Q8IXI1	Mitochondrial Rho GTPase 2	2.02	1.45	1.74	
Q12905	Interleukin enhancer-binding factor 2	2.21	1.26	1.74	
O94901	SUN domain-containing protein 1	2.20	1.27	1.74	a
Q5T8D3	Acyl-CoA-binding domain-containing protein 5	2.12	1.35	1.73	
Q15149	Plectin	2.11	1.34	1.73	
Q9BSJ8	Extended synaptotagmin-1	2.77	0.67	1.72	
Q14134	Tripartite motif-containing protein 29	2.14	1.27	1.70	
P53701	Cytochrome c-type heme lyase	2.49	0.89	1.69	
Q8IUF1	COBW domain-containing protein 2	2.34	1.03	1.69	
P09012	U1 small nuclear ribonucleoprotein A	2.25	1.12	1.68	
P28799	Granulins	2.25	1.11	1.68	a
Q53HL2	Borealin	1.02	2.32	1.67	
P04792	Heat shock protein beta-1	2.34	0.99	1.67	
Q9BT78	COP9 signalosome complex subunit 4	2.19	1.13	1.66	
Q9H3Q1	Cdc42 effector protein 4	2.27	1.05	1.66	
Q92905	COP9 signalosome complex subunit 5	2.26	1.06	1.66	
P46782	40S ribosomal protein S5	2.04	1.28	1.66	
Q86X53	Glutamate-rich protein 1	2.07	1.24	1.65	
Q8N6N3	UPF0690 protein C1orf52	0.93	2.30	1.61	
P78318	Immunoglobulin-binding protein 1	2.01	1.22	1.61	
O60716	Catenin delta-1	2.11	1.11	1.61	a
P07919	Cytochrome b-c1 complex subunit 6	2.05	1.16	1.60	
Q9NRG0	Chromatin accessibility complex protein 1	2.14	1.00	1.57	
P16383	GC-rich sequence DNA-binding factor 2	2.44	0.71	1.57	
P21291	Cysteine and glycine-rich protein 1	2.17	0.96	1.57	
O15162	Phospholipid scramblase 1	2.03	1.10	1.56	
O95361	Tripartite motif-containing protein 16	2.42	0.69	1.56	
P61513	60S ribosomal protein L37a	2.10	1.01	1.55	
Q99714	3-hydroxyacyl-CoA dehydrogenase type-2	2.09	1.02	1.55	
P54105	Methylosome subunit pICln	2.05	1.05	1.55	
P28066	Proteasome subunit alpha type-5	2.06	1.04	1.55	
O00192	Armadillo repeat protein deleted in velo-cardio-facial syndrome	2.00	1.00	1.50	
Q9H1B7	Interferon regulatory factor 2-binding protein-like	2.18	0.82	1.50	
P20962	Parathymosin	2.04	0.96	1.50	
Q5H9R7	Serine/threonine-protein phosphatase 6 regulatory subunit 3	2.16	0.77	1.47	
P07355	Annexin A2	2.00	0.93	1.47	
Q93008	Probable ubiquitin carboxyl-terminal hydrolase FAF-X	2.08	0.81	1.45	
Q8IYL3	UPF0688 protein C1orf174	2.03	0.80	1.42	
Q8N5G2	Macoilin	2.30	0.51	1.41	
O00487	26S proteasome non-ATPase regulatory subunit 14	2.73	0.04	1.39	a
Q9H6B4	CXADR-like membrane protein	2.31	0.38	1.35	
O00488	Zinc finger protein 593	2.09	0.60	1.35	
Q9Y4X5	E3 ubiquitin-protein ligase ARIH1	2.00	0.63	1.31	
P51665	26S proteasome non-ATPase regulatory subunit 7	2.09	0.47	1.28	
Q96T76	MMS19 nucleotide excision repair protein homolog	2.06	0.41	1.24	
P09543	2',3'-cyclic-nucleotide 3'-phosphodiesterase	2.03	0.42	1.22	
P30084	Enoyl-CoA hydratase	2.32	0.07	1.19	
P52655	Transcription initiation factor IIA subunit 1	0.17	2.04	1.10	

Table S7. Quantitation of reduced Met-peptides in the 12 samples

MS data from the label-swap replicated SILAC experiments (Exp.1, PDT/Ctrl = Light/Heavy; Exp. 2, PDT/Ctrl = Heavy/Light) described in Table S1 were used to analyze the reduced Met-peptides that were detected/quantified in the non-enriched and enriched samples. The numbers shown in the intersecting regions of the blue and red circles denote the reduced Met-peptides that were commonly detected/quantified in both enriched and non-enriched samples.

Peptide identification	Condition I				Condition II				Condition III			
	H=Ctrl, L=PDT		H=PDT, L=Ctrl		H=Ctrl, L=PDT		H=PDT, L=Ctrl		H=Ctrl, L=PDT		H=PDT, L=Ctrl	
	Non-enriched	Enriched	Non-enriched	Enriched	Non-enriched	Enriched	Non-enriched	Enriched	Non-enriched	Enriched	Non-enriched	Enriched
No. of reduced Met-peptides	4361	3654	6333	3816	5851	8504	5371	5273	5903	4315	4927	5183
Identified in both protocols	2195		2521		4151		2953		2691		2843	
Identified in one protocol only	2166	1459	3812	1295	1700	4353	2418	2320	3212	1624	2084	2340
	2166 2195 1459		3812 2521 1295		1700 4151 4353		2418 2953 2320		3212 2691 1624		2084 2843 2340	
No. of significantly changed reduced Met-peptides	301	454	317	215	343	391	255	224	251	287	263	338
Quantified in both protocols	73		48		37		18		76		107	
Quantified in one protocol only	228	381	269	167	306	354	237	206	175	211	156	231
	228 73 381		269 48 167		306 37 354		237 18 206		175 76 211		156 107 231	

Table S8. A cross-study comparison of oxidative stress-sensitive proteins.

The list shows the severely oxidized proteins identified in the present study and other studies (Ref. No. 1-7).

Accession	Protein name	Oxidative treatment		Oxidation sensitive proteins	Identified in this study	ER, Golgi and mitochondrion distribution
		PDT	H_2O_2 and others			
O00116	Alkyldihydroxyacetonephosphate synthase	3				Mito
O00151	PDZ and LIM domain protein 1	3				
O00264	Membrane-associated progesterone receptor component 1	2		PDT specific		ER
O00299	Chloride intracellular channel protein 1	3				Mito
O00410	Importin-5				Y	Golgi
O00425	Insulin-like growth factor 2 mRNA-binding protein 3	3				
O00487	26S proteasome non-ATPase regulatory subunit 14		5		Y	
O00571	ATP-dependent RNA helicase DDX3X	3				Mito
O14579	Coatomer subunit epsilon				Y	ER, Golgi
O14744	Protein arginine N-methyltransferase 5				Y	Golgi
O14745	Na(+)/H(+) exchange regulatory cofactor NHE-RF1	3				
O15031	Plexin-B2				Y	
O15160	DNA-directed RNA polymerases I and III subunit RPAC1				Y	
O15173	Membrane-associated progesterone receptor component 2				Y	
O15260	Surfeit locus protein 4		5		Y	ER, Golgi
O15270	Serine palmitoyltransferase 2				Y	ER, Mito
O15305	Phosphomannomutase 2	3				
O15355	Protein phosphatase 1G	3				
O15371	Eukaryotic translation initiation factor 3 subunit D				Y	
O15372	Eukaryotic translation initiation factor 3 subunit H				Y	
O15460	Prolyl 4-hydroxylase subunit alpha-2	3				ER
O43143	Pre-mRNA-splicing factor ATP-dependent RNA helicase DHX15	3				
O43148	mRNA cap guanine-N7 methyltransferase	3				
O43172	U4/U6 small nuclear ribonucleoprotein Prp4	3				
O43175	D-3-phosphoglycerate dehydrogenase	3				
O43390	Heterogeneous nuclear ribonucleoprotein R				Y	ER
O43399	Tumor protein D54				Y	
O43615	Mitochondrial import inner membrane translocase subunit TIM44		5			Mito
O43707	Alpha-actinin-4	3	5	Sensitive	Y	
O43752	Syntaxin-6				Y	Golgi
O43795	Unconventional myosin-Ib	3				
O43852	Calumenin				Y	ER, Golgi
O60568	Procollagen-lysine,2-oxoglutarate 5-dioxygenase 3	3				ER
O60610	Protein diaphanous homolog 1		5			
O60664	Perilipin-3				Y	Golgi
O60716	Catenin delta-1				Y	
O60763	General vesicular transport factor p115				Y	ER, Golgi
O60814	Histone H2B type 1-K				Y	
O75083	WD repeat-containing protein 1	3				
O75116	Rho-associated protein kinase 2				Y	
O75131	Copine-3				Y	
O75369	Filamin-B	3	4, 5	Sensitive		
O75390	Citrate synthase	3				Mito
O75396	Vesicle-trafficking protein SEC22b		5		Y	ER, Golgi
O75436	Vacuolar protein sorting-associated protein 26A				Y	
O75533	Splicing factor 3B subunit 1	3				
O75534	Cold shock domain-containing protein E1	2		PDT specific	Y	Golgi, Mito
O75694	Nuclear pore complex protein Nup155				Y	
O75794	Cell division cycle protein 123 homolog	3				
O75821	Eukaryotic translation initiation factor 3 subunit G	3				
O75844	CAAX prenyl protease 1 homolog				Y	ER
O75874	Isocitrate dehydrogenase [NADP]	3				Mito
O75915	PRA1 family protein 3				Y	ER
O75955	Flotillin-1				Y	
O76031	ATP-dependent Clp protease ATP-binding subunit clpX-like	3				Mito
O94826	Mitochondrial import receptor subunit TOM70				Y	Mito
O94901	SUN domain-containing protein 1				Y	
O95169	NADH dehydrogenase [ubiquinone] 1 beta subcomplex subunit 8				Y	ER, Mito
O95347	Structural maintenance of chromosomes protein 2	3				
O95373	Importin-7				Y	
O95394	Phosphoacetylglucosamine mutase				Y	

O95433	Activator of 90 kDa heat shock protein ATPase homolog 1	3				ER
O96008	Mitochondrial import receptor subunit TOM40 homolog				Y	Mito
O96019	Actin-like protein 6A				Y	
P00338	L-lactate dehydrogenase A chain	3			Y	
P00367	Glutamate dehydrogenase 1	3				Mito
P00387	NADH-cytochrome b5 reductase 3				Y	ER, Mito
P00390	Glutathione reductase	3				Mito
P00492	Hypoxanthine-guanine phosphoribosyltransferase	3				
P00505	Aspartate aminotransferase	3				Mito
P00533	Epidermal growth factor receptor	3		PDT specific	Y	ER, Golgi
P00558	Phosphoglycerate kinase 1	3	4	Sensitive	Y	
P00918	Carbonic anhydrase 2	3				
P00966	Argininosuccinate synthase				Y	ER, Mito
P01111	GTPase Nras				Y	Golgi
P02545	Lamin-A/C		4		Y	
P02786	Transferrin receptor protein 1	3				
P02795	Metallothionein-2				Y	
P04040	Catalase	3				ER, Golgi, Mito
P04075	Fructose-bisphosphate aldolase A	3				
P04083	Annexin A1	2, 3		PDT specific	Y	Mito
P04181	Ornithine aminotransferase	3				Mito
P04406	Glyceraldehyde-3-phosphate dehydrogenase	3				
P04632	Calpain small subunit 1				Y	
P05023	Sodium/potassium-transporting ATPase subunit alpha-1	3	5	Sensitive	Y	ER, Golgi
P05026	Sodium/potassium-transporting ATPase subunit beta-1				Y	
P05141	ADP/ATP translocase 2				Y	Mito
P05165	Propionyl-CoA carboxylase alpha chain	3				Mito
P05198	Eukaryotic translation initiation factor 2 subunit 1				Y	
P05455	Lupus La protein	3				
P05556	Integrin beta-1	3	4	Sensitive	Y	
P06396	Gelsolin	3				
P06576	ATP synthase subunit beta	3		PDT specific	Y	Mito
P06733	Alpha-enolase	1, 3	4	Sensitive	Y	
P06737	Glycogen phosphorylase	3				
P06744	Glucose-6-phosphate isomerase	3				
P06748	Nucleophosmin	3				
P07108	Acyl-CoA-binding protein		5		Y	ER, Golgi
P07195	L-lactate dehydrogenase B chain		5		Y	Mito
P07237	Protein disulfide-isomerase	1, 3	5	Sensitive	Y	ER, Golgi
P07339	Cathepsin D		4		Y	
P07355	Annexin A2	3	4	Sensitive		
P07437	Tubulin beta chain		5		Y	
P07602	Prosaposin				Y	Mito
P07814	Bifunctional glutamate/proline--tRNA ligase	3				
P07900	Heat shock protein HSP 90-alpha	3	4	Sensitive	Y	Mito
P07954	Fumarate hydratase	3				Mito
P08107	Heat shock 70 kDa protein 1A/1B		4			
P08195	4F2 cell-surface antigen heavy chain	3		PDT specific	Y	
P08238	Heat shock protein HSP 90-beta	3		PDT specific	Y	Mito
P08240	Signal recognition particle receptor subunit alpha (SR-alpha)				Y	ER
P08708	40S ribosomal protein S17				Y	
P08758	Annexin A5				Y	
P09429	High mobility group protein B1		4			ER, Golgi
P09525	Annexin A4	3				
P09622	Dihydrolipoyl dehydrogenase	3		PDT specific		Mito
P09758	Tumor-associated calcium signal transducer 2				Y	
P09874	Poly [ADP-ribose] polymerase 1	3				Mito
P09960	Leukotriene A-4 hydrolase	3				
P0DMV8	Heat shock 70 kDa protein 1A	3				Mito
P10321	HLA class I histocompatibility antigen, Cw-7 alpha chain				Y	ER, Golgi
P10644	cAMP-dependent protein kinase type I-alpha regulatory subunit				Y	
P10809	60 kDa heat shock protein	3			Y	Mito
P11021	78 kDa glucose-regulated protein	1, 3		PDT specific	Y	ER, Golgi, Mito
P11142	Heat shock cognate 71 kDa protein	1, 3	4, 5, 7	Sensitive	Y	
P11177	Pyruvate dehydrogenase E1 component subunit beta	3				Mito
P11310	Medium-chain specific acyl-CoA dehydrogenase	3				Mito

P11413	Glucose-6-phosphate 1-dehydrogenase		4, 6			
P11498	Pyruvate carboxylase	3				Mito
P11586	C-1-tetrahydrofolate synthase	3				Mito
P11940	Polyadenylate-binding protein 1	3	4, 5	Sensitive	Y	
P12004	Proliferating cell nuclear antigen (PCNA)				Y	
P12081	Histidine--tRNA ligase	3				Mito
P12236	ADP/ATP translocase 3				Y	Mito
P12268	Inosine-5'-monophosphate dehydrogenase 2	3				
P12429	Annexin A3	3				
P12814	Alpha-actinin-1	3	4, 5	Sensitive	Y	
P12956	X-ray repair cross-complementing protein 6	3				
P13010	X-ray repair cross-complementing protein 5	3				
P13489	Ribonuclease inhibitor	3				
P13639	Elongation factor 2	3	4, 6	Sensitive	Y	
P13667	Protein disulfide-isomerase A4	3	5	Sensitive		ER
P13674	Prolyl 4-hydroxylase subunit alpha-1	3				ER, Mito
P13693	Translationally-controlled tumor protein				Y	
P13726	Tissue factor				Y	
P13796	Plastin-2	3	5	Sensitive	Y	
P13797	Plastin-3	3		PDT specific		
P13798	Acylamino-acid-releasing enzyme	3				
P13804	Electron transfer flavoprotein subunit alpha	2, 3	5	Sensitive		Mito
P13995	Bifunctional methylenetetrahydrofolate dehydrogenase/cyclohydrolase	3				Mito
P14314	Glucosidase 2 subunit beta	3	5	Sensitive	Y	ER
P14618	Pyruvate kinase isozymes M1/M2	3	4, 5	Sensitive		Mito
P14625	Endoplasmin	3	6		Y	ER
P14868	Aspartate--tRNA ligase	3				
P14923	Junction plakoglobin				Y	
P15311	Ezrin	3	6	Sensitive	Y	
P15559	NAD	3				
P15586	N-acetylglucosamine-6-sulfatase				Y	
P15880	40S ribosomal protein S2				Y	
P15924	Desmoplakin				Y	
P16070	CD44 antigen		4		Y	Golgi
P16083	Ribosyldihydronicotinamide dehydrogenase [quinone]	3				
P16144	Integrin beta-4	3		PDT specific	Y	
P16152	Carbonyl reductase [NADPH] 1	3				
P16422	Epithelial cell adhesion molecule				Y	
P16435	NADPH--cytochrome P450 reductase	3				ER, Mito
P16615	Sarcoplasmic/endoplasmic reticulum calcium ATPase 2	3				ER
P17174	Aspartate aminotransferase	3				
P17655	Calpain-2 catalytic subunit	3	4, 7	Sensitive		ER, Golgi
P17980	26S protease regulatory subunit 6A	3				
P17987	T-complex protein 1 subunit alpha	3	5, 6, 7	Sensitive	Y	Golgi
P18206	Vinculin	3			Y	
P18754	Regulator of chromosome condensation	3				
P19338	Nucleolin	3				
P19367	Hexokinase-1				Y	Mito
P20618	Proteasome subunit beta type-1	3				
P20645	Cation-dependent mannose-6-phosphate receptor				Y	Golgi
P20700	Lamin-B1		5		Y	
P21333	Filamin-A	3	4, 5, 7	Sensitive		
P21399	Cytoplasmic aconitate hydratase	3				ER, Golgi, Mito
P21796	Voltage-dependent anion-selective channel protein 1		4		Y	Mito
P22059	Oxysterol-binding protein 1				Y	ER, Golgi
P22061	Protein-L-isoaspartate(D-aspartate) O-methyltransferase	3		PDT specific	Y	
P22102	Trifunctional purine biosynthetic protein adenosine-3	3				
P22234	Multifunctional protein ADE2	3				
P22314	Ubiquitin-like modifier-activating enzyme 1	3				Mito
P22570	NADPH:adrenodoxin oxidoreductase	3				Mito
P22626	Heterogeneous nuclear ribonucleoproteins A2/B1		4, 5, 6		Y	
P22695	Cytochrome b-c1 complex subunit 2				Y	Mito
P23246	Splicing factor, proline- and glutamine-rich		5		Y	
P23284	Peptidyl-prolyl cis-trans isomerase B	3			Y	ER
P23368	NAD-dependent malic enzyme	3				Mito
P23526	Adenosylhomocysteinase	3				

P23588	Eukaryotic translation initiation factor 4B	3				
P23921	Ribonucleoside-diphosphate reductase large subunit	3				
P25398	40S ribosomal protein S12				Y	
P25705	ATP synthase subunit alpha	3				Mito
P25786	Proteasome subunit alpha type-1	3				
P25787	Proteasome subunit alpha type-2	3				
P25788	Proteasome subunit alpha type-3	3				
P25789	Proteasome subunit alpha type-4	3				
P26038	Moesin	3	5, 6	Sensitive	Y	
P26358	DNA (cytosine-5)-methyltransferase 1				Y	
P26373	60S ribosomal protein L13				Y	
P26639	Threonine--tRNA ligase	3				
P26640	Valine--tRNA ligase	3				Mito
P26641	Elongation factor 1-gamma		4, 5, 6		Y	
P27348	14-3-3 protein theta	3				Mito
P27361	Mitogen-activated protein kinase 3	3				Golgi, Mito
P27695	DNA-(apurinic or apyrimidinic site) lyase	3				ER, Mito
P27797	Calreticulin	1, 3	4	Sensitive		ER, Golgi
P27816	Microtubule-associated protein 4	3				
P27824	Calnexin	3		PDT specific		ER, Mito
P28070	Proteasome subunit beta type-4	3				
P28482	Mitogen-activated protein kinase 1				Y	Golgi, Mito
P28799	Granulins				Y	
P28838	Cytosol aminopeptidase	3				Golgi, Mito
P29144	Tripeptidyl-peptidase 2	3				
P29218	Inositol monophosphatase 1	3				
P29401	Transketolase	3				
P29692	Elongation factor 1-delta	3			Y	
P29966	Myristoylated alanine-rich C-kinase substrate	3		PDT specific		
P30040	Endoplasmic reticulum resident protein 29				Y	ER
P30041	Peroxiredoxin-6	3				
P30048	Thioredoxin-dependent peroxide reductase	2		PDT specific		Mito
P30101	Protein disulfide-isomerase A3	3	6	Sensitive		ER
P30153	Serine/threonine-protein phosphatase 2A 65 kDa regulatory subunit A	3			Y	Mito
P30419	Glycylpeptide N-tetradecanoyltransferase 1	3				Mito
P30740	Leukocyte elastase inhibitor (LEI)				Y	
P30837	Aldehyde dehydrogenase X	3				Mito
P31150	Rab GDP dissociation inhibitor alpha	3				Golgi
P31153	S-adenosylmethionine synthase isoform type-2	3				
P31689	DnaJ homolog subfamily A member 1				Y	ER, Mito
P31943	Heterogeneous nuclear ribonucleoprotein H		4, 5		Y	
P31947	14-3-3 protein sigma	3			Y	Mito
P31948	Stress-induced-phosphoprotein 1		4, 5			Golgi
P33121	Long-chain-fatty-acid--CoA ligase 1				Y	ER, Mito
P33176	Kinesin-1 heavy chain	3				
P33527	Multidrug resistance-associated protein 1				Y	
P33991	DNA replication licensing factor MCM4	3				
P33993	DNA replication licensing factor MCM7	3				
P34897	Serine hydroxymethyltransferase	3				Mito
P34932	Heat shock 70 kDa protein 4	3			Y	
P35221	Catenin alpha-1	3		PDT specific	Y	Golgi
P35241	Radixin	3				
P35579	Myosin-9	3	5, 7	Sensitive	Y	
P35606	Coatomer subunit beta'	3	5	Sensitive		ER, Golgi
P35613	Basigin				Y	Golgi, Mito
P35637	RNA-binding protein FUS (75 kDa DNA-pairing protein)	3			Y	
P35658	Nuclear pore complex protein Nup214				Y	
P35998	26S protease regulatory subunit 7	3		PDT specific	Y	
P36578	60S ribosomal protein L4		7			
P36952	Serpin B5				Y	
P36957	Dihydrolipoyllysine-residue succinyltransferase component of 2-oxoglutarate dehydrogenase complex	3				Mito
P37802	Transgelin-2	3	5	Sensitive	Y	
P37837	Transaldolase	3				
P38117	Electron transfer flavoprotein subunit beta	3				Mito
P38159	RNA-binding motif protein, X chromosome (Glycoprotein p43)				Y	

P38606	V-type proton ATPase catalytic subunit A	3				Mito
P38646	Stress-70 protein	3	5	Sensitive	Y	Mito
P39656	Dolichyl-diphosphooligosaccharide--protein glycosyltransferase 48 kDa				Y	ER
P40222	Alpha-taxilin		5			
P40227	T-complex protein 1 subunit zeta	3				
P40616	ADP-ribosylation factor-like protein 1				Y	Golgi
P40926	Malate dehydrogenase	3	6	Sensitive	Y	Mito
P40939	Trifunctional enzyme subunit alpha	3	5	Sensitive	Y	Mito
P41250	Glycine--tRNA ligase	3				Mito
P41252	Isoleucine--tRNA ligase	3				
P42704	Leucine-rich PPR motif-containing protein	3			Y	Mito
P43034	Platelet-activating factor acetylhydrolase IB subunit alpha	3				
P43243	Matrin-3	3				
P43246	DNA mismatch repair protein Msh2	3				
P43304	Glycerol-3-phosphate dehydrogenase				Y	Mito
P43307	Translocon-associated protein subunit alpha (TRAP-alpha)				Y	ER
P43358	Melanoma-associated antigen 4	3				
P43686	26S protease regulatory subunit 6B				Y	
P45973	Chromobox protein homolog 5		4			
P45974	Ubiquitin carboxyl-terminal hydrolase 5	3				
P46777	60S ribosomal protein L5		5			
P47756	F-actin-capping protein subunit beta	1	5	Sensitive	Y	
P47895	Aldehyde dehydrogenase family 1 member A3	3				
P47897	Glutamine--tRNA ligase	3				Mito
P48147	Prolyl endopeptidase	3				
P48444	Coatomer subunit delta				Y	ER, Golgi
P48637	Glutathione synthetase	3				
P48643	T-complex protein 1 subunit epsilon	3	5	Sensitive	Y	
P48739	Phosphatidylinositol transfer protein beta isoform (PI-TP-beta)				Y	ER, Golgi
P49257	Protein ERGIC-53				Y	ER, Golgi
P49321	Nuclear autoantigenic sperm protein	3				
P49327	Fatty acid synthase	3	4, 7	Sensitive	Y	Golgi, Mito
P49368	T-complex protein 1 subunit gamma		4			
P49411	Elongation factor Tu	3				Mito
P49588	Alanyl-tRNA synthetase	3		PDT specific		
P49591	Serine--tRNA ligase				Y	
P49736	DNA replication licensing factor MCM2	3				
P49755	Transmembrane emp24 domain-containing protein 10				Y	ER, Golgi
P49792	E3 SUMO-protein ligase RanBP2				Y	Mito
P50395	Rab GDP dissociation inhibitor beta	3		PDT specific	Y	Golgi
P50416	Carnitine O-palmitoyltransferase 1				Y	Mito
P50454	Serpin H1				Y	ER, Golgi
P50570	Dynamin-2				Y	Golgi, Mito
P50579	Methionine aminopeptidase 2	3				
P50895	Basal cell adhesion molecule	3			Y	
P50990	T-complex protein 1 subunit theta	3	5		Y	
P50991	T-complex protein 1 subunit delta	3				
P51148	Ras-related protein Rab-5C				Y	
P51572	B-cell receptor-associated protein 31				Y	ER, Golgi, Mito
P52209	6-phosphogluconate dehydrogenase	3				
P52272	Heterogeneous nuclear ribonucleoprotein M		5, 6		Y	
P52566	Rho GDP-dissociation inhibitor 2				Y	
P52701	DNA mismatch repair protein Msh6	3				Golgi
P52888	Thimet oligopeptidase	3				Mito
P53396	ATP-citrate synthase	3				
P53621	Coatomer subunit alpha	3		PDT specific		ER, Golgi
P53999	Activated RNA polymerase II transcriptional coactivator p15		5			
P54577	Tyrosine--tRNA ligase	3				
P54578	Ubiquitin carboxyl-terminal hydrolase 14		5			
P54709	Sodium/potassium-transporting ATPase subunit beta-3				Y	
P54819	Adenylate kinase 2		5			Mito
P54920	Alpha-soluble NSF attachment protein		5			Golgi
P55010	Eukaryotic translation initiation factor 5				Y	
P55072	Transitional endoplasmic reticulum ATPase -ATPase p97 subunit	3			Y	ER
P55084	Trifunctional enzyme subunit beta	3			Y	ER, Mito
P55196	Afadin				Y	

P55735	Protein SEC13 homolog				Y	ER, Golgi
P55786	Puromycin-sensitive aminopeptidase	3				
P56192	Methionine--tRNA ligase	3				
P57088	Transmembrane protein 33				Y	ER
P60174	Triosephosphate isomerase	3				
P60468	Protein transport protein Sec61 subunit beta				Y	ER
P60709	Actin	3				
P60842	Eukaryotic initiation factor 4A-I	3	5	Sensitive	Y	
P60900	Proteasome subunit alpha type-6	3				
P60903	Protein S100-A10				Y	
P61019	Ras-related protein Rab-2A				Y	ER, Golgi
P61163	Alpha-centractin				Y	
P61221	ATP-binding cassette sub-family E member 1	3				Mito
P61247	40S ribosomal protein S3a		4			
P61916	Epididymal secretory protein E1				Y	ER
P61923	Coatomer subunit zeta-1				Y	ER, Golgi
P61978	Heterogeneous nuclear ribonucleoprotein K	3			Y	
P62070	Ras-related protein R-Ras2				Y	ER
P62081	40S ribosomal protein S7				Y	
P62158	Calmodulin				Y	
P62191	26S protease regulatory subunit 4	3	5	Sensitive		
P62195	26S protease regulatory subunit 8				Y	
P62253	Ubiquitin-conjugating enzyme E2 G1				Y	
P62258	14-3-3 protein epsilon	3	4,5	Sensitive	Y	Mito
P62263	40S ribosomal protein S14				Y	Mito
P62330	ADP-ribosylation factor 6				Y	Golgi
P62491	Ras-related protein Rab-11A				Y	Golgi, Mito
P62805	Histone H4				Y	
P62829	60S ribosomal protein L23				Y	
P62873	Guanine nucleotide-binding protein G/G/G subunit beta-1				Y	
P62877	E3 ubiquitin-protein ligase RBX1				Y	
P62879	Guanine nucleotide-binding protein G/G/G subunit beta-2				Y	
P62937	Peptidyl-prolyl cis-trans isomerase A				Y	
P62979	Ubiquitin-40S ribosomal protein S27a	3				
P63244	Guanine nucleotide-binding protein subunit beta-2-like 1		4, 7		Y	Mito
P67809	Nuclease-sensitive element-binding protein 1	3				
P67936	Tropomyosin alpha-4 chain	3				
P68371	Tubulin beta-4B chain	3				
P78330	Phosphoserine phosphatase				Y	
P78344	Eukaryotic translation initiation factor 4 gamma 2				Y	
P78371	T-complex protein 1 subunit beta	3				
P78406	mRNA export factor	3				
P78417	Glutathione S-transferase omega-1	3				
P78527	DNA-dependent protein kinase catalytic subunit	3				
P80303	Nucleobindin-2				Y	ER, Golgi
P83111	Serine beta-lactamase-like protein LACTB				Y	Mito
P83731	60S ribosomal protein L24		5			
P84077	ADP-ribosylation factor 1				Y	Golgi
Q00325	Phosphate carrier protein				Y	Mito
Q00610	Clathrin heavy chain 1		4		Y	Golgi
Q00839	Heterogeneous nuclear ribonucleoprotein U	3	4		Y	
Q01082	Spectrin beta chain				Y	
Q01105	Protein SET	3				ER
Q01130	Splicing factor, arginine/serine-rich 2		5		Y	
Q01518	Adenylyl cyclase-associated protein 1	3		PDT specific	Y	
Q01581	Hydroxymethylglutaryl-CoA synthase	3		PDT specific		
Q01813	ATP-dependent 6-phosphofructokinase	3				
Q02252	Methylmalonate-semialdehyde dehydrogenase	3				Mito
Q02790	Peptidyl-prolyl cis-trans isomerase FKBP4	3			Y	ER, Mito
Q03252	Lamin-B2		5			
Q04637	Eukaryotic translation initiation factor 4 gamma 1	3		PDT specific		
Q04828	Aldo-keto reductase family 1 member C1	3				
Q06203	Amidophosphoribosyltransferase	3				
Q06830	Peroxiredoxin-1	3				Mito
Q07065	Cytoskeleton-associated protein 4				Y	ER
Q09666	Neuroblast differentiation-associated protein AHNAK				Y	

Q10713	Mitochondrial-processing peptidase subunit alpha	3			Y	Mito
Q12769	Nuclear pore complex protein Nup160				Y	
Q12797	Aspartyl/asparaginyl beta-hydroxylase				Y	ER
Q12906	Interleukin enhancer-binding factor 3	3				Mito
Q12907	Vesicular integral-membrane protein VIP36				Y	ER, Golgi
Q12931	Heat shock protein 75 kDa	2		PDT specific		Mito
Q13158	Protein FADD				Y	
Q13162	Peroxiredoxin-4		4, 6			ER, Mito
Q13177	Serine/threonine-protein kinase PAK 2	3				
Q13188	Serine/threonine-protein kinase 3				Y	
Q13200	26S proteasome non-ATPase regulatory subunit 2	3			Y	
Q13217	DnaJ homolog subfamily C member 3				Y	ER
Q13308	Inactive tyrosine-protein kinase 7	3				
Q13409	Cytoplasmic dynein 1 intermediate chain 2	3				
Q13435	Splicing factor 3B subunit 2				Y	
Q13751	Laminin subunit beta-3				Y	
Q13753	Laminin subunit gamma-2				Y	
Q13867	Bleomycin hydrolase	3				
Q14126	Desmoglein-2				Y	
Q14134	Tripartite motif-containing protein 29	3				
Q14137	Ribosome biogenesis protein BOP1	3				
Q14152	Eukaryotic translation initiation factor 3 subunit A		5			
Q14204	Cytoplasmic dynein 1 heavy chain 1	3				
Q14247	Src substrate cortactin	3		PDT specific	Y	Golgi
Q14258	E3 ubiquitin/ISG15 ligase TRIM25	3				
Q14435	Polypeptide N-acetylgalactosaminyltransferase 3				Y	Golgi
Q14444	Caprin-1	3				
Q14566	DNA replication licensing factor MCM6	3				
Q14697	Neutral alpha-glucosidase AB	3		PDT specific	Y	ER, Golgi
Q14847	LIM and SH3 domain protein 1	3				
Q14974	Importin subunit beta-1	3		PDT specific		ER
Q14C86	GTPase-activating protein and VPS9 domain-containing protein 1				Y	
Q15005	Signal peptidase complex subunit 2				Y	ER
Q15046	Lysine--tRNA ligase	3				Mito
Q15056	Eukaryotic translation initiation factor 4H				Y	
Q15084	Protein disulfide-isomerase A6	3				ER, Golgi
Q15181	Inorganic pyrophosphatase	3				
Q15233	Non-POU domain-containing octamer-binding protein				Y	
Q15286	Ras-related protein Rab-35				Y	Mito
Q15365	Poly(rC)-binding protein 1	3	4	Sensitive		
Q15366	Poly(rC)-binding protein 2		4			
Q15392	Delta(24)-sterol reductase				Y	ER, Golgi
Q15393	Splicing factor 3B subunit 3	3				
Q15417	Calponin-3				Y	
Q15437	Protein transport protein Sec23B				Y	ER, Golgi
Q16531	DNA damage-binding protein 1	3				
Q16543	Hsp90 co-chaperone Cdc37	3			Y	
Q16576	Histone-binding protein RBBP7	3				
Q16611	Bcl-2 homologous antagonist/killer				Y	ER, Mito
Q16658	Fascin	3	4, 6	Sensitive		
Q16666	Gamma-interferon-inducible protein 16		5		Y	
Q16881	Thioredoxin reductase 1	3				Mito
Q16891	Mitochondrial inner membrane protein				Y	Mito
Q32MZ4	Leucine-rich repeat flightless-interacting protein 1		5			
Q4KMP7	TBC1 domain family member 10B				Y	
Q53SF7	Cordon-bleu protein-like 1				Y	
Q5VYK3	Proteasome-associated protein ECM29 homolog				Y	ER, Golgi
Q6NUK1	Calcium-binding mitochondrial carrier protein SCaMC-1				Y	Mito
Q6NZI2	Polymerase I and transcript release factor				Y	ER, Mito
Q6P9B6	TLD domain-containing protein KIAA1609				Y	
Q71U36	Tubulin alpha-1A chain	3				
Q7KZF4	Staphylococcal nuclease domain-containing protein 1	3		PDT specific		Mito
Q7L576	Cytoplasmic FMR1-interacting protein 1				Y	
Q7L7V1	Putative pre-mRNA-splicing factor ATP-dependent RNA helicase DHX32				Y	Mito
Q7Z2K6	Endoplasmic reticulum metallopeptidase 1				Y	ER
Q7Z6Z7	E3 ubiquitin-protein ligase HUWE1		5			

Q86UP2	Kinectin	3				ER
Q8IXB1	DnaJ homolog subfamily C member 10	3				ER
Q8N163	Cell cycle and apoptosis regulator protein 2	3				Mito
Q8N1B4	Vacuolar protein sorting-associated protein 52 homolog				Y	Golgi
Q8NBJ7	Sulfatase-modifying factor 2	3			Y	ER
Q8NBS9	Thioredoxin domain-containing protein 5	3	4	Sensitive		ER
Q8NC51	Plasminogen activator inhibitor 1 RNA-binding protein	3		PDT specific	Y	
Q8NE71	ATP-binding cassette sub-family F member 1	3	5	Sensitive		
Q8NI22	Multiple coagulation factor deficiency protein 2				Y	ER, Golgi
Q8TCG1	Protein CIP2A	3				
Q8TCS8	Polyribonucleotide nucleotidyltransferase 1	3				Mito
Q8TCT9	Minor histocompatibility antigen H13				Y	ER, Golgi
Q8WUM0	Nuclear pore complex protein Nup133				Y	
Q8WWA1	Transmembrane protein 40				Y	
Q92499	ATP-dependent RNA helicase DDX1	3				
Q92544	Transmembrane 9 superfamily member 4				Y	Golgi
Q92597	Protein NDRG1				Y	
Q92598	Heat shock protein 105 kDa	3				
Q92734	Protein TFG				Y	Golgi
Q92797	Symplekin		5			
Q92841	Probable ATP-dependent RNA helicase DDX17	3				
Q92890	Ubiquitin fusion degradation protein 1 homolog				Y	
Q93009	Ubiquitin carboxyl-terminal hydrolase 7	3				
Q96A26	Protein FAM162A				Y	Mito
Q96A33	Coiled-coil domain-containing protein 47				Y	ER
Q96CS3	FAS-associated factor 2				Y	ER
Q96HE7	ERO1-like protein alpha	3		PDT specific	Y	ER
Q96IR7	4-hydroxyphenylpyruvate dioxygenase-like protein	3				
Q96JB5	CDK5 regulatory subunit-associated protein 3				Y	
Q96KP4	Cytosolic non-specific dipeptidase	3				
Q96RQ3	Methylcrotonoyl-CoA carboxylase subunit alpha	3				Mito
Q96RS6	NudC domain-containing protein 1				Y	
Q96S66	Chloride channel CLIC-like protein 1				Y	ER, Golgi
Q96TA1	Niban-like protein 1				Y	
Q99436	Proteasome subunit beta type-7	3				
Q99460	26S proteasome non-ATPase regulatory subunit 1	3				
Q99497	Protein deglycase DJ-1	3				ER, Mito
Q99575	Ribonucleases P/MRP protein subunit POP1	3				
Q99798	Aconitate hydratase	3				Mito
Q99832	T-complex protein 1 subunit eta	3				Mito
Q99959	Plakophilin-2				Y	
Q9BQE3	Tubulin alpha-1C chain	1		PDT specific	Y	
Q9BRP8	Partner of Y14 and mag				Y	
Q9BS26	Endoplasmic reticulum resident protein 44				Y	ER, Golgi
Q9BUF5	Tubulin beta-6 chain		4			
Q9BV40	Vesicle-associated membrane protein 8				Y	
Q9BVK6	Transmembrane emp24 domain-containing protein 9				Y	ER, Golgi
Q9BW27	Nuclear pore complex protein Nup85				Y	
Q9BW60	Elongation of very long chain fatty acids protein 1				Y	ER
Q9BYX2	TBC1 domain family member 2A				Y	
Q9H0U4	Ras-related protein Rab-1B				Y	ER, Golgi, Mito
Q9H223	EH domain-containing protein 4				Y	ER
Q9H2P0	Activity-dependent neuroprotector homeobox protein	3				
Q9H3K6	BolA-like protein 2				Y	
Q9H3N1	Thioredoxin-related transmembrane protein 1	3				ER
Q9H4A4	Aminopeptidase B	3				
Q9H4M9	EH domain-containing protein 1		5		Y	
Q9H6Y2	WD repeat-containing protein 55				Y	
Q9H6Z4	Ran-binding protein 3		5			
Q9HAU5	Regulator of nonsense transcripts 2				Y	
Q9HAV7	GrpE protein homolog 1				Y	Mito
Q9HB71	Calcyclin-binding protein	3		PDT specific		
Q9HCY8	Protein S100-A14				Y	
Q9NP81	Serine--tRNA ligase	3				Mito
Q9NQC3	Reticulon-4		4			ER
Q9NQR4	Omega-amidase NIT2	3				Mito

Q9NQW7	Xaa-Pro aminopeptidase 1	3				
Q9NR31	GTP-binding protein SAR1a (COPII-associated small GTPase)				Y	ER, Golgi
Q9NRX4	14 kDa phosphohistidine phosphatase				Y	
Q9NS69	Mitochondrial import receptor subunit TOM22 homolog		5		Y	Mito
Q9NS86	LanC-like protein 2	3		PDT specific	Y	
Q9NSD9	Phenylalanine--tRNA ligase beta subunit	3				
Q9NSE4	Isoleucine--tRNA ligase	3				Mito
Q9NTJ3	Structural maintenance of chromosomes protein 4	3				
Q9NTJ5	Phosphatidylinositide phosphatase SAC1				Y	ER, Golgi
Q9NVI7	ATPase family AAA domain-containing protein 3A		5			Mito
Q9NVJ2	ADP-ribosylation factor-like protein 8B				Y	
Q9NX63	MICOS complex subunit MIC19				Y	Mito
Q9NY33	Dipeptidyl peptidase 3	3				
Q9NYP7	Elongation of very long chain fatty acids protein 5				Y	ER
Q9NYU2	UDP-glucose:glycoprotein glucosyltransferase 1				Y	ER, Golgi
Q9NZ08	Endoplasmic reticulum aminopeptidase 1				Y	ER
Q9NZM1	Myoferlin				Y	
Q9NZT2	Opioid growth factor receptor	3				
Q9P0I2	ER membrane protein complex subunit 3				Y	
Q9P0L0	Vesicle-associated membrane protein-associated protein A				Y	ER, Golgi
Q9P0V9	Septin-10				Y	
Q9P1F3	Costars family protein ABRACL				Y	
Q9P258	Protein RCC2	3				
Q9P265	Disco-interacting protein 2 homolog B				Y	
Q9P2E9	Ribosome-binding protein 1		7		Y	ER
Q9UBI1	COMM domain-containing protein 3				Y	
Q9UBI6	Guanine nucleotide-binding protein G(I)/G(S)/G(O) subunit gamma-12				Y	
Q9UBT2	SUMO-activating enzyme subunit 2	3				
Q9UH65	Switch-associated protein 70	3				
Q9UHB6	LIM domain and actin-binding protein 1	3	5	Sensitive		
Q9UHG3	Prenylcysteine oxidase 1				Y	
Q9UJZ1	Stomatin-like protein 2				Y	Mito
Q9UKY7	Protein CDV3 homolog				Y	
Q9ULA0	Aspartyl aminopeptidase	3				
Q9ULV4	Coronin-1C	3				
Q9UQ80	Proliferation-associated protein 2G4	3	5	Sensitive		
Q9Y230	RuvB-like 2	3				
Q9Y265	RuvB-like 1	3		PDT specific	Y	Golgi
Q9Y2B0	Protein canopy homolog 2				Y	ER
Q9Y2Q3	Glutathione S-transferase kappa 1				Y	Mito
Q9Y371	Endophilin-B1				Y	Golgi, Mito
Q9Y3F4	Serine-threonine kinase receptor-associated protein	3		PDT specific		
Q9Y3Z3	Deoxynucleoside triphosphate triphosphohydrolase SAMHD1	3				
Q9Y490	Talin-1	3				
Q9Y4L1	Hypoxia up-regulated protein 1	3	5		Y	ER
Q9Y5L4	Mitochondrial import inner membrane translocase subunit Tim13				Y	Mito
Q9Y617	Phosphoserine aminotransferase	3				
Q9Y624	Junctional adhesion molecule A				Y	
Q9Y696	Chloride intracellular channel protein 4				Y	Mito

References:

1. Magi B, Ettorre A, Liberatori S, Bini L, Andreassi M, Frosali S, Neri P, Pallini V, Di Stefano A. Selectivity of protein carbonylation in the apoptotic response to oxidative stress associated with photodynamic therapy: a cell biochemical and proteomic investigation. Cell Death Differ. 11(8):842-52, 2004.

2. Lu Y, Jiao R, Chen X, Zhong J, Ji J, Shen P. Methylene blue-mediated photodynamic therapy induces mitochondria-dependent apoptosis in HeLa cell. J Cell Biochem. 105(6):1451-60, 2008.

3. Tsaytler PA, C O'Flaherty M, Sakharov DV, Krijgsveld J, Egmond MR. Immediate protein targets of photodynamic treatment in carcinoma cells. J Proteome Res. 7(9):3868-78, 2008.

4. Martínez-Acedo P, Núñez E, Gómez FJ, Moreno M, Ramos E, Izquierdo-Álvarez A, Miró-Casas E, Mesa R, Rodriguez P, Martínez-Ruiz A, Dorado DG, Lamas S, Vázquez J. A novel strategy for global analysis of the dynamic thiol redox proteome. Mol Cell Proteomics. 11(9):800-13, 2012.

5. Ghesquière B, Jonckheere V, Colaert N, Van Durme J, Timmerman E, Goethals M, Schymkowitz J, Rousseau F, Vandekerckhove J, Gevaert K. Redox proteomics of protein-bound methionine oxidation. Mol Cell Proteomics. 10(5):M110.006866, 2011.

6. Ugarte N, Ladouce R, Radjei S, Gareil M, Friguet B, Petropoulos I. Proteome alteration in oxidative stress-sensitive methionine sulfoxide reductase-

Figure S1

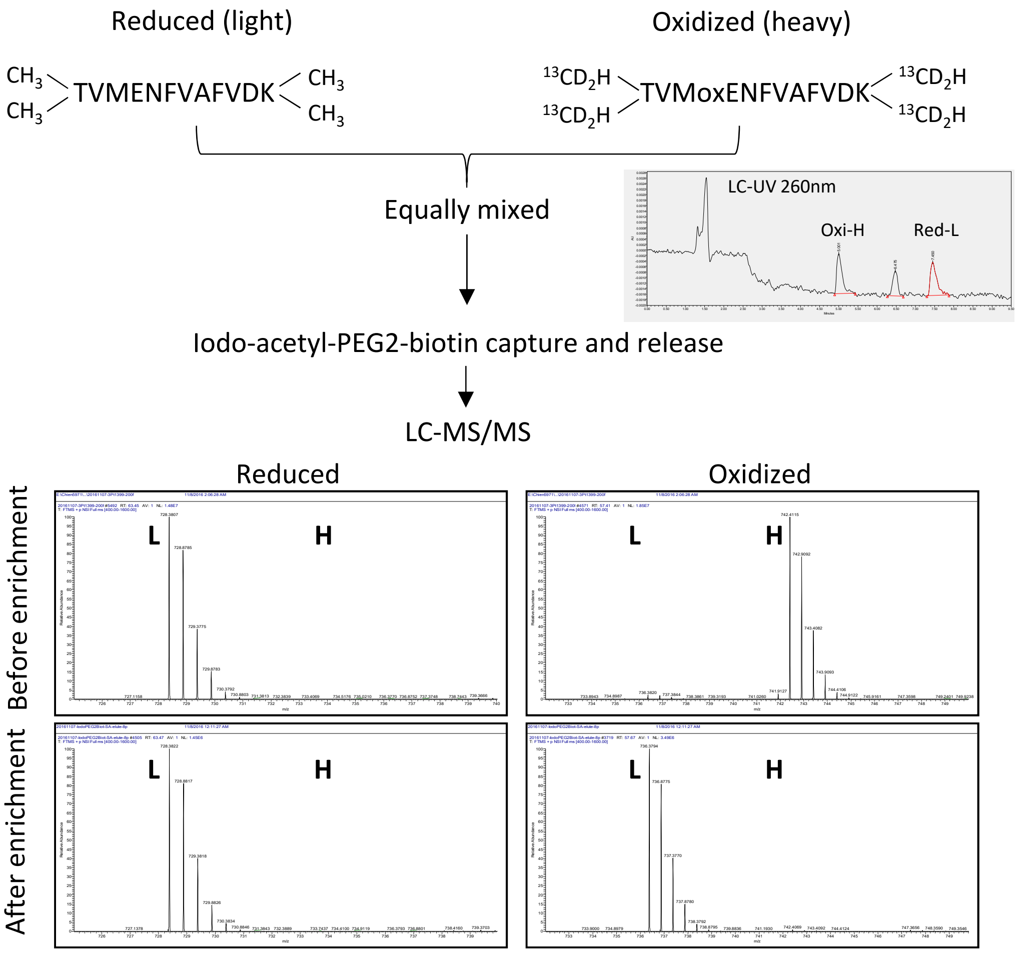

EIC peak area	Reduced		Oxidized	
	Light	Heavy	Light	Heavy
Before capture	195,790,414	ND*	28,536,152	345,065,465
After capture	191,676,497	ND*	55,801,473	8,568,150

*ND: Not detectable

Normalized by ionization factor (1.91)

EIC peak area	Reduced		Oxidized	
	Light	Heavy	Light	Heavy
Before capture	195,790,414 (100%)	ND*	14,940,393 (7.6%)	180,662,547 (92.4%)
After capture	191,676,497 (100%)	ND*	29,215,430 (15.2%)	4,485,942 (2.3%)

*ND: Not detectable

Figure S1. Quantitative analysis of capture efficiency of the iodoacetyl-PEG2-biotin-based protocol for oxidized and reduced forms of a model Met-peptide.

A model Met-peptide (TVMENFVAFVDK) derived from the tryptic digest of bovine serum albumin was dimethylated by heavy ($^{13}CD_2O$) or light (CH_2O) formaldehyde, and the resulting heavy peptide was oxidized by H_2O_2 to generate oxidized heavy peptide. Both oxidized (H) and reduced (L) peptides were purified by HPLC, quantified by LC-UV, equally mixed and then captured by the iodoacetyl-PEG2-biotin-based protocol. The enriched Met-peptides were released and analyzed by LC-MS/MS. A small portion of the equally mixed peptides was also analyzed by LC-MS/MS before the capture experiment. The peak area of extracted ion chromatograms (EIC) of the reduced and oxidized light/heavy peptides detected in LC-MS/MS were used to evaluate the relative capture efficiency between reduced and oxidized peptides. Since the oxidized and reduced Met-peptides were equally mixed according to the UV quantitation result, the relative ionization efficiency between reduced and oxidized peptides can be estimated from equation A (see below), which indicates that the ionization efficiency of oxidized form is ~1.9 times better than that of the reduced form.

$$\frac{\text{Oxidized (L) + Oxidized (H)}}{\text{Reduced (L) + Reduced (H)}} = \frac{28536152 + 345065465}{195790414 + \quad 0} = 1.91 \ \textbf{(equation A)}$$

After normalization using the ionization factor (1.91), the normalized EIC peak area can now be used to calculate the relative molar ratio between reduced peptide (L), oxidized peptide (L) and oxidized peptide (H) detected in the same LC run, either before or after the capture process.

Before the capture process, a small fraction of the original reduced peptide (L) (taken as 100%) has already converted to oxidized peptide (L) (7.6%) via spontaneous oxidation, which co-eluted with the oxidized peptide (H) (92.4% relative to 100% assigned for the reduced peptide (L)) in the LC run.

After the capture/elution process, three kinds of peptides could be detected: reduced peptide (L), oxidized peptide (L) and oxidized peptide (H), with a molar ratio of 100 : 15.2 : 2.3. This analysis clearly indicated that only a very small fraction (~2.3%) of oxidized peptide (H) could be captured by this protocol as compared to its reduced form (i.e. the reduced peptide (L)), probably due to non-specific interaction. Furthermore, this analysis also revealed that most of the oxidized peptide detected after the capture/elution process was derived from the spontaneous oxidation of the original reduced peptide (L) that had been captured by the iodoacetyl-PEG2-biotin-streptavidin beads.

Figure S2

(a)

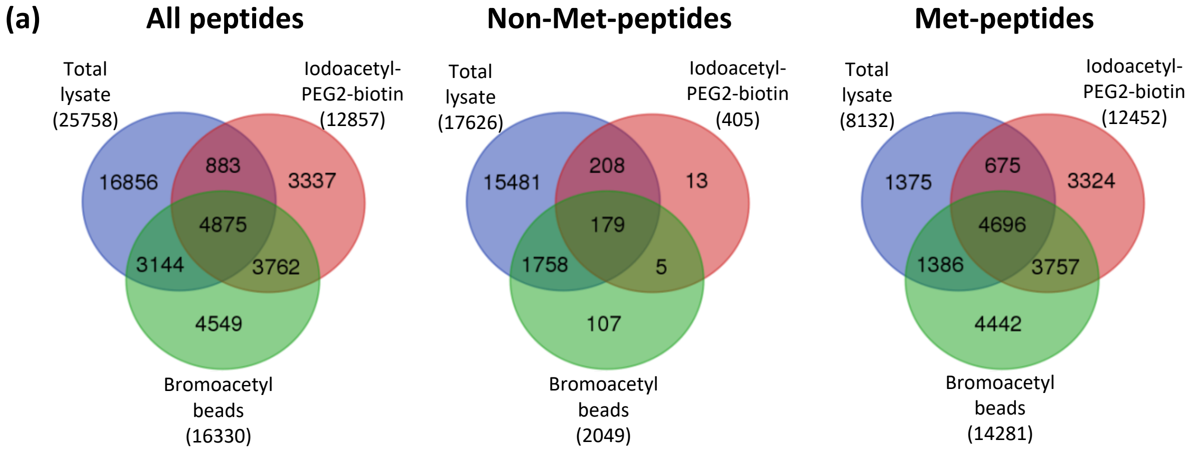

	Total lysate	Enriched by iodoacetyl-PEG2-biotin	Enriched by bromoacetyl
No. of peptides	25758	12857	16330
No. of Met-peptides	8132	12452	14281
No. of non-Met-peptides	17626	405	2049
% of Met-peptides	31.6	96.9	87.5

(b)

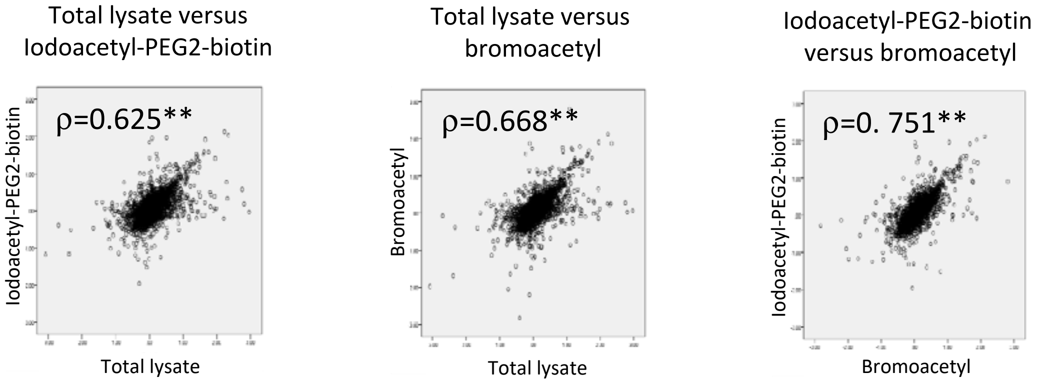

Figure S2. Comparisons between the two protocols used to enrich Met-peptides from lysates of PDT-treated A431 cells. SILAC labeled A431 cells were treated with condition I PDT (H/L=Ctrl/PDT), and the cell lysates were completely digested by trypsin. Tryptic digests (200 μg protein) were subjected to Met-peptide enrichment using the iodoacetyl-PEG2-biotin- and bromoacetyl-based protocols, respectively. After enrichment, one-fourth of the enriched product (equivalent to 50 μg protein of the original lysates) was analyzed by LC-MS/MS for peptide identification and quantitation. The tryptic digests (10 μg protein) without prior enrichment step were also analyzed by LC-MS/MS for comparison purpose. **(a)** Venn diagrams of all peptides, non-Met-peptides and Met-peptides identified among the three samples. **(b)** The ratios (Heavy/Light= Ctrl/PDT) of 3964 reduced Met-peptides simultaneously quantified in all three samples were subjected to Spearman's correlation analysis. ρ, Spearman's rho; **, p value < 0.01.

Figure S3

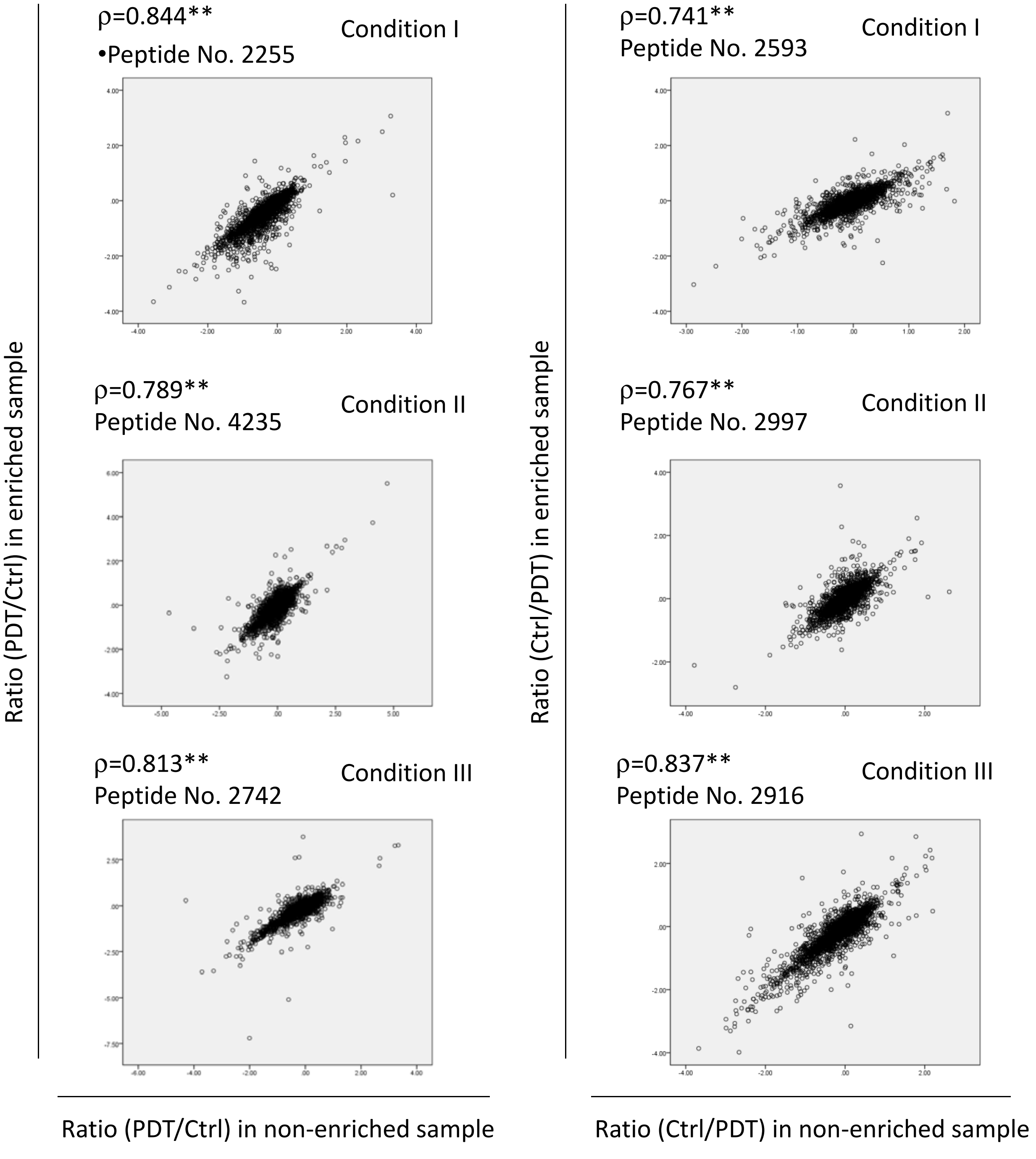

Figure S3. Spearman's correlation coefficient analysis of the ratios of reduced Met-peptides from non-enriched versus enriched samples. The ratios of reduced Met-peptides (Light/Heavy = PDT/Ctrl; Light/Heavy = Ctrl/PDT) simultaneously quantified from both non-enriched and enriched samples were calculated. ρ, Spearman's rho; **, p value < 0.01

Figure S4

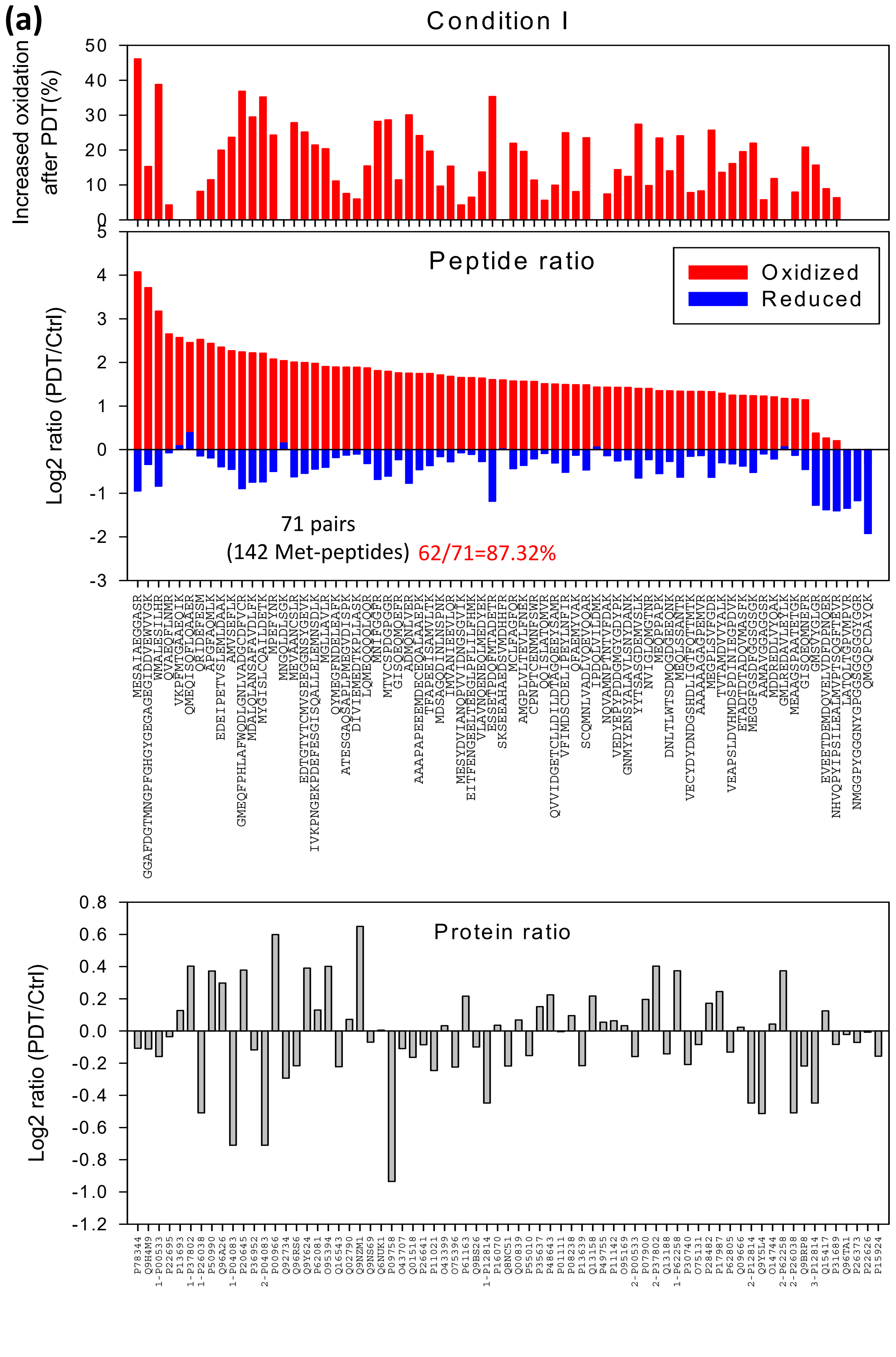
(a)
Condition I
Increased oxidation after PDT(%)
Peptide ratio
Oxidized
Reduced
Log2 ratio (PDT/Ctrl)
71 pairs
(142 Met-peptides)
62/71=87.32%
Protein ratio
Log2 ratio (PDT/Ctrl)

Figure S4

(b)

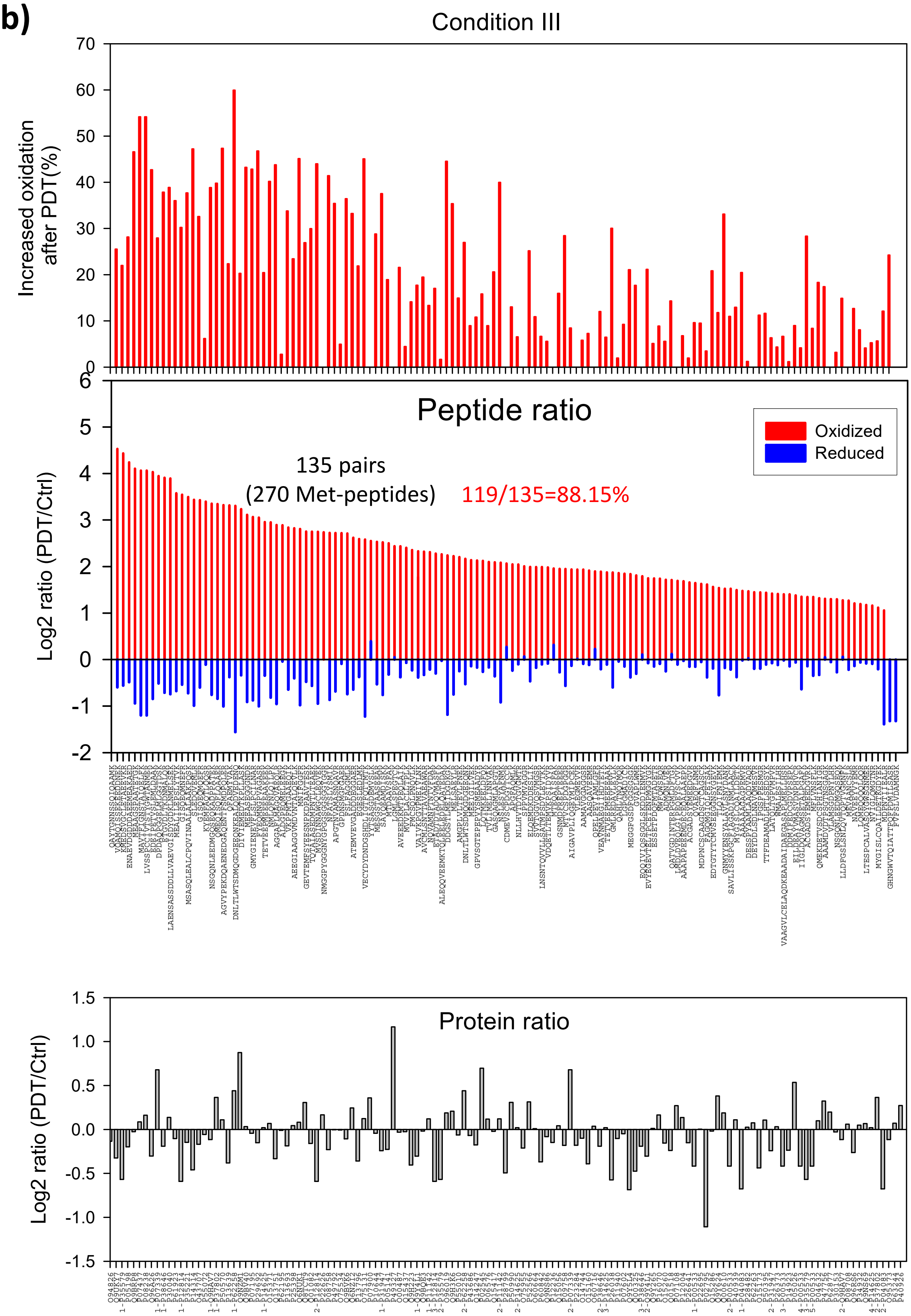

Condition III
Increased oxidation after PDT(%)
0
10
20
30
40
50
60
70
Peptide ratio
135 pairs
(270 Met-peptides)
119/135=88.15%
Oxidized
Reduced
Log2 ratio (PDT/Ctrl)
-2
-1
0
1
2
3
4
5
6
Protein ratio
Log2 ratio (PDT/Ctrl)
-1.5
-1.0
-0.5
0.0
0.5
1.0
1.5

Figure S4

(c)

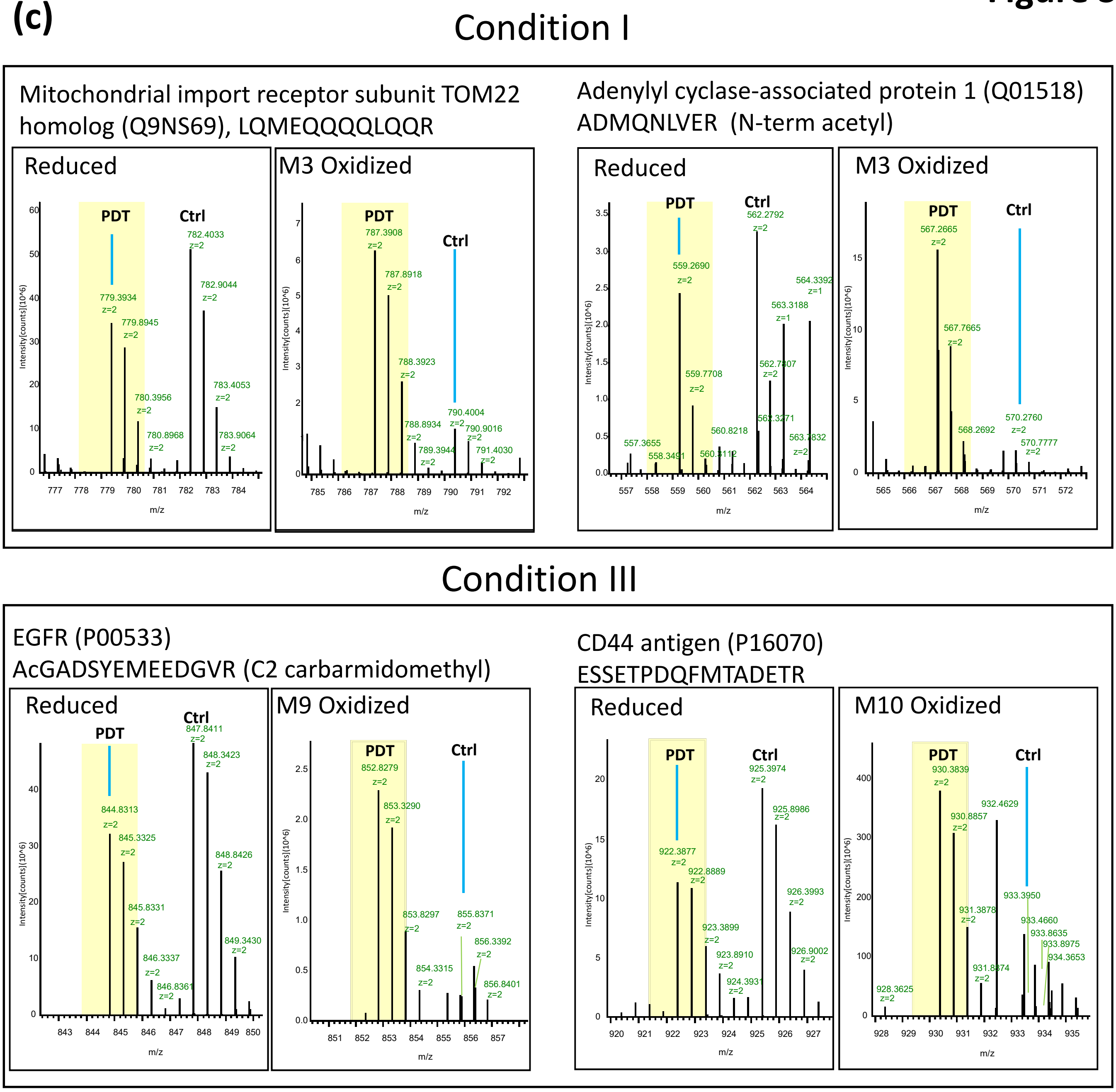

Figure S4. Reciprocal relationship between the increase of oxidized Met-peptides and the decrease of reduced Met-peptides in A431 cells treated with Photofrin-PDT. (A, B) 71 Met-peptide pairs in condition I **(a)** and 135 in condition III **(b)** were used for this analysis. (*Upper panel*): The oxidation percentage of each Met-peptide was calculated according to the equation described in Table S5 and averaged between swapping experiments. (*Middle panel*): The log2 ratios (PDT/Ctrl, in average of swapping experiments) of oxidized and reduced Met-peptides for each Met-peptide pair are shown for comparison. Approximately 87% (62/71) Met-peptide pairs in condition I and 88% (119/135) in condition III showed a good reciprocal relationship between the increase of oxidized Met-peptides and the decrease of reduced Met-peptides. (*Lower panel*): The alteration of the amounts of proteins that comprise the oxidized Met-peptides described above were analyzed according to the PDT/Ctrl ratios derived from their non-Met-peptides. **(c)** Representative MS spectra for selected Met-peptides containing both oxidized and reduced forms are shown to illustrate the reciprocal relationship.

Figure S5

(a)

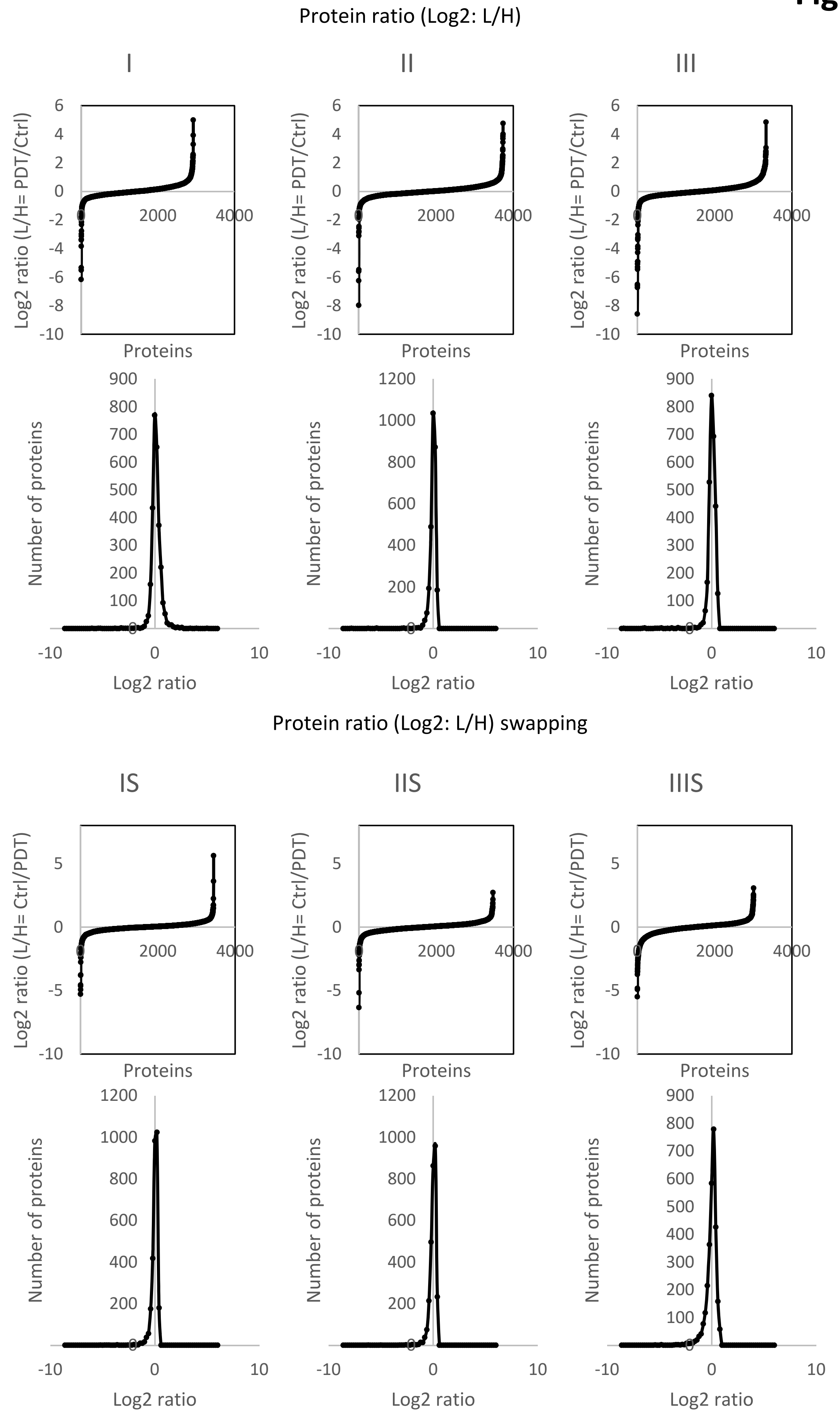
Protein ratio (Log2: L/H)
I
II
III
Log2 ratio (L/H= PDT/Ctrl)
6
4
2
0
-2
-4
-6
-8
-10
0
2000
4000
Proteins
Number of proteins
900
800
700
600
500
400
300
200
100
1200
1000
-10
10
Log2 ratio
Protein ratio (Log2: L/H) swapping
IS
IIS
IIIS
Log2 ratio (L/H= Ctrl/PDT)
5
-5

Figure S5

(b)

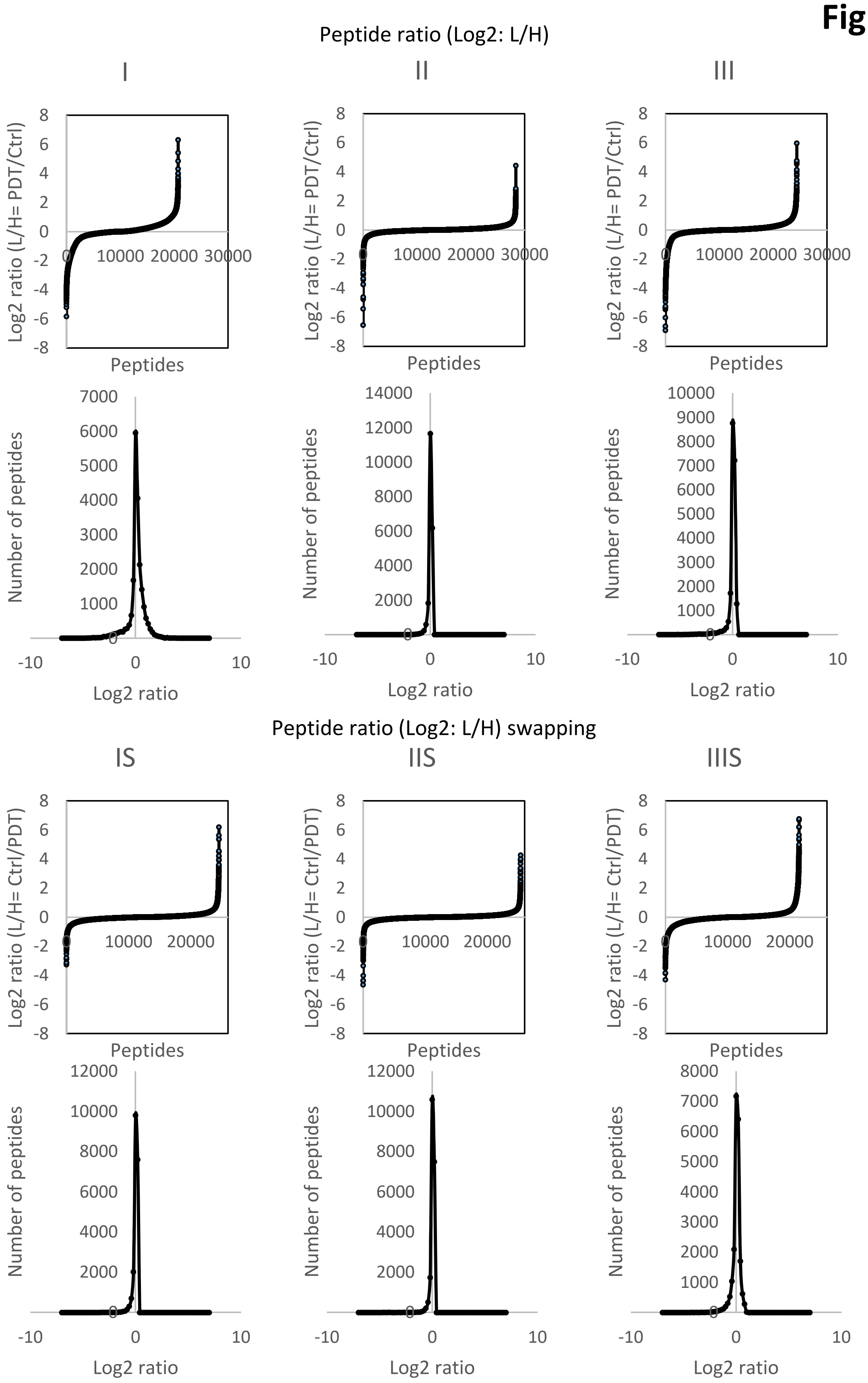

Figure S5. Global distribution of the protein/peptide ratio (Log2) determined by LC-MS/MS in the six non-enriched samples. (A) Protein ratio distribution excluding all Met-containing peptides. (B) Peptide ratio distribution.
